# Multitarget Design of Steroidal Inhibitors Against Hormone-Dependent Breast Cancer: An Integrated In Silico Approach

**DOI:** 10.3390/ijms26157477

**Published:** 2025-08-02

**Authors:** Juan Rodríguez-Macías, Oscar Saurith-Coronell, Carlos Vargas-Echeverria, Daniel Insuasty Delgado, Edgar A. Márquez Brazón, Ricardo Gutiérrez De Aguas, José R. Mora, José L. Paz, Yovanni Marrero-Ponce

**Affiliations:** 1 Facultad de Ciencias de la Salud, Exactas y Naturales, Universidad Libre, Barranquilla 080001, Colombia; 2 Grupo de Investigaciones en Química y Biología, Departamento de Química y Biología, Facultad de Ciencias Básicas, Universidad del Norte, Carrera 51B, Km 5, Vía Puerto Colombia, Barranquilla 081007, Colombia; osaurith@uninorte.edu.co (O.S.-C.); vargasce@uninorte.edu.co (C.V.-E.); insuastyd@uninorte.edu.co (D.I.D.); ebrazon@uninorte.edu.co (E.A.M.B.); rgutierr@uninorte.edu.co (R.G.D.A.); 3 Grupo de Química Computacional y Teórica (QCT-USFQ), Departamento de Ingeniería Química, Universidad San Francisco de Quito, Diego de Robles y Vía Interoceánica, Quito 170901, Ecuador; jrmora@usfq.edu.ec; 4 Departamento Académico de Química Inorgánica, Facultad de Química e Ingeniería Química, Universidad Nacional Mayor de San Marcos, Lima 15081, Peru; jpazr@unmsm.edu.pe; 5 Facultad de Ingeniería, Universidad Panamericana, Augusto Rodin No. 498, Insurgentes Mixcoac, Benito Juárez, Ciudad de México 03920, Mexico

**Keywords:** QSAR, steroids, docking, HER2, ERα, PR, inhibitors

## Abstract

Hormone-dependent breast cancer, particularly in its treatment-resistant forms, remains a significant therapeutic challenge. In this study, we applied a fully computational strategy to design steroid-based compounds capable of simultaneously targeting three key receptors involved in disease progression: progesterone receptor (PR), estrogen receptor alpha (ER-α), and HER2. Using a robust 3D-QSAR model (R^2^ = 0.86; Q^2^_LOO = 0.86) built from 52 steroidal structures, we identified molecular features associated with high anticancer potential, specifically increased polarizability and reduced electronegativity. From a virtual library of 271 DFT-optimized analogs, 31 compounds were selected based on predicted potency (pIC_50_ > 7.0) and screened via molecular docking against PR (PDB 2W8Y), HER2 (PDB 7JXH), and ER-α (PDB 6VJD). Seven candidates showed strong binding affinities (ΔG ≤ −9 kcal/mol for at least two targets), with Estero-255 emerging as the most promising. This compound demonstrated excellent conformational stability, a robust hydrogen-bonding network, and consistent multitarget engagement. Molecular dynamics simulations over 100 nanoseconds confirmed the structural integrity of the top ligands, with low RMSD values, compact radii of gyration, and stable binding energy profiles. Key interactions included hydrophobic contacts, π–π stacking, halogen–π interactions, and classical hydrogen bonds with conserved residues across all three targets. These findings highlight Estero-255, alongside Estero-261 and Estero-264, as strong multitarget candidates for further development. By potentially disrupting the PI3K/AKT/mTOR signaling pathway, these compounds offer a promising strategy for overcoming resistance in hormone-driven breast cancer. Experimental validation, including cytotoxicity assays and ADME/Tox profiling, is recommended to confirm their therapeutic potential.

## 1. Introduction

Cancer is a genetic disease that alters cellular function and constitutes a global public health problem. In 2020, approximately 10 million deaths were attributed to various types of cancer [1], some of which are more aggressive than others, such as breast, lung, colon, rectum, prostate, skin, and stomach cancers, with significant incidence both nationally and globally [1,2,3,4,5]. Among these cancer types, breast cancer is one of the most impactful types on public health and one of the leading causes of mortality in women worldwide. The World Health Organization (WHO) estimated that in 2020, more than 2.2 million new cases were diagnosed, and over 685,000 deaths occurred due to breast cancer [6]. In low- and middle-income countries, where most cases are diagnosed in advanced stages, breast cancer is the leading cause of cancer death in women, accounting for 25% of all cancer cases and the second cause of cancer-related deaths in women in Latin America [7].

Despite advances in early detection and treatment, breast cancer remains a significant cause of mortality. Current therapies, such as chemotherapy and radiotherapy, although effective, can cause severe side effects and reduce patients’ quality of life [8]. Moreover, resistance to existing treatments and the lack of effective drugs pose significant challenges for medical and pharmaceutical research [9,10].

The molecular classification of breast cancer has enabled the identification of subtypes with specific biological, clinical, and prognostic characteristics, including luminal A, associated with a favorable prognosis; luminal B; HER2-positive; and triple-negative, which is linked to a poorer prognosis. These subtypes are defined by the expression of hormonal receptors and HER2, facilitating the selection of personalized treatments [11,12,13].

Steroid hormones, such as androgens and estrogens, play a crucial role in breast cancer progression. Tumors expressing estrogen receptors have been shown to respond to anti-estrogen therapies, such as tamoxifen, or aromatase inhibitors. Understanding the mechanisms of action of steroid hormones is essential for the development of more effective and targeted therapies [14,15,16].

In the search for new treatments, quantitative structure-activity relationship (QSAR) modeling has become a key approach for identifying and refining potential anticancer compounds. It offers a faster, more cost-effective alternative to traditional lab-based methods by helping researchers predict which molecules are most likely to be effective [17,18,19]. Among the many candidates explored, steroid-based compounds have stood out due to their favorable properties—such as low toxicity and good bioavailability. Several studies have highlighted their ability to interact with key enzymes and receptors involved in cancer progression, making them strong contenders in the fight against breast cancer [20,21,22,23]. In this context, in silico tools have revolutionized the early stages of drug discovery, offering a powerful and flexible platform to explore chemical space with unprecedented speed and precision. Techniques such as QSAR modeling, molecular docking, and molecular dynamics simulations allow researchers to not only predict biological activity but also visualize and understand how potential drugs interact with their targets at the atomic level. These tools reduce the need for costly and time-consuming experimental screening, enabling a more focused and rational approach to drug design [24,25,26,27].

Computational tools are playing an increasingly important role in drug development. A good example is raloxifene, a selective estrogen receptor modulator (SERM) commonly used to help prevent breast cancer. Researchers have used structure-based design methods, along with advanced drug delivery systems like nanocarriers, to improve how well raloxifene dissolves, how much of it the body can absorb, and how effectively it works against cancer cells in lab studies [24,28]. Along the same lines, abiraterone, a steroidal CYP17 inhibitor primarily indicated for the treatment of prostate cancer, has been refined through computational pharmacokinetic modeling. This optimization has enhanced its metabolic stability, thereby permitting reduced dosing regimens and contributing to improved therapeutic outcomes [29].

More recently, GDC-0810 (brilanestrant), an orally bioavailable selective estrogen receptor degrader (SERD), exemplifies the integration of computational modeling with experimental validation to accelerate drug development. This compound was iteratively optimized using in silico approaches to enhance estrogen receptor (ER) degradation, antagonistic activity, and pharmacokinetic properties. These improvements were subsequently confirmed through in vivo studies and early-phase clinical trials, demonstrating its potential efficacy in patients with ER-positive, HER2-negative advanced breast cancer [30,31].

Given the persistent global rise of hormone-dependent breast cancer, especially in areas with limited healthcare infrastructure, both innovative targeted therapies have never been more critical. Here, we describe a comprehensive computational pipeline that combines QSAR modeling, molecular docking, and molecular dynamics simulations to design new steroidal anticancer agents. Our goal is to harness in silico strengths to elucidate key structure–activity relationships, predict binding affinity and stability, and ultimately lay the groundwork for experimental follow-up. By doing so, we aim not only to advance scientific insight but also to contribute toward accessible, effective treatments for breast cancer that can translate into real-world clinical impact.

## 2. Results

To obtain multitarget compounds, a logical sequence was followed for analyzing basic features, designing, and screening the ligands with the best profiles. The workflow began with the QSAR model and proceeded to molecular docking analyses. Next, new ligands were designed by incorporating the modifications suggested by the model and their interactions with the three receptors. These ligands were screened based on both their molecular interaction profiles and the biological activity predicted by the QSAR model. The top-performing ligands were then evaluated through 100 ns molecular dynamics simulations and ADME/Tox studies.

### 2.1. Selection of Training Data

After applying the selection criteria, we gathered a total of 52 steroidal compounds with documented anticancer activity against breast cancer cell lines. These compounds were sourced from both scientific literature and specialized chemical databases, as detailed in Table 1. For clarity and ease of reference, each molecule was labeled sequentially as Mol-n. To standardize the biological activity data, we used the IC_50_ values reported in the original studies, which represent the concentration (in mol/L) required to inhibit 50% of cancer cell growth. To make the data more suitable for computational modeling, we converted these values into their negative logarithmic form, known as pIC_50_ (i.e., pIC_50_ = −log_10_[IC_50_]). This transformation helps to linearize the data and is commonly used in QSAR studies. Additionally, we recorded the SMILES (Simplified Molecular Input Line Entry System) notation for each compound to digitally represent their chemical structures (see Supplementary Material Table S1).

These molecules were optimized using DFT calculations with the LanL2DZ basis set, and the minimum energy structures were confirmed by means of the frequency calculations; compounds with all secondary derivatives >0 were considered “optimized”. These optimized molecules were used to compute more than 800 molecular descriptors; t Multiple correlation analyses were performed to select a mathematical model with eight descriptors, seven of which were primarily algebraic, while one corresponded to the number of HBond donors (Table 2).

### 2.2. QSAR Modeling

Following the molecular geometry optimization, a comprehensive set of molecular descriptors was calculated, including electronic, thermodynamic, and topological properties. These descriptors were used as independent variables, while the experimentally determined anticancer activity, expressed as pIC_50_, served as the dependent variable.

To identify the most predictive relationships between molecular structure and biological activity, various statistical modeling techniques were applied. These included regression-based approaches aimed at constructing robust mathematical models capable of correlating the calculated descriptors with the observed pIC_50_ values.

Although multiple models were generated during this process, the most statistically significant and predictive model was selected for further analysis. This final model is presented in Equation (1), and the corresponding descriptor labels are summarized in Table 2.

pICL50 = 10.956 − 3.525*A* − 4.2412*B* + 0.5453*C* − 0.024*D* + 0.1555*E* − 0.158*F* − 0.264*G* + 0.512*H* N = 52, R^2^ = 0.86, Adj R^2^ = 0.83 (1)

Most of the descriptors used in the model were generated using the QuBiLS-MIDAS software (https://tomocomd.com/software/qubils-midas, accessed on 20 January 2025), which can produce complex molecular descriptors based on electronic, thermodynamic, or topological properties. These descriptors are derived from linear, bilinear, or quadratic algebraic operations, resulting in stochastic matrices, simple stochastic matrices, mutual probability matrices, or non-stochastic matrices that encode molecular structural information. Notably, descriptors 2, 3, 4, and 6 are primarily based on stochastic matrices, while descriptors 1, 7, and 8 rely on non-stochastic matrices (see Table 2).

### 2.3. QSAR Validation

**Figure 1:**
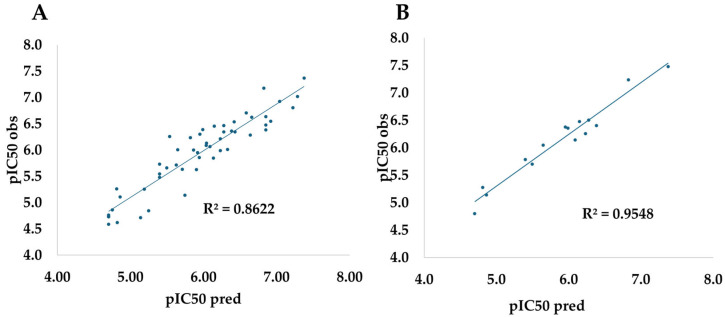
Scatter plot of the observed versus phase-predicted activity (pIC50) through leave-one-out cross-validation (**A**) and Hold-Out (**B**).

Figure 1 shows that Model 1 tracks the experimental pIC_50_ values in an almost perfectly linear fashion, but its true reliability emerges in the validation phase. With leave-one-out cross-validation (LOOCV), which recalibrates the model 52 times and predicts each compound only once, the model delivers a Q^2^ of 0.861, virtually identical to the adjusted R^2^ (0.862), alongside very low errors (RMSEP = 0.274 and MAE = 0.218 log units). Tropsha’s tests further rule out systematic bias: the origin-forced slopes k and k′ are around 0.93, and all ΔR^2^ values are below 0.08. These figures point to high internal robustness and a minimal risk of overfitting (see Table 3).

A 15-compound Hold-Out test likewise confirms the model’s extrapolative power (R^2^_ext = 0.826, RMSEP = 0.301, MAE = 0.253), yet unlike LOOCV, it does not probe the influence of every single data point on the model. The close match between both metric sets boosts confidence, but the LOOCV results carry greater weight because they exploit almost the entire chemical space during training, evaluate every molecule, and still meet the most stringent thresholds (|R^2^ − Q^2^| < 0.1; 0.85 < k, k′ < 1.15) [32]. Both validation schemes support the predictive reliability of Model 1, yet the LOOCV analysis provides the most compelling evidence of its robustness for predicting the anticancer activity of new steroid derivatives.

### 2.4. New Anticancer Molecules Prediction

To improve the anticancer potential of steroid-based compounds, we introduced specific chemical modifications aimed at enhancing their electronic properties. One of the key strategies was to increase the molecule’s polarizability, its ability to shift its electron cloud in response to the environment, by adding highly polarizable or electronegative groups like carbonyl (C=O), bromine (Br), nitro (NO_2_), sulfoxide (SO), and sulfone (SO_2_). These groups help the molecule better adapt to the dynamic conditions of a biological target, which can strengthen binding and improve activity [33]. At the same time, we added functional groups like hydroxyl (–OH) or amino (–NH_2_) to increase the number of hydrogen bond donors, which are known to support stronger interactions with receptors. These changes were guided by the QSAR model that showed a clear positive relationship between these features and anticancer activity, specifically through descriptors labeled C, E, and H. We also avoided bulky or rigid groups that tend to reduce activity, as indicated by negatively correlated descriptors (A, B, D, F, and G).

### 2.5. Combining Molecular Docking with QSAR to Design New Anticancer Molecules

After completing the QSAR analysis, we decided to take a closer look at how our designed compounds might interact with real biological targets. To do this, we carried out molecular docking studies against three key proteins involved in hormone-related cancers: the progesterone receptor, the HER2 tyrosine kinase domain, and estrogen receptor-α. These proteins were chosen because of their well-established roles in cancer progression and treatment. As a benchmark, we used three known antagonists—Mifepristone, Lapatinib, and Raloxifene—which are clinically validated inhibitors for each respective target.

By combining QSAR modeling with molecular docking, we were able to bridge the gap between statistical prediction and structural insight. QSAR helped us identify which molecular features are most likely to contribute to anticancer activity, while docking allowed us to visualize how these compounds might bind to their targets. This combination not only strengthens the reliability of our predictions but also gives us a clearer picture of the types of interactions—like hydrogen bonds or hydrophobic contacts—that are essential for strong and selective binding.

This integrated approach has been gaining traction in drug discovery because it offers a more efficient and informed way to design new molecules. Instead of relying solely on trial-and-error or high-throughput screening, we can now prioritize compounds with a higher chance of success based on both their predicted activity and their structural compatibility with the target. Recent studies have shown that this strategy can significantly improve hit rates and lead optimization in anticancer drug development [34,35].

**Figure 2:**
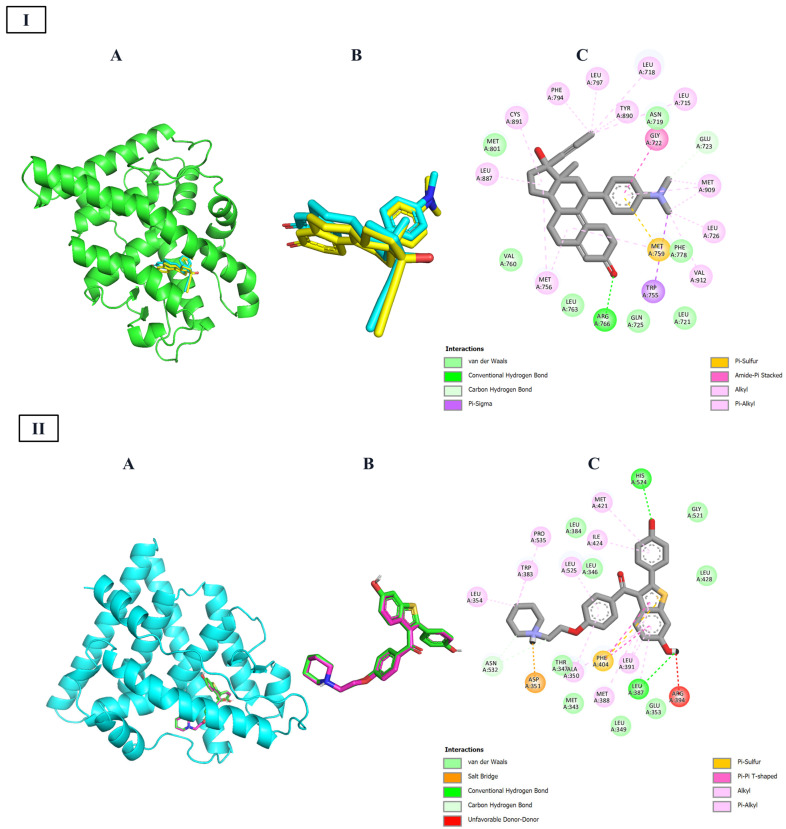

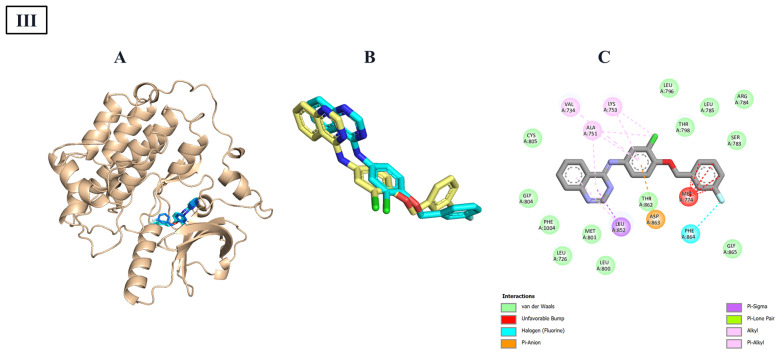
Validation of the redocking protocol for the native receptor-inhibitor complexes: (**A**) ribbon representation of the target protein, (**B**) superposition of the crystallographic (yellow) and redocked (cyan) inhibitor conformations, and (**C**) 2D interaction map of the redocked pose. **I**: The progesterone-activated transcription factor nuclear receptor with Mifepristone. **II**: The estrogen-activated transcription factor nuclear receptor with Raloxifene. **III**: Tyrosine protein kinase receptor erbB-2 with Lapatinib.

For this reason, to jointly assure the reproducibility of these methods, a redocking protocol was carried out using the ligands against the target, respectively. The graphic results are shown in Figure 2. As revealed in this figure, the redocking protocol can reproduce closely the experimental values, as shown for the small rmsd in each case (<2 Å). Once the redocking protocol is validated, all data were docked against three targets and compared with the inhibitor compounds. The results are shown in Table 4. Compounds with equal or higher values were considered as the baseline for further modifications.

**Figure 3:**
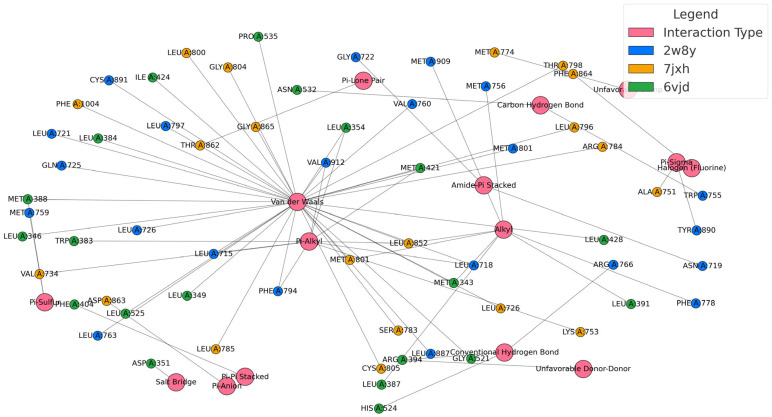
Network of ligand–protein non-covalent interactions in complexes 2W8Y, 7JXH, and 6VJD, classified by contact type and participating residue.

The molecular docking results provide a valuable layer of validation for the QSAR model, offering a structural explanation for the electronic and topological descriptors that were statistically linked to anticancer activity. By analyzing the interactions between the designed steroidal compounds and the three selected targets (summarized in Table 5 and shown in Figure 2 and Figure 3), we observed a consistent pattern of hydrophobic and π-type interactions, particularly with residues such as leucine, methionine, and phenylalanine. These findings align closely with the QSAR descriptors C and H, which were positively correlated with activity and are associated with molecular features that enhance polarizability and electronic adaptability.

The presence of van der Waals, alkyl, and π–alkyl interactions supports the inclusion of hydrocarbon rings and alkyl chains, structural elements that contribute to the electronic descriptors captured by the QuBiLS-MAS model. Similarly, the observed π–π stacking and π–sigma interactions reinforce the importance of aromatic or unsaturated systems, which not only stabilize the ligand–receptor complex but also reflect the electronic delocalization patterns that the QSAR model identified as favorable.

Moreover, the docking studies highlighted the role of hydrogen bonding and salt bridge formation, particularly with polar and charged residues like aspartate and arginine. These interactions are consistent with the positive contribution of descriptor E (number of hydrogen bond donors) in the QSAR equation, confirming that functional groups capable of forming such bonds are critical for strong and specific binding.

Interestingly, interactions such as π–sulfur and halogen bonding, observed in complexes involving sulfur-containing or halogenated substituents, further validate the inclusion of these groups as a strategy to enhance binding affinity and specificity. In fact, their role in improving ligand–receptor interactions has been well documented before [36]. These features are not only chemically intuitive but also statistically supported by the QSAR model, which penalized descriptors associated with rigid or sterically hindered structures (A, B, D, F, G).

All these analyses derived from the obtained model were considered in the design of new steroidal molecules, aiming to enhance their biological activity. Various structures were designed by introducing functional groups according to the guidelines established by the QSAR model. Additionally, these designs incorporated molecular fragments that enhance the interactions identified in the molecular docking studies conducted with inhibitors of the Progesterone-activated transcription factor nuclear receptor, Estrogen-activated transcription factor nuclear receptor, and tyrosine protein kinase erbB-2 receptor, which are key targets in the progression of breast cancer.

**Figure 4:**
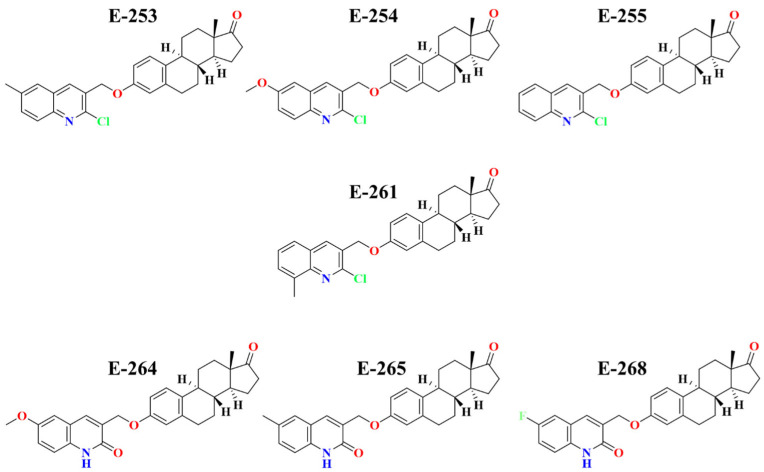
Two-dimensional chemical structures of the seven top-ranked designed steroidal ligands (Estero-253, Estero-254, Estero-255, Estero-261, Estero-264, Estero-265, and Estero-268).

A total of 266 compounds were designed and subsequently optimized at the DFT level using the LanL2DZ basis set and the WB97XD functional (Supplementary Material Table S2). These compounds were then evaluated using the QSAR model, leading to the selection of compounds with the highest predicted biological activity. These compounds were further analyzed through molecular docking simulations with the selected target proteins. A matrix was generated based on interaction energy values, and machine learning techniques were applied to refine the selection, (Supplementary Material S3) ultimately identifying the seven most promising compounds (Supplementary Material Figures S1–S3). Docking scores are reported against three breast cancer targets: progesterone receptor (PR, PDB 2W8Y), HER2 tyrosine kinase domain (PDB 7JXH), and estrogen-receptor-α ligand-binding domain (PDB 6VJD), together with pIC_50_ values predicted by the QSAR model (pIC_50_ = −log_10_ IC_50_, M). The dataset comprises the reference compound RM-581, two clinically used steroidal inhibitors (Exemestane and Formestane), and the seven designed ligands (Estero-253 to Estero-268) that achieved the highest docking scores and the best QSAR-predicted biological activity (Table 6). The molecular 2D structures are shown below in Figure 4.

### 2.6. ADME-TOX Properties

To assess the drug-likeness properties as well as the safety of the predicted compounds, the SMILES codes for the seven top-ranked designed compounds were used to determine their ADME-Tox values. For this goal, ProTox 3.0 software was used https://tox.charite.de/protox3/, accessed 5 June 2025), and the results are shown in Table 7. The in silico assessment of the steroid candidate analogs (Table 8): E-253, E-254, E-255, E-261, E-264, E-265 and E-268, confirms that, aside from the occasional minor deviation, they all satisfy the classical drug-likeness criteria: molecular weights range from 441 to 476 g mol^−1^, each molecule contains at most four rotatable bonds and no more than four hydrogen-bond acceptors. Lipophilicity spans from Log *p* = 4.8 for E-264 to 6.1 for E-253 and E-261, a gradient that is mirrored in solubility; indeed, E-264 offers the best compromise (Log S = −5.50), whereas E-253 and E-261 show the lowest solubilities (≈−6.97). Five of the seven compounds (E-254, E-255, E-264, E-265, and E-268) are predicted to have high gastrointestinal absorption and thus emerge as promising oral candidates, in contrast to E-253 and E-261, whose high log *p* values are associated with slightly limited absorption and therefore call for specialized formulation strategies.

From a toxicological standpoint, compounds E-253, E-255, and E-261 tested positive in the Ames assay, indicating potential mutagenic risk. This suggests that future structural optimization should consider reducing aromatic electron density or exploring prodrug strategies to mask reactive moieties during systemic circulation [37]. In contrast, E-264 is flagged for hepatotoxicity, yet its favorable balance between lipophilicity and solubility suggests that this liability could be addressed through targeted modification, such as fine-tuning metabolic soft spots or introducing steric shields to reduce bioactivation [38]. Importantly, none of the candidate steroidal compounds demonstrated the ability to cross the blood–brain barrier, a desirable trait for minimizing central nervous system side effects in non-CNS-targeted therapies [39,40]. Additionally, all compounds exhibited moderate synthetic accessibility scores (ranging from 4.31 to 4.52), indicating that they are within a practical range for chemical synthesis and scale-up.

Among the candidates, E-254 and E-265 emerged as the most promising prototypes, combining high predicted absorption, absence of major toxicological alerts, log *p* values near the optimal threshold of 5, and a favorable balance between solubility and synthetic feasibility. E-264 remains a viable lead, provided its hepatotoxicity can be mitigated through rational design. Meanwhile, E-268 offers a structurally interesting alternative with good acute safety indicators, though its long-term tolerability warrants further investigation.

When compared with the reference drugs, RM-581, exemestane, and formestane, the newly designed steroid compounds show several clear advantages. First, all candidates have molecular weights under 500 g/mol, which is beneficial for passive membrane permeability. In contrast, RM-581 exceeds this threshold, which may limit its absorption. Among the new compounds, E-254, E-265, and E-264 stand out for maintaining an ideal lipophilicity, with log *p* values close to 5. This balance supports good membrane permeability without compromising solubility. On the other hand, exemestane and formestane are more hydrophilic (Log *p* around 3.1–3.5), and although RM-581 has a Log *p* of 4.78, its high molecular weight and flexible structure reduce its overall drug-likeness.

From a safety perspective, only E-264 and RM-581 raised hepatotoxicity concerns. Interestingly, the clinical inhibitors did not trigger this alert, although formestane showed the weakest toxicological profile overall, being classified in the lowest safety category (class 6). In terms of synthetic feasibility, the new steroid candidates also performed well, with synthetic accessibility scores averaging around 4.4, lower (and thus more favorable) than exemestane (5.03) and especially RM-581 (5.92), suggesting they are easier and more cost-effective to produce. Finally, none of the new steroid compounds are predicted to cross the blood–brain barrier, which is a desirable trait for drugs not intended to act on the central nervous system. This reduces the risk of neurological side effects, a notable advantage over exemestane and formestane, which do have the potential to enter the brain.

Following the confirmation that all designed compounds met acceptable ADME-Tox criteria, we proceeded to investigate their molecular interactions with the selected protein targets. This analysis was conducted using PyMOL and Discovery Studio Visualizer, which allowed for detailed visualization of the binding modes and interaction profiles. The resulting 2D interaction diagrams, presented in Table 8, Table 9 and Table 10, illustrate the key contacts formed between each compound and its respective target, offering valuable insights into the structural features that contribute to binding affinity and specificity.

Docking simulations against the progesterone receptor ligand-binding domain (PDB ID: 2W8Y, Table 8) revealed that all seven designed steroidal compounds consistently occupied the same hydrophobic pocket. This cavity is primarily defined by residues such as Leu715, Leu718, Leu721, Leu763, Val760, Met756, Met759, Phe778, and Met801/Met909. Within this site, the ligands formed a stable network of van der Waals, alkyl, and π–alkyl interactions, which contributed significantly to binding affinity. Each compound also exhibited unique polar and π-stacking interactions superimposed on this shared hydrophobic framework. For instance, E-253 formed π–sulfur and π–alkyl contacts without steric clashes. E-254 maintained a similar interaction pattern but introduced dual π–sulfur interactions and a hydrogen bond with Arg766, slightly improving its binding energy. E-255 added hydrogen bonds with Gln725 and Arg766, along with a π–sulfur contact, though a minor clash at Phe778 was observed. E-261 preserved the hydrophobic core and established hydrogen bonds with Arg766 and Gln725, plus a π–sulfur interaction, despite mild steric interference. E-264 demonstrated enhanced aromatic interactions, forming dual π–π stacking contacts with Phe778 and Tyr890, a halogen bond with Met909, and a hydrogen bond with Arg766—though accompanied by steric hindrance near Gln725. E-265 retained the core interaction profile and added hydrogen bonds and a π–sulfur contact, resulting in an intermediate binding profile. Lastly, E-268 relied on halogen bonding and π–π interactions to maintain affinity, despite lacking conventional hydrogen bonds.

On the other hand, Table 9 presents the 2D interaction for the seven steroidal ligands with the highest predicted affinity toward the HER2 tyrosine kinase domain (PDB ID: 7JXH). In all complexes, the hydrophobic tetracyclic core of the ligands consistently fits into a conserved pocket defined by Leu785, Leu800, and Met801, forming a stable network of van der Waals, alkyl, and π–alkyl interactions that dominate the binding energy landscape. Estero-253 reinforces this hydrophobic anchoring with a T-shaped π–π stacking interaction with Phe864 and a conventional hydrogen bond to the carboxylate of Asp863, effectively stabilizing its terminal orientation. Estero-254 maintains the same hydrophobic profile but introduces an additional π–anion interaction with Asp863 and a C–H···O hydrogen bond, which likely accounts for its slightly improved binding energy. Estero-255 combines the core hydrophobic contact with a weak hydrogen bond to the ε-ammonium group of Lys753 and shows no steric clashes, consistent with its low fluctuation during molecular dynamics simulations.

In the case of Estero-264, the extended aromatic substituent enables dual π–π stacking with Phe864 and Tyr835. A halogen bond between its fluorine atom and Phe864, along with electrostatic interaction with Lys753, further enhances its binding affinity. Finally, Estero-265 forms two classical hydrogen bonds with Glu770 and Asp863 and establishes a halogen contact with Tyr835. However, a donor–donor clash with Asp863 may slightly reduce its binding efficiency.

Finally, Table 10 shown the Docking simulations of the seven designed steroidal compounds into the ligand-binding domain of estrogen receptor-α (ER-α, PDB ID: 6VJD). Table 10 reveals a consistent binding mode across the series. In all cases, the tetracyclic steroid core was stably positioned within a hydrophobic pocket defined by residues Leu346, Leu349, Leu384, Leu428, Leu525, Met343, Met388, Met421, Ile424, and Pro535. This region formed a dense network of van der Waals, alkyl, and π–alkyl interactions, which dominated the binding energy profile for each complex. E-253 complemented this hydrophobic anchoring with a π–anion interaction involving Asp351, a π–sulfur contact with Met388, and a C–H···O hydrogen bond to His524. E-254 preserved the same core interactions and added a T-shaped π–π stacking interaction with Phe404 and a π–sulfur contact with Met421, resulting in a modest increase in binding affinity. E-255 introduced two classical hydrogen bonds with Arg394 and His524, along with a π–σ interaction with Met388. Although a minor acceptor–acceptor clash with Leu525 was observed, it did not disrupt the ligand’s position within the pocket. E-264, featuring an extended aromatic substituent, formed three simultaneous π–π stacking interactions with Phe404, Tyr526, and Trp383, and established a hydrogen bond with Arg394. These interactions compensated for its larger molecular size. E-261 maintained the core hydrophobic interactions and added a π–σ contact with Met388 and a hydrogen bond to His524, though minor steric clashes with Leu384 and Leu428 were detected. E-265 formed a π–sulfur interaction with Met343 and hydrogen bonds with Arg394 and His524, yielding an interaction profile intermediate in complexity between E-255 and E-253. Lastly, E-268 relied on a fluorine-centered halogen bond with Leu525, a π–σ interaction with Met388, and π–π stacking with Trp383 to maintain strong binding affinity, despite lacking classical hydrogen bonds.

Once molecular docking identifies potential binding poses between a ligand and its target, it becomes essential to evaluate the stability and realism of these complexes over time. To achieve this, molecular dynamics (MD) simulations are employed. Unlike docking, which provides a static snapshot, MD allows us to observe how the protein–ligand complex behaves in a dynamic, solvated environment—closer to physiological conditions. This step helps confirm whether the predicted interactions are stable, whether the ligand remains in the binding pocket, and how the complex responds to thermal fluctuations, thus offering a more reliable assessment of binding affinity and structural compatibility.

After confirming that all designed compounds met acceptable ADME-Tox criteria, we analyzed their molecular interactions with the selected protein targets. This study was performed using PyMOL and Discovery Studio Visualizer, tools that allowed detailed visualization of binding modes and interaction profiles. Comprehensive tables summarizing every interaction type for each ligand–protein pair (Table 8, Table 9 and Table 10) were prepared, and the full set of 2D interaction images is provided in the Supplementary Material (Figures S1–S3).

**Figure 5:**
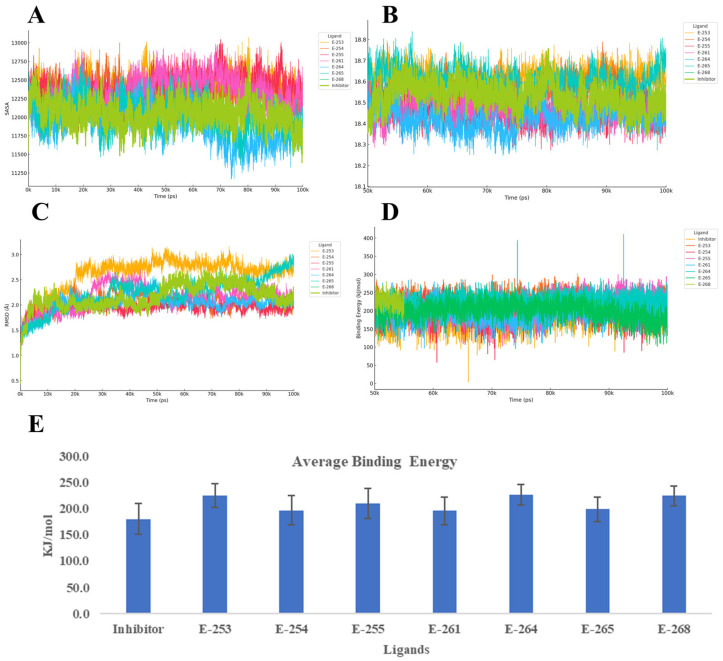
Structural and energetic evolution of the 2W8Y complex over 100 ns of molecular dynamics simulations with seven steroid derivatives and a reference inhibitor: panel (**A**) displays the solvent-accessible surface area (SASA), panel (**B**) the variation in radius of gyration, panel (**C**) the backbone RMSD, panel (**D**) the binding-energy time courses calculated via MMPBSA, and panel (**E**) the mean ± SD of this energy; together, these curves and bars enable comparison of the conformational stability and relative affinity of E-253, E-254, E-255, E-261, E-264, E-265, E-268, and the inhibitor toward the target protein.

To evaluate the dynamic stability and binding behavior of the seven designed steroidal analogs with the progesterone receptor (PR, PDB ID: 2W8Y), 100 ns MD simulations were performed using YASARA, with the co-crystallized inhibitor serving as a reference. The analysis focused on four key parameters: Cα RMSD, radius of gyration (Rg), secondary structure evolution, and YASARA’s Binding Energy score proprietary metric, where higher values indicate stronger interactions (Figure 5).

Across the simulations, Estero-264 and Estero-255 formed the most stable complexes, with RMSD values around 1.5 Å and slightly more compact receptor conformations (Rg ≈ 18.46–18.47 Å) compared with the reference. These were followed by Estero-254 and Estero-261, which showed similar stability to the inhibitor (RMSD ≈ 1.6–1.7 Å). In contrast, Estero-253 exceeded 2 Å RMSD, suggesting conformational adaptation of the receptor to accommodate the ligand. Secondary structure analysis confirmed that the top-performing compounds preserved α-helical content, while Estero-265 and Estero-268 induced modest coil formation and, in the latter case, a net 3% helix loss. All candidates outperformed the inhibitor in terms of Binding Energy, with Estero-264 (226.8 ± 19.8 kcal/mol) and Estero-253 (226.2 ± 22.6 kcal/mol) showing the highest scores. Estero-268 and Estero-255 followed closely, while Estero-265, Estero-254, and Estero-261 still exceeded the reference by 15–18 kcal/mol within the method’s uncertainty but are indicative of improved affinity.

Taken together, the combination of low RMSD, reduced Rg, and high Binding Energy identifies Estero-264 and Estero-255 as the most robust and tightly bound complexes. Estero-254, Estero-261, and Estero-265 also show a favorable balance between structural stability and binding strength. Notably, Estero-265 exhibited the narrowest energy distribution, suggesting a consistently stable interaction network throughout the simulation. Even additional experiments are necessary, these results suggest that the designed steroids likely act as competitive inhibitors, stabilizing the receptor in a compact, low-energy conformation through persistent hydrophobic and polar interactions. The reduced flexibility (low RMSD), compactness (low Rg), and enhanced binding energy collectively support their potential to effectively block natural ligand access to the receptor’s active site [41].

**Figure 6:**
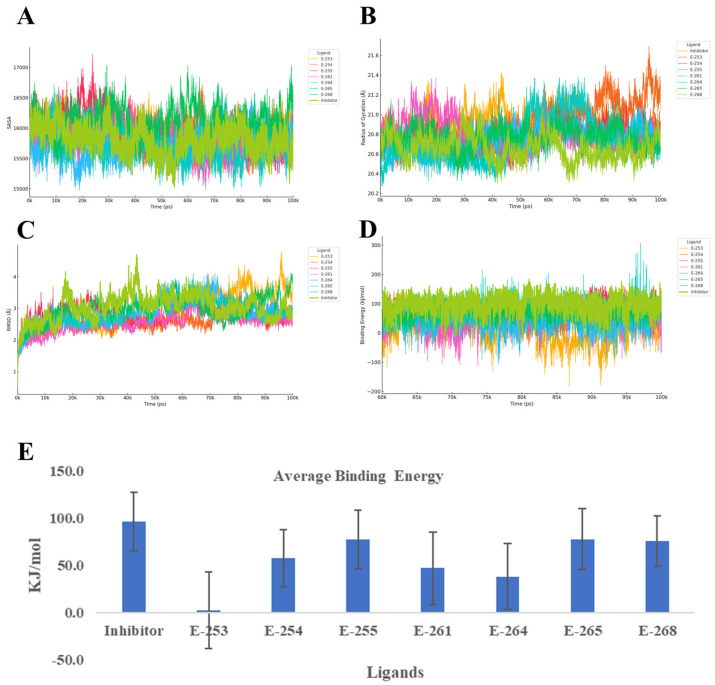
Structural and energetic evolution of the 7JXH complex over 100 ns of molecular dynamics simulations with seven steroid derivatives and a reference inhibitor: panel (**A**) displays the solvent-accessible surface area (SASA), panel (**B**) the variation in radius of gyration, panel (**C**) the backbone RMSD, panel (**D**) the time courses of binding energy calculated via MMPBSA, and panel (**E**) the mean ± SD of this energy; together, these curves and bars enable comparison of the conformational stability and relative affinity of E-253, E-254, E-255, E-261, E-264, E-265, E-268, and the inhibitor toward the target protein.

To better understand how the designed steroidal compounds interact with the HER2 tyrosine kinase domain (PDB ID: 7JXH), we ran 100-nanosecond molecular dynamics simulations using YASARA, using the co-crystallized inhibitor as a benchmark. We focused on four key indicators of complex behavior: Cα RMSD, radius of gyration (Rg), secondary structure changes, and YASARA’s Binding Energy score (Figure 6). Throughout the simulations, all compounds maintained the structural integrity of the HER2 domain. Estero-261 and Estero-254 formed the most stable complexes, with RMSD values around 2.1–2.2 Å—more stable than the reference inhibitor (2.65 Å). The Rg values remained consistent (~20.8 Å), indicating no significant unfolding or collapse of the protein. Most compounds preserved the receptor’s secondary structure, with only Estero-268 (+4.8%) and Estero-264 (+2.7%) showing modest increases in coil content, but without signs of destabilization.

In terms of binding strength, Estero-255, Estero-265, and Estero-268 showed the highest affinity, with Binding Energy scores between 75–78 kcal/mol—approaching the reference inhibitor’s 96.4 ± 31.3 kcal/mol. Interestingly, Estero-254 and Estero-261, despite their excellent structural stability, showed moderate binding energies (47–57 kcal/mol), suggesting that while they fit well geometrically, they may benefit from additional polar interactions to improve electrostatic complementarity. On the other hand, Estero-264 and especially Estero-253 (~2 kcal/mol) showed weak binding, likely due to poor contact formation or excessive flexibility.

When considering both stability and affinity, Estero-255 and Estero-265 stand out as the most promising HER2 inhibitors. They combine strong binding with structural stability and minimal disruption to the protein’s secondary structure. Estero-254 and Estero-261 offer rigid scaffolds that could be optimized further, while Estero-264 and Estero-268 may require structural refinement to improve their interaction profiles. Estero-253, due to its very low binding energy, appears unsuitable for this target. Finally, the binding profiles suggest that Estero-255 and Estero-265 likely act as ATP-competitive inhibitors. Their ability to maintain low RMSD and stable Rg values, along with high binding energy, indicates that they effectively occupy the ATP-binding cleft and stabilize the kinase in an inactive conformation. This mechanism aligns with the established mode of action for HER2-targeted tyrosine kinase inhibitors, which block ATP access and prevent downstream phosphorylation events critical for cancer cell proliferation [42].

**Figure 7:**
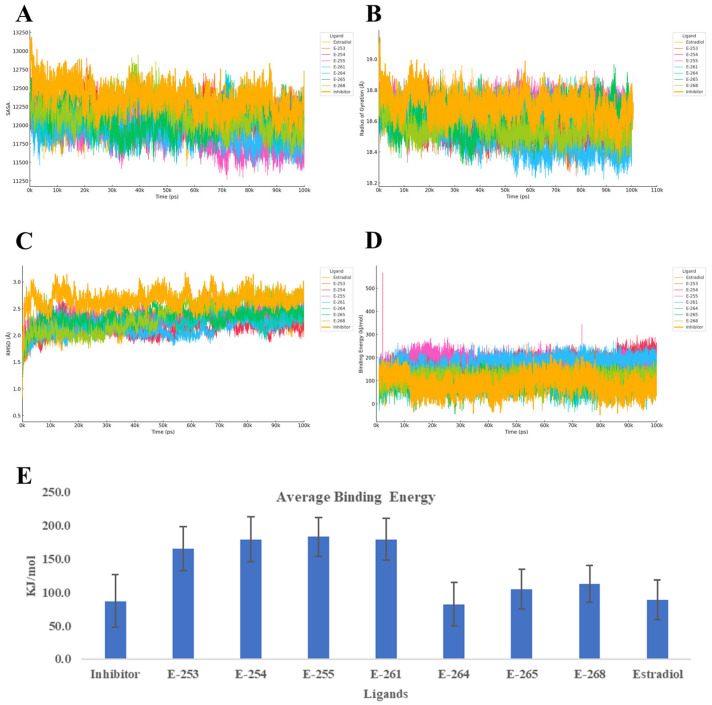
Structural and energetic evolution of the 6VJD complex over 100 ns of molecular dynamics simulations with steroid ligands: panel (**A**) displays the solvent-accessible surface area (SASA), panel (**B**) the variation in radius of gyration, panel (**C**) the backbone RMSD, panel (**D**) the time courses of binding energy, and panel (**E**) the mean ± SD of this energy; together, these curves and bars enable comparison of the conformational stability and relative affinity of derivatives E-253, E-254, E-255, E-261, E-264, E-265, E-268, estradiol, and the reference inhibitor toward the target protein.

Finally, the 100-nanosecond molecular dynamics (MD) simulations of the estrogen receptor alpha ligand-binding domain (ER-α, PDB ID: 6VJD) in complex with the designed steroid analogs demonstrate favorable structural and energetic profiles (Figure 7). All compounds preserved the receptor’s global fold, while several analogs exhibited superior binding characteristics compared with both estradiol and the co-crystallized inhibitor.

Among the tested ligands, Estero-261 and Estero-254 were particularly effective. These analogs significantly reduced the Cα RMSD (~1.5 Å), indicating enhanced structural stability, and slightly decreased the radius of gyration (Rg), suggesting a more compact and potentially less flexible receptor conformation. These structural effects were accompanied by a marked increase in binding energy, nearly doubling the values observed for estradiol and the reference inhibitor. Such stabilization of the ligand-binding domain (LBD) is known to interfere with the dynamic positioning of helix 12 (H12), a critical element for coactivator recruitment and transcriptional activation [43]. Estero-255 also demonstrated a substantial gain in binding energy, with only a marginal increase in backbone flexibility, while Estero-253 presented a balanced profile—moderate RMSD and Rg values coupled with high affinity—suggesting that further rigidification of its steroidal scaffold could enhance its inhibitory potential. Conversely, Estero-264 did not exhibit significant improvements in any of the evaluated parameters. Its RMSD and Rg values were more variable, and its binding energy remained comparable to the controls, indicating limited potential without further structural optimization.

Overall, these findings support a competitive inhibition mechanism, wherein the top-performing analogs occupy the ligand-binding pocket with higher affinity than estradiol, thereby preventing receptor activation. The observed reduction in RMSD and compaction in Rg suggest that these ligands stabilize the receptor in an inactive conformation, potentially obstructing coactivator binding [44]. This interpretation is further supported by the enhanced binding energies, which are predictive of stronger ligand–receptor interactions and inhibitory potency [45,46].

### 2.7. Multitarget Molecular Dynamics Analysis of Steroid Analogs in Triple-Positive Breast Cancer

The 100-nanosecond molecular dynamics simulations conducted across the three principal targets in triple-positive breast cancer—progesterone receptor (PR, PDB ID: 2W8Y), HER2 tyrosine kinase (PDB ID: 7JXH), and estrogen receptor alpha (ER-α, PDB ID: 6VJD) enabled a comprehensive evaluation of seven designed steroid analogs (Estero-253, -254, -255, -261, -264, -265, and -268). This unified approach allowed for the identification of ligands with consistent performance across multiple receptor classes, a key criterion in the development of multitarget therapeutics.

Analysis of structural stability parameters, including root mean square deviation (RMSD), radius of gyration (Rg), and secondary structure retention, revealed that Estero-254 and Estero-261 consistently exhibited the lowest RMSD values and most compact Rg profiles across all three targets. These findings suggest a pre-organized chemical scaffold that minimizes the conformational rearrangement required upon binding, thereby enhancing complex stability. Estero-255 also demonstrated favorable rigidity in PR and ER-α, with acceptable RMSD values in HER2, while Estero-264 maintained structural integrity only in PR. In contrast, Estero-268 and Estero-253 induced significant helical perturbations and exhibited high flexibility, compromising their overall stability.

Energetic profiling further reinforced this hierarchy. Estero-261 emerged as the only compound to achieve top-tier binding energy scores across all three targets, doubling the reference inhibitor’s score at ER-α, outperforming it at PR, and approaching it within ~20 kcal/mol at HER2. Estero-254 mirrored this profile with negligible deviation. Estero-255 also reached peak affinity at PR and ER-α, and although its binding energy at HER2 (~78 kcal/mol) was more modest, it still surpassed the reference compound. Estero-265 showed strong affinity at HER2 but lacked sufficient electrostatic interaction at the nuclear receptors, while Estero-253, -264, and -268 were deprioritized due to weak binding or instability at two or more targets.

Detailed RMSD analysis confirmed that Estero-261 and Estero-265 provided the most stable complexes in PR (1.50 Å and 1.54 Å, respectively), while Estero-261 and Estero-253 were most stable in ER-α and HER2, with RMSD values below 1.80 Å. Rg analysis indicated that Estero-255 and Estero-261 induced the most compact conformations in the nuclear receptors, suggesting enhanced conformational stability. Total energy calculations supported these findings: Estero-255 and Estero-253 showed the most negative values in PR, Estero-265 and Estero-264 in ER-α, and Estero-268 and Estero-255 in HER2. Electrostatic (Coulomb) and van der Waals interaction analyses highlighted Estero-255 as a strong binder in PR and ER-α, while Estero-253 also demonstrated favorable short-range interactions across all targets. Hydrogen-bonding analysis, a critical determinant of complex specificity and stability, revealed that Estero-255 and Estero-253 formed the highest number of internal hydrogen bonds in PR, reinforcing their structural integrity. Conversely, Estero-265 and Estero-268 showed greater solvent interaction in ER-α and HER2, which may contribute to their solubility and bioavailability in physiological environments.

These results highlight the strong potential of our steroid-based compounds to act as multitarget inhibitors in triple-positive breast cancer. What stands out is how consistently Estero-255 and Estero-265 perform across three very different types of receptors, two nuclear hormone receptors (PR and ER-α) and a membrane-bound tyrosine kinase (HER2). This kind of versatility suggests that these molecules are not only structurally stable but also adaptable enough to engage with diverse binding environments without losing affinity or precision. That’s a rare and valuable trait in drug design.

Estero-255 shows a well-rounded profile, combining strong binding, compact structure, and stable interactions across all targets. Estero-265, while slightly more selective, shows excellent performance in ER-α, which is especially relevant for hormone-driven cancers. These findings support the idea that a multitarget approach, rather than focusing on a single receptor, could offer a more effective strategy for tackling the complexity of breast cancer. With further optimization and experimental validation, these compounds could serve as promising leads for the next generation of targeted therapies [47,48].

## 3. Discussion

Estrogen receptors (ER) and progesterone receptors (PR) belong to the steroid nuclear receptor superfamily and share a highly conserved modular architecture that yields nearly parallel activation and regulatory mechanisms [49]. Both proteins comprise six functional domains (A–F) arranged from the N- to the C-terminus; the A/B domain hosts ligand-independent activation function 1 (AF-1), whose presence or absence, as in the ERα46 isoform, introduces selectivity nuances that multitarget ligands can exploit. The C domain (DBD), almost identical in ER and PR, contains the zinc fingers responsible for sequence-specific DNA binding and receptor dimerization; therefore, a compound that stabilizes or disrupts this region in one receptor is very likely to influence the other. The hinge region (D) provides the nuclear-localization signal and binding sites for corepressors, whereas the ligand-binding domain (LBD, E domain), with ≈60% sequence homology, houses both the ligand pocket and ligand-dependent activation function 2 (AF-2) [49,50,51,52,53,54,55,56,57,58]. Chemical modifications directed at key residues in this cavity can preserve cross-affinity and simultaneously modulate the transcriptional activity of both receptors. In addition, the ability of ER and PR to form homo- or heterodimers, together with the conserved “L-shaped boot” spatial arrangement of their DBD–LBD tandem, suggests that conformational changes induced by a ligand in one receptor will likely be mirrored in the other, reinforcing the multitarget rationale [53].

By contrast, approved therapies against the tyrosine kinase receptor HER2—monoclonal antibodies (trastuzumab, pertuzumab), tyrosine kinase inhibitors (lapatinib, neratinib), and antibody–drug conjugates (T-DM1, trastuzumab deruxtecan)—focus exclusively on blocking HER2 signaling or eliminating cells that overexpress it. To date, no clinical candidates directly and simultaneously target HER2 together with members of the nuclear receptor superfamily. Nevertheless, evidence of functional crosstalk between HER2 and several nuclear receptors, particularly the androgen receptor, strengthens the hypothesis that a molecule capable of concertedly modulating HER2, ER, and PR could overcome resistance, reduce adverse effects, and enhance therapeutic efficacy in hormone-dependent tumors [59].

This study demonstrates the value of an integrated computational pipeline, combining 3D-QSAR modeling, molecular docking, and molecular dynamics (MD) simulations, for identifying steroidal scaffolds with multitarget potential against the three key drivers of triple-positive breast cancer: PR, ER-α, and HER2. Starting from a library of 271 DFT-optimized derivatives, we narrowed the field to seven lead compounds with strong predicted potency (pIC_50_ > 7.0) and favorable binding energies across at least two targets.

The 3D-QSAR model, built on eight QuBiLS-MIDAS descriptors, showed excellent predictive performance (R = 0.93; Q^2^ = 0.86), surpassing OECD thresholds for reliability. Descriptor analysis revealed that anticancer activity correlates positively with molecular polarizability and hydrogen-bond donor count, and negatively with excessive electronegativity and softness, guiding the rational inclusion of aromatic and halogen substituents to fine-tune electronic properties.

Docking results prioritize seven analogs, Estero-253, -254, -255, -261, -264, -265, and −268, based on their ability to bind PR, ER-α, and HER2 with ΔG ≤ −9 kcal/mol. Estero-255 and Estero-261 matched or exceeded the affinities of clinical agents like Exemestane and Formestane, establishing them as internal benchmarks.

MD simulations confirmed the structural robustness of these ligands. Estero-261 consistently showed the lowest RMSD values (1.50–1.58 Å) in PR and ER-α, while Estero-255 and Estero-253 also maintained stable conformations (RMSD < 1.9 Å). These results are supported by compact radii of gyration (~18.3 Å) and dense hydrogen-bond networks, particularly in Estero-255. Binding energy traces stabilized early, indicating no late-stage unbinding events.

Interaction mapping revealed conserved hydrophobic contacts with Leu, Met, and Phe residues, while π–π and π–σ stacking interactions (especially in Estero-254 and -261) enhanced binding to Tyr890 (PR) and Phe864 (HER2). Halogenated analogs like Estero-268 formed halogen–π interactions that likely contribute to their PR selectivity. Estero-255, enriched in hydrogen-bond donors, formed classical H-bonds with Arg766 (PR) and His524 (ER-α), anchoring the steroid core.

Compared with clinical inhibitors, the new ligands offer superior predicted potency (pIC_50_ up to 9.2 vs. 5.7) and verified multiactivity. This multitarget profile is particularly relevant in triple-positive breast cancer, where PR, ER-α, and HER2 converge on the PI3K/AKT/mTOR (PAM) pathway, a central axis of tumor growth and endocrine resistance. HER2 activates PAM via direct PI3K recruitment; ERs contribute through both genomic and non-genomic mechanisms; and PR, primarily through PGRMC1, reinforces the same cascade. These pathways are interlinked, forming a feedback loop that sustains proliferation and survival even under targeted therapy.

Simultaneous inhibition of PR, ER-α, and HER2 disrupts this feedback, collapsing the PAM axis. AKT inactivation reactivates pro-apoptotic proteins and suppresses survival signals; mTORC1 blockade halts biosynthesis and induces energetic stress; and cell-cycle arrest is triggered via p21/p27 restoration and cyclin D1 degradation. This reprogramming not only promotes apoptosis but also sensitizes tumor cells to chemotherapy and endocrine agents, addressing a major resistance mechanism in this subtype.

Beyond binding affinity and structural stability, the drug-likeness and pharmacokinetic profiles of the lead compounds were also evaluated to assess their translational potential. All top-performing analogs, particularly Estero-261, Estero-254, and Estero-255, exhibited favorable physicochemical properties, including moderate lipophilicity (log *p* < 5), acceptable molecular weights (<500 Da), and low polar surface areas, aligning with Lipinski’s Rule of Five. In silico ADMET predictions revealed no major red flags for mutagenicity, hepatotoxicity, or cardiotoxicity, and synthetic accessibility scores (SA ≈ 4.3–4.5) suggest that these compounds are amenable to practical synthesis and scale-up. These attributes collectively support their candidacy for further preclinical development and formulation studies.

While the computational results are promising, several limitations must be acknowledged. First, the simulations were conducted under idealized conditions and do not account for the full complexity of the tumor microenvironment, including protein–protein interactions, metabolic degradation, or immune modulation. Second, although the multitarget activity of the compounds is well supported in silico, experimental validation—such as receptor binding assays, cell-based functional studies, and in vivo efficacy models—will be essential to confirm their therapeutic potential. Future work should also explore structure–activity relationships (SAR) around the Estero-255 and Estero-261 scaffolds to optimize selectivity, solubility, and metabolic stability. Additionally, combination studies with existing endocrine or HER2-targeted therapies could reveal synergistic effects and inform rational co-treatment strategies.

From a therapeutic perspective, a single compound capable of targeting all three receptors offers a dual advantage: it blocks hormone-driven proliferation and prevents HER2-mediated escape. Within this framework, Estero-261 and Estero-254 emerge as the most promising leads, combining strong binding, structural stability, and favorable safety profiles. Estero-255 and Estero-265, with their complementary energetic and structural features, expand the chemical space for future optimization and resistance management.

## 4. Materials and Methods

### 4.1. Development of Datasets and Calculation of Molecular Descriptors

To assemble a consistent and reliable dataset for this study, we conducted a thorough search for steroidal compounds with reported anticancer activity against breast cancer. This process involved mining several reputable sources, including peer-reviewed literature and specialized chemical and pharmacological databases such as PubChem, ChEMBL, and DrugBank. The search strategy was guided by keywords like “steroids”, “steroidal compounds”, and “breast cancer”. A key inclusion criterion was that every selected compound had been assessed for anticancer activity using the MTT assay—which relies on the reduction of 3-(4,5-dimethylthiazol-2-yl)-2,5-diphenyltetrazolium bromide—over 24 h in triple-positive breast cancer cell lines, with its IC_50_ reported in µM. In addition, the experiments had to follow identical or highly comparable protocols. This requirement was critical to ensure that the biological activity data were directly comparable across the dataset. Compounds lacking clear methodological consistency or tested under differing conditions were excluded to preserve the integrity and predictive reliability of the computational models developed in this study.

The chemical structures of the selected steroid compounds were obtained in digital format and subsequently pre-optimized using Avogadro 1 (GitHub 2022) and Chem3D^®^ Professional software 17.1. (PerkinElmer, Waltham, MA, USA) [60,61]. Then, the conformation with the minimum energy state was found using GaussView software, version 6, at the DFT level using the combination of correlation exchange WB97XD functional and the basis set LanL2DZ [60,62]. Additionally, various molecular descriptors were calculated, including topological, thermodynamic, and electronic, by means of Chem3D, Gaussian 16, and QuBiLS-MIDAS, respectively [63,64,65].

### 4.2. Target Selection and Ligand-Inhibitors

To investigate the molecular interactions of the designed steroidal compounds, we selected three key protein targets that play central roles in the development and progression of hormone-dependent breast cancer. These targets were retrieved from the RCSB Protein Data Bank (PDB) and include Progesterone receptor (PR)—PDB ID: 2W8Y; Human epidermal growth factor receptor 2 (HER2)—PDB ID: 7JXH, and Estrogen receptor alpha ligand-binding domain (ERα-LBD)—PDB ID: 6VJD. These proteins were chosen based on their well-established involvement in breast cancer biology and their relevance as therapeutic targets for steroidal agents.

Estrogen and progesterone receptors are nuclear transcription factors that, when activated by their respective hormones, regulate gene expression patterns that promote cell proliferation and survival. In many breast cancers, especially those classified as hormone receptor-positive, these pathways are overactive, making them prime targets for therapeutic intervention. Steroidal compounds, due to their structural similarity to natural hormones, are particularly well suited to interact with these receptors—either to block their activity or modulate their function [66,67]. On the other hand, HER2 is a membrane-bound tyrosine kinase receptor that, when overexpressed, drives aggressive tumor growth and poor prognosis. Although HER2-positive breast cancers are often treated with monoclonal antibodies or kinase inhibitors, there is growing interest in small molecules—including steroidal derivatives—that can bind to and modulate HER2 activity through alternative mechanisms [68]. Finally, the estrogen receptor alpha (ERα) is perhaps the most critical target in hormone-dependent breast cancer. It mediates the effects of estrogen on gene expression and cell proliferation. ERα-positive tumors account for most breast cancer cases, and therapies that block or degrade this receptor—such as selective estrogen receptor modulators (SERMs) and degraders (SERDs)—are standard treatments. Understanding how new compounds interact with ERα is essential for developing next-generation endocrine therapies [69].

By focusing on these three targets, our goal is to capture a broad yet biologically coherent spectrum of hormone-related pathways in breast cancer. This approach not only enhances the relevance of our findings but also increases the likelihood of identifying compounds with multitarget potential—an increasingly valuable trait in modern oncology drug development.

### 4.3. QSAR Analysis

The dataset was partitioned into a training set (70%) and a test set (30%) using random sampling. Cross-validation techniques, including LOOCV, were subsequently applied to the training set to evaluate the internal robustness and predictive reliability of the model. The QSAR-3D model was developed using IBM^®^ SPSS^®^ Statistics version 17.0.0 for Windows. Descriptors with autocorrelation were discarded, and relevant descriptors capturing the chemical and structural information of the steroid compounds were selected [65]. QSAR modeling methods, such as multiple linear regression and principal component analysis (PCA), were applied. The robustness and predictability of the QSAR model were evaluated using metrics such as R^2^, Q^2^, RMSE, and QMRF, tested through the leave-one-out cross-validation method (LOOCV) [65,70,71,72].

### 4.4. Design of New Steroid-Derivative Compounds

To design new steroidal derivatives with potential anticancer activity, we followed a step-by-step computational approach that combined QSAR modeling, molecular docking, and molecular dynamics simulations. We began by using a validated QSAR model to pinpoint the molecular features most strongly linked to high anticancer activity. These insights guided the thoughtful modification of the most promising compounds in our dataset, aiming to enhance their biological potential. For the design of the new ligands, various substituents were introduced into the steroidal scaffold, including alkyl (–CH_3_), carbonyl (C=O), nitrile (C≡N), halogen (F, Cl, Br, I), nitro (–NO_2_), sulfoxide (–SO), and sulfone (–SO_2_) groups, among others.

Once modified, the new molecules were re-evaluated by recalculating their molecular descriptors using the same combination of DFT level. Only those that showed equal or better predicted activity compared with the original compounds were selected for further analysis. These candidates were then tested for their ability to bind to the target protein using molecular docking techniques. The compounds with the strongest predicted binding affinities were further screened using ADMET and toxicity prediction tools to assess their pharmacokinetic behavior and safety.

Finally, the top-performing candidates underwent molecular dynamics simulations. This step allowed us to observe how the drug–target complexes behaved over time, offering valuable insights into their stability and interactions at the atomic level. Altogether, this integrated strategy provided a rational and efficient pathway for identifying promising steroidal compounds for further development against hormone-dependent breast cancer.

### 4.5. Docking and Molecular Dynamics

Only high-resolution, mutation-free protein structures co-crystallized with their natural ligand or an antagonist inhibitor were downloaded. The X-ray crystal structures, complexed with the natural ligand or inhibitor, were obtained from the RCSB Protein Data Bank (http://www.rcsb.org/, accessed on 20 February 2024) in .pdb format. Each three-dimensional structure was first modeled on the AlphaFold platform (https://alphafold.ebi.ac.uk/, accessed on 25 February 2024) and subsequently refined in PyMOL 2.1 (Schrödinger, New York, NY, USA). During this preparation, a single monomer with its corresponding ligand was retained, all water molecules and non-standard residues were removed, and hydrogen atoms were added to optimize hydrogen bonding. For each protein–ligand complex, the docking site was defined using the position of the endogenous ligand or substrate as a reference, ensuring that the study focused on the physiologically relevant region (orthosteric pocket). In the progesterone receptor (PR; PDB ID 2W8Y), the selected pocket exhibited a volume of 583 Å^3^ with a geometric center at (−2, −7, 25 Å). In the ligand-binding domain of estrogen receptor-α (ERα-LBD; PDB ID 6VJD), a considerably larger cavity of 2176 Å^3^ was identified, centered at (29, 11, 33 Å). Finally, in human epidermal growth factor receptor 2 (HER2; PDB ID 7JXH), the calculated pocket volume was 306 Å^3^ with a center at (67, 13, 83 Å). The CB-Dock2 platform ©YANG CAO LAB (https://cadd.labshare.cn/cb-dock2/index.php, accessed on 15 February 2024) was used to predict the conformation and binding affinity of both the dataset and the designed compounds with the receptor proteins [72,73]. In the docking protocol, nine poses were generated using AutoDock Vina (Version 2.0, January 2004) iterative local-search algorithm. Each pose was further refined with the Broyden–Fletcher–Goldfarb–Shanno (BFGS) optimizer. A moderate exhaustiveness level of 8 was selected. AutoDock Vina estimated the binding free energy (ΔG) with its Vina scoring function, which combines van der Waals, repulsion, electrostatic, and other energetic contributions.

Molecular dynamics (MD) simulations were performed using YASARA with the AMBER14 force field. This macro applies AMBER14/ff14SB to the protein and GAFF-2 (via AutoSMILES) to the ligands, ensuring energetic consistency between both components during the molecular dynamics simulation. All ligands were parameterized using YASARA’s automatic md_run.mcr workflow, fully compatible with AMBER14/ff14SB. Each ligand was first converted to a canonical SMILES string with AutoSMILES, which adjusts protonation states and bond orders to pH 7.4. Atom types and bonded/van-der-Waals parameters were then assigned with GAFF-2, while partial charges were computed via AM1-BCC, reproducing RESP/HF-6-31G* electrostatics (r.m.s.d. ≈ 0.04 e) at minimal computational cost. GAFF-2 parameters and AM1-BCC charges were combined into a topology file that YASARA merged with the protein–solvent system under a single AMBER14 scheme, automatically checking for missing terms. This procedure ensures an energetically consistent protein–ligand description for all molecular dynamics simulations. The system was solvated in explicit water (TIP3P) and neutralized with Na+ and Cl- ions at a 0.9% NaCl concentration. Periodic boundary conditions were applied, with temperature and pressure set to 300 K and 1 atm using the Berendsen thermostat and barostat. The Leap-Frog algorithm integrated equations of motion with a 2 fs timestep. After 500 ps of equilibration, a 100 ns production run was carried out, saving snapshots every 10 ps. Finally, the trajectory analysis was performed with the md_analyze.mcr macro, generating RMSD, energy, and hydrogen bond plots. Ligand binding energy was evaluated using the md_analyzebindenergy.mcr macro, providing average binding energy values over the simulation time [74,75,76,77]. Results were exported for further statistical analysis.

## 5. Conclusions

This study demonstrates that a step-wise computational workflow combining a robust eight-descriptor 3D-QSAR model (R^2^ = 0.86), docking-based virtual screening, and 100 ns molecular dynamics validation can rapidly identify multitarget steroidal leads for hormone-dependent breast cancer. From 271 in-silico-optimized derivatives, seven candidates (Estero-253, -254, -255, -261, -264, -265, and -268) surpassed the affinity benchmark (ΔG ≤ −9 kcal mol^−1^ for at least two targets) and exhibited predicted potencies of pIC_50_ ≥ 7.2. MD trajectories confirmed that Estero-255 and Estero-261 establish exceptionally stable complexes with the progesterone and estrogen receptors, while Estero-264 secures the tightest HER2 binding through additional π–π and halogen–π interactions.

Crucially, the ADME/Tox analysis refines these rankings: five candidates—Estero-254, -255, -264, -265, and -268—are predicted to achieve high gastrointestinal absorption, and none of the series crosses the blood–brain barrier, reducing the risk of central nervous side-effects. Estero-254 and Estero-265 emerge as the most balanced prototypes, pairing strong target affinity with optimal lipophilicity (Log *p* ≈ 5.0), favorable solubility, an absence of mutagenic or hepatotoxic alerts, and moderate synthetic accessibility (SA ≈ 4.3–4.5). Estero-264 offers comparable pharmacodynamic performance but warrants structural tweaks to eliminate a predicted hepatotoxic liability. By contrast, Estero-253 and Estero-261 require mitigation of AMES-positive motifs, and the bulky RM-581 reference is penalized by its high molecular weight, conformational flexibility, and hepatotoxic signal.

Collectively, the data position Estero-254 and Estero-265 as front-runner leads, with Estero-264 as a promising secondary option once its safety profile is optimized. Their concurrent activity against PR, ER-α, and HER2 suggests a polypharmacological strategy with the potential to circumvent endocrine resistance. Follow-up efforts will focus on targeted chemical modifications, experimental ADME/Tox validation in cellular and microsomal systems, and formulation studies to confirm the in silico projections and translate these leads toward preclinical evaluation.

## Figures and Tables

**Table 1:** pIC_50_ Values and SMILES Representations of Steroidal Compounds in the QSAR Training Set.

Ligand	pIC_50_	Chemical Formula	Ligand	pIC_50_	Chemical Formula
Mol-1	6.04	C_28_H_32_IN_3_O_2_	Mol-27	5.65	C_32_H_40_N_2_O_4_
Mol-2	6.59	C_57_H_73_FN_2_O_2_	Mol-28	6.82	C_50_H_59_N_5_O_9_P
Mol-3	7.04	C_32_H_40_N_2_O_2_	Mol-29	6.66	C_42_H_52_N_4_O_4_
Mol-4	6.85	C_39_H_44_N_4_O_4_	Mol-30	6.64	C_42_H_52_N_4_O_5_
Mol-5	5.63	C_26_H_33_NO_3_	Mol-31	6.85	C_41_H_50_N_4_O_4_
Mol-6	5.59	C_25_H_35_N_3_O_3_S	Mol-32	6.85	C_41_H_50_N_4_O_5_
Mol-7	5.54	C_27_H_37_N_5_O_3_	Mol-33	6.92	C_41_H_50_N_4_O_6_S
Mol-8	5.49	C_46_H_55_N_5_O_7_	Mol-34	6.43	C_42_H_52_N_4_O_5_
Mol-9	5.74	C_46_H_55_N_5_O_7_	Mol-35	6.15	C_44_H_56_N_6_O_5_
Mol-10	5.82	C_33_H_44_N_2_O_5_	Mol-36	4.7	C_33_H_34_Cl_2_O_4_
Mol-11	5.4	C_34_H_46_N_2_O_6_	Mol-37	5.19	C_32_H_42_O_5_
Mol-12	6.09	C_31_H_39_ClN_2_O_3_	Mol-38	4.7	C_33_H_42_O_6_
Mol-13	6.04	C_32_H_39_F_3_N_2_O_3_	Mol-39	4.7	C_32_H_42_O_4_
Mol-14	5.92	C_31_H_39_N_3_O_5_	Mol-40	5.25	C_33_H_44_O_4_
Mol-15	5.95	C_31_H_39_N_3_O_5_	Mol-41	4.86	C_32_H_41_FO_4_
Mol-16	6.24	C_31_H_38_N_2_O_3_	Mol-42	4.82	C_32_H_41_BrO_4_
Mol-17	7.28	C_32_H_40_N_2_O_4_	Mol-43	4.75	C_33_H_44_O_7_
Mol-18	5.94	C_32_H_40_N_2_O_4_	Mol-44	5.14	C_36_H_46_O_8_
Mol-19	6.24	C_33_H_42_N_2_O_5_	Mol-45	6.14	C_32_H_42_O_6_
Mol-20	5.4	C_34_H_44_N_2_O_6_	Mol-46	6.39	C_25_H_29_N_3_O_2_S
Mol-21	5.4	C_31_H_39_ClN_2_O_3_	Mol-47	6.33	C_27_H_33_N_2_O_4_S
Mol-22	5.9	C_32_H_39_F_3_N_2_O_3_	Mol-48	6.23	C_34_H_37_N_2_O_3_S
Mol-23	5.65	C_31_H_39_N_3_O_5_	Mol-49	6.28	C_31_H_36_N_4_O
Mol-24	5.86	C_31_H_39_N_3_O_5_	Mol-50	6.42	C_24_H_32_N_4_OS
Mol-25	5.99	C_31_H_38_N_2_O_3_	Mol-51	6.28	C_34_H_39_N_5_O
Mol-26	7.38	C_32_H_40_N_2_O_4_	Mol-52	6.23	C_39_H_42_N_6_O

**Table 2:** Development of 3D-QSAR model.

Nº	DESCRIPTOR	Code
1	(−) VC_Q_AB_nCi_2_NS6_T_LGP[4–6]_e_MAS	A
2	(−) HM_F_BB_nCi_2_SS2_H_A_SRW_e_MAS	B
3	(+) K_TrC_AB_nCi_3_M26(M14)_SS2_T_LGA[6.0–7.0]_p_MID	C
4	(−) AC [1]_K_TrC_AB_nCi_3_M20(M15)_SS3_T_LGTP[10–11]_c_MID	D
5	(+) Number of HBond Donors	E
6	(−) N2_TrQB_AB_nCi_3_M26(M16)_SS2_T_LGA[0.0–1.0]_m-p_MID	F
7	(−) GV [3]_K_TrB_AB_nCi_3_M25(M13)_NS1_T_KA_p-s_MID	G
8	(+) TS [3]_K_TrC_AB_nCi_3_M27_NS0_T_KA_p_MID	H

**Table 3:** Statistical parameters of the QSAR model for steroidal molecules with activity against breast cancer in cell lines.

Metric	Hold-Out Value	LOO-CV Value
R (Correlation Coefficient)	0.974	0.929
R^2^ (Determination Coefficient)	0.949	0.862
R^2^_ext_	0.826	**-**
Q^2^ (Predictive Squared Correlation)	**-**	0.861
QMRF (Mean Squared Prediction Error)	0.091	0.075
RMSEP (Square root of MSE)	0.301	0.274
MAE_ext_	0.253	0.218
(R^2^ − R_0_^2^)/R^2^ < 0.1	0.0102	0.0752
(R^2^ − R_0_′^2^)/R^2^ < 0.1	0.0019	0.0752
|R_0_^2^ − R_0_′^2^| < 0.1	0.0078	0.0752
0.85 < K < 1.15	1.042	0.9286
0.85 < k’ < 1.15	0.959	0.9286

**Table 4:** Docking binding energies (ΔG, kcal/mol) calculated for the QSAR training-set molecules against the following targets: progesterone receptor (PDB 2W8Y), HER2 tyrosine kinase domain (PDB 7JXH), and estrogen-receptor-α ligand-binding domain (PDB 6VJD).

	Receptor						
Ligand Molecule	2W8Y	7JXH	6VJD	Ligand Molecule	2W8Y	7JXH	6VJD
Inhibitor substrate	−12.3	−9.0	−8.3	Mol-27	−6.0	−9.4	−8.8
Mol-1	−7.5	−10.7	−8.0	Mol-28	16.2	10.6	−8.6
Mol-2	1.8	−13.0	−9.9	Mol-29	−5.5	12.5	−8.3
Mol-3	22.5	−10.5	−8.4	Mol-30	−5.6	−13.2	−7.4
Mol-4	−6.4	−11.3	−8.3	Mol-31	21.3	−11.6	−8.0
Mol-5	−5.8	−9.5	−7.2	Mol-32	475.3	−11.9	−7.7
Mol-6	13.7	−8.5	−6.6	Mol-33	341.8	−11.2	−7.8
Mol-7	7.7	−9.6	−7.3	Mol-34	−5.0	−11.5	−8.3
Mol-8	−8.4	−11.8	−9.7	Mol-35	−0.6	−12.3	−9.3
Mol-9	−5.7	−12.2	−9.4	Mol-36	−0.4	−8.4	−6.6
Mol-10	2.8	−9.1	−6.8	Mol-37	−4.3	−9.7	−8.1
Mol-11	4.1	−8.8	−6.1	Mol-38	−2.4	−9.7	−8.7
Mol-12	−7.0	−8.0	−8.3	Mol-39	9.7	−9.7	−8.2
Mol-13	−8.2	−9.1	−9.2	Mol-40	14.6	−10.2	−8.5
Mol-14	−1.4	−8.7	−8.5	Mol-41	8.2	−9.6	−7.9
Mol-15	−3.9	−9.6	−8.6	Mol-42	−0.4	−9.6	−8.0
Mol-16	−7.5	−7.3	−5.2	Mol-43	−0.6	−9.5	−6.5
Mol-17	−0.2	9.6	−6.8	Mol-44	7.5	8.1	−6.5
Mol-18	−0.2	−9.3	−9.1	Mol-45	−3.3	−9.3	−7.6
Mol-19	2.5	−10.6	−9.0	Mol-46	2.6	−9.3	−6.7
Mol-20	5.8	−10.3	−8.5	Mol-47	20.9	−9.7	−6.8
Mol-21	0.1	−9.5	−9.1	Mol-48	11.0	−9.9	−7.3
Mol-22	−0.3	−11.0	−10.1	Mol-49	−1.5	−10.3	−7.3
Mol-23	−0.3	−10.9	−9.6	Mol-50	−6.5	−8.2	−6.3
Mol-24	2.5	−10.8	−6.8	Mol-51	−6.2	−10.5	−8.4
Mol-25	−7.3	−9.3	−8.4	Mol-52	3.0	−11.8	−9.1
Mol-26	1.2	−8.9	−9.2				

**Table 5:** Comparative residue–antagonist inhibitor interaction profile for the PR (2W8Y), HER2 (7JXH), and ER-α (6VJD) complexes.

Interaction Type	Amino Acid (Code + Number) of 2W8Y	Amino Acid (Code + Number) of 7JXH	Amino Acid (Code + Number) of 6VJD
Van der Waals	MET A:801, LEU A:763, LEU A:721, VAL A:760, CYS A:891, LEU A:718, LEU A:715, LEU A:797, PHE A:794, LEU A:887, LEU A:726, VAL A:912, GLN A:725	CYS A:805, LEU A:726, LEU A:800, LEU A:796, LEU A:785, LEU A:852, MET A:801, PHE A:1004, GLY A:804, GLY A:865, SER A:783, ARG A:784, THR A:798	LEU A:354, LEU A:525, LEU A:346, LEU A:349, LEU A:428, LEU A:384, ILE A:424, PRO A:535, MET A:343, MET A:388, MET A:421, GLY A:521
Conventional Hydrogen Bond	ARG A:766	-	HIS A:524, ARG A:394
Carbon Hydrogen Bond	TRP A:755	-	ASN A:532
Pi-Sigma	TYR A:890	ALA A:751	-
Pi-Sulfur	MET A:759	-	MET A:388
Amide-Pi Stacked	ASN A:719, GLY A:722	-	-
Salt Bridge	-	-	ASP A:351
Unfavorable Bump	-	MET A:774	-
Unfavorable Donor-Donor	-	-	ARG A:394
Halogen (Fluorine)	-	PHE A:864	-
Pi-Anion	-	ASP A:863	-
Pi-Lone Pair	-	THR A:862	-
Pi-Pi Stacked	-	-	PHE A:404
Alkyl	MET A:756, MET A:909, PHE A:778	LEU A:852, MET A:801	LEU A:391, LEU A:387, MET A:343
Pi-Alkyl	LEU A:718, LEU A:715, PHE A:794, VAL A:912	VAL A:734, LYS A:753, LEU A:852	TRP A:383, LEU A:354, MET A:421

**Table 6:** Docking binding energies (ΔG, kcal/mol) and predicted biological potency (pIC_50_) of the designed steroid ligands.

Ligand Molecule	Target Protein				Nomenclature	
	2W8Y	7JXH	6VJD	Ip50	IUPAC	Canonical Smile
RM-581	−5	−11.5	−8.3	6.4	C_40_H_46_N_4_O_4_	C[C@]12CC[C@H]3[C@H]([C@@H]1CC[C@]2(C#C)O)CCC4=CC(=C(C=C34)N5CCN(CC5)C(=O)[C@@H]6CCCN6C(=O)C7=NC8=CC=CC=C8C=C7)OC
Exemestane	−9.2	−8.7	−9.6	5.7	C_20_H_24_O_2_	C[C@]12CC[C@H]3[C@H]([C@@H]1CCC2=O)CC(=C)C4=CC(=O)C=C[C@]34C
Formestane	−9.6	−9.2	−9.6	9.2	C_19_H_26_O_3_	C[C@]12CCC(=O)C(=C1CC[C@@H]3[C@@H]2CC[C@]4([C@H]3CCC4=O)C)O
Estero_253	−7.6	−10.1	−7.1	7.2	C_29_H_30_ClNO_2_	CC1=CC2=C(C=C1)N=C(Cl)C(=C2)COC3=CC=C4C5CCC6(C)C(CCC6=O)C5CCC4=C3
Estero_254	−7	−10.2	−7.3	7.4	C_29_H_30_ClNO_3_	COC1=CC2=C(C=C1)N=C(Cl)C(=C2)COC3=CC=C4C5CCC6(C)C(CCC6=O)C5CCC4=C3
Estero_255	−9.4	−11.1	−9.8	7.3	C_28_H_28_ClNO_2_	CC12CCC3C(CCC4=C3C=CC(=C4)OCC5=CC6=C(C=CC=C6)N=C5Cl)C1CCC2=O
Estero_261	−9.2	−10.2	−9.6	7.4	C_29_H_30_ClNO_2_	CC1=CC=CC2=CC(=C(Cl)N=C12)COC3=CC4=C(C=C3)C5CCC6(C)C(CCC6=O)C5CC4
Estero_264	−8.4	−10.8	−10.8	7.2	C_29_H_31_NO_4_	COC1=CC=C2NC(=O)C(=CC2=C1)COC3=CC=C4C5CCC6(C)C(CCC6=O)C5CCC4=C3
Estero_265	−8.5	−10.7	−9	7.2	C_29_H_31_NO_3_	CC1=CC=C2NC(=O)C(=CC2=C1)COC3=CC=C4C5CCC6(C)C(CCC6=O)C5CCC4=C3
Estero_268	−11.2	−10.9	−8.3	7.5	C_28_H_28_FNO_3_	CC12CCC3C(CCC4=CC(=CC=C34)OCC5=CC6=CC(=CC=C6NC5=O)F)C1CCC2=O

**Table 7:** Physicochemical properties, lipophilicity, solubility, pharmacokinetics, drug-likeness, and predicted toxicity of steroidal ligands E-253–E-268, RM-581, and the reference inhibitors Exemestane and Formestane.

Properties	Ligands	E-253	E-254	E-255	E-261	E-264	E-265	E-268	RM-581	Exemestane	Formestane
Physico-chemical Properties	MW (g/mol)	460.017	476.01	445.98	460.01	457.56	441.56	445.53	646.82	296.4	302.41
	Heavy atoms	33	34	32	33	34	33	33	48	22	22
	Arom. Heavy atoms	16	16	16	16	16	16	16	16	0	0
	Rotatable atoms	3	4	3	3	4	3	3	6	0	0
	H-bond acceptors	3	4	3	3	4	3	4	5	2	3
	H-bond donors	0	0	0	0	1	1	1	1	0	1
Lipophilicity	Consensus Log *p*	6.11	5.76	5.78	6.09	4.8	5.18	5.14	4.78	3.51	3.09
Water solubility	Log S (ESOL)	−6.97	−6.75	−6.67	−6.97	−5.5	−5.72	−5.58	−7.38	−3.61	−3.35
Pharmacokinetics	GI absorption	Low	High	High	Low	High	High	High	High	High	High
	BBB permeant	No	No	No	No	No	No	No	No	Yes	Yes
Druglikeness	Lipinski. Violation	1	1	1	1	0	1	1	1	0	0
Medicinal Chemistry	Synth. accessibility	4.43	4.48	4.31	4.48	4.52	4.47	4.37	5.92	5.03	4.5
Toxicity	AMES toxicity	Yes	No	Yes	Yes	No	No	No	No	No	No
	Oral Rat Acute Toxicity LD50 (mol/kg)	2.991	2.983	2.921	2.955	2.415	2.43	2.175	3.171	1.689	1.998
	Oral Rat Chronic Toxicity (LOAEL)	1.688	1.26	1.858	1.596	1.273	1.677	1.167	1.791	1.776	1.747
	Hepatotoxicity	No	No	No	No	Yes	No	No	Yes	No	No
	Skin Sensitization	No	No	No	No	No	No	No	No	No	No
	Predicted Toxicity Class	5	5	5	5	5	5	5	5	5	6
	Average similarity (%)	58.79	58.57	58.99	57.98	54.67	55.05	52.22	54.86	70.97	92.68
	Prediction accuracy (%)	67.38	67.38	67.38	67.38	67.38	67.38	67.38	67.38	69.26	72.9

**Table 8:** Molecular Interaction Profile of the designed steroidal ligands bound to the progesterone-receptor ligand-binding domain (PDB 2W8Y).

Ligand	Van Der Waals	Conventional H-Bond	Carbon H-Bond	π-Donor H-Bond	Unfavorable Bump	Unfavorable Acceptor-Acceptor	Halogen (Fluorine)	π-Stack/π-T-shaped	π–Sulfur	Alkyl π–Alkyl
E-253	11	0	0	0	0	0	0	0	1	9
E-254	15	1	0	1	0	0	0	0	3	9
E-255	15	1	0	0	5	1	0	0	3	8
E-261	11	1	0	0	5	0	0	3	4	9
E-264	11	0	1	0	4	1	0	0	1	7
E-265	16	1	0	1	2	0	0	0	2	8
E-268	22	0	0	0	0	0	5	2	0	5

**Table 9:** Molecular Interaction Profile of the designed steroidal ligands with the HER2 tyrosine kinase domain (PDB 7JXH).

Ligand	Van Der Waals	Carbon H-Bond	Conventional H-Bond	π-Donor H-Bond	π–Anion/Attractive	Halogen (Fluorine)	π–Sigma	π–π T-Shaped	π–π Stacked	Unfavorable Donor-Donor	Alkyl + π–Alkyl
E-253	7	1	0	0	2	0	0	1	0	0	3
E-254	13	0	3	1	2	0	0	0	0	0	9
E-255	15	1	0	0	0	0	0	0	0	0	5
E-261	11	0	0	0	0	0	2	0	0	0	9
E-264	13	0	0	0	0	0	2	0	0	0	8
E-265	15	0	4	0	5	0	0	0	5	2	0
E-268	15	0	6	0	2	2	0	0	7	2	0

**Table 10:** Molecular Interaction Profile of the designed steroidal ligands bound to the ER-α ligand-binding domain (PDB 6VJD).

Ligand	Van Der Waals	Carbon H-Bond	π–Anion	π–Sigma	π–Sulfur	π-π	Alkyl + π–Alkyl
E-253	12	0	1	2	1	4	7
E-254	13	1	1	2	1	5	9
E-255	15	1	0	2	1	6	8
E-261	14	0	0	2	2	6	9
E-264	12	1	0	2	1	7	9
E-265	13	0	0	2	1	7	10
E-268	14	0	1	2	1	6	9

## Data Availability

Data are contained within the article.

## Supplementary Materials

The following supporting information can be downloaded at https://www.mdpi.com/article/10.3390/ijms26157477/s1.
