# Supplementary material for: Multitarget Design of Steroidal Inhibitors Against Hormone-Dependent Breast Cancer: An Integrated In Silico Approach
Source: Int J Mol Sci. 2025 Aug 2;26(15):7477. doi: 10.3390/ijms26157477 (PMC12348013; doi:10.3390/ijms26157477)
Supplement: Supplementary file 1 [file ijms-26-07477-s001.zip › ijms-3755776-supplementary.pdf]

Multitarget Design of Steroidal Inhibitors Against Hormone-Dependent Breast Cancer: An Integrated *In Silico* Approach.

Supplementary Material

Table S1. pIC_{50} Values and SMILES Representations of Steroidal Compounds in the QSAR Training Set.

Ligand molecule	PICL50	Smiles code
Mol-1	6.04	O=C1/C(C[C@@]2([H])[C@]3([H])CC=C4C[C@@H](O)CC[C@]4(C)[C@@]3([H])CC[C@]12C)=C/C5=CN(C6=CC=C(I)C=C6)N=N5
Mol-2	6.59	O=C([C@@](C1)2N(C)[C@@H](C3=CC=C(/C=C4CC5C6CC=C7C[C@@H](C)CC[C@]7(C)C6CC[C@]5(C)C\4)C=C3)C81CC9C%10CC=C%11C[C@@H](O)CC[C@]%11(C)C%10CC[C@]9(C)C8)NC%12=C2C=CC(F)=C%12
Mol-3	7.04	O[C@H]1CC[C@]2(C)C3CC[C@]4(C)[C@@H](C5=NN(C6=CC=CC=C6)C(C7=CC=CO7)C5)CCC4C3CC=C2C1
Mol-4	6.85	O=C(N1CCN(C2=C(O)C=C3CC[C@@]4([H])[C@]5([H])CC[C@@](O)(C#C)[C@@]5(C)CC[C@]4([H])C3=C2)CC1)[C@H]6N(C(C7=NC8=CC=CC=C8C=C7)=O)CCC6
Mol-5	5.63	O=C(O)C1=CC=CC=C1NC2CCC3C4CCC5=CC(CC[C@]5(C)C4CC[C@]23C)=O
Mol-6	5.59	CC(O[C@H](C[C@]1([H])CCC2C3CC/4)CC[C@]1(C)C2CC[C@]3(C)C4=N\C(NC5=S)=CC(N5)=O)=O
Mol-7	5.54	CC(O[C@H](C[C@]1([H])CCC2C3CC/4)CC[C@]1(C)C2CC[C@]3(C)C4=N\C(N=C5N6C(C)=NN5)=CC6=O)=O
Mol-8	5.49	CC(O[C@H](C[C@]1([H])CCC2C3CC/4)CC[C@]1(C)C2CC[C@]3(C)C4=N\C(N=C(N5N=C(/C=C/C6=CC=C(O)C(OC)=C6)CC5/C=C/C7=CC=C(O)C(OC)=C7)N8)=CC8=O)=O
Mol-9	5.74	CC(O[C@H](C[C@]1([H])CCC2C3CC/4)CC[C@]1(C)C2CC[C@]3(C)C4=N\C(N=C(N5N=C(/C=C/C6=CC=C(O)C(OC)=C6)CC5/C=C/C7=CC=C(O)C(OC)=C7)N8)=CC8=

		O)=O
Mol-10	5.82	O=C([C@@H]1CCC2C3CCC4=CC(CC[C@]4(C)C3CC[C@]12C)=O)NCCNC(/C=C/C5=CC=C(OC)C(OC)=C5)=O
Mol-11	5.4	O=C([C@@H]1CCC2C3CCC4=CC(CC[C@]4(C)C3CC[C@]12C)=O)NCCNC(/C=C/C5=CC(OC)=C(OC)C(OC)=C5)=O
Mol-12	6.09	O=C([C@@H]1CCC2C3CCC4=CC(CC[C@]4(C)C3CC[C@]12C)=O)NCCNC(/C=C/C5=CC=C(Cl)C=C5)=O
Mol-13	6.04	O=C([C@@H]1CCC2C3CCC4=CC(CC[C@]4(C)C3CC[C@]12C)=O)NCCNC(/C=C/C5=CC=C(C(F)(F)F)C=C5)=O
Mol-14	5.92	O=C([C@@H]1CCC2C3CCC4=CC(CC[C@]4(C)C3CC[C@]12C)=O)NCCNC(/C=C/C5=CC=C([N+]([O-])=O)C=C5)=O
Mol-15	5.95	O=C([C@@H]1CCC2C3CCC4=CC(CC[C@]4(C)C3CC[C@]12C)=O)NCCNC(/C=C/C5=CC=CC([N+]([O-])=O)=C5)=O
Mol-16	6.24	O=C([C@@H]1CCC2C3CCC4=CC(C=C[C@]4(C)C3CC[C@]12C)=O)NCCNC(C=CC5=CC=CC=C5)=O
Mol-17	7.28	O=C([C@@H]1CCC2C3CCC4=CC(C=C[C@]4(C)C3CC[C@]12C)=O)NCCNC(/C=C/C5=CC=CC=C5OC)=O
Mol-18	5.94	O=C([C@@H]1CCC2C3CCC4=CC(C=C[C@]4(C)C3CC[C@]12C)=O)NCCNC(/C=C/C5=CC=C(OC)C=C5)=O
Mol-19	6.24	O=C([C@@H]1CCC2C3CCC4=CC(C=C[C@]4(C)C3CC[C@]12C)=O)NCCNC(/C=C/C5=CC=C(OC)C(OC)=C5)=O
Mol-20	5.4	O=C([C@@H]1CCC2C3CCC4=CC(C=C[C@]4(C)C3CC[C@]12C)=O)NCCNC(/C=C/C5=CC(OC)=C(OC)C(OC)=C5)=O
Mol-21	5.4	O=C([C@@H]1CCC2C3CCC4=CC(C=C[C@]4(C)C3CC[C@]12C)=O)NCCNC(/C=C/C5=CC=C(Cl)C=C5)=O
Mol-22	5.9	O=C([C@@H]1CCC2C3CCC4=CC(C=C[C@]4(C)C3CC[C@]12C)=O)NCCNC(/C=C/C5=CC=C(C(F)(F)F)C=C5)=O
Mol-23	5.65	O=C([C@@H]1CCC2C3CCC4=CC(C=C[C@]4(C)C3CC[C@]12C)=

		O)NCCNC(/C=C/C5=CC=C([N+]([O-])=O)C=C5)=O
Mol-24	5.86	O=C([C@@H]1CCC2C3CCC4=CC(C=C[C@]4(C)C3CC[C@]12C)=O)NCCNC(/C=C/C5=CC=CC([N+]([O-])=O)=C5)=O
Mol-25	5.99	O=C([C@@H]1CCC2C3CCC4=CC(CC[C@]4(C)C3CC[C@]12C)=O)NCCNC(C=CC5=CC=CC=C5)=O
Mol-26	7.38	O=C([C@@H]1CCC2C3CCC4=CC(CC[C@]4(C)C3CC[C@]12C)=O)NCCNC(/C=C/C5=CC=CC=C5OC)=O
Mol-27	5.65	O=C([C@@H]1CCC2C3CCC4=CC(CC[C@]4(C)C3CC[C@]12C)=O)NCCNC(/C=C/C5=CC=C(OC)C=C5)=O
Mol-28	6.82	O=P(O)(OC(C(N1CCN(C([C@H]2N(C(C3=NC4=CC=CC=C4C=C3)=O)CCC2)=O)CC1)=C5)=CC6=C5[C@@]7([H])CC[C@]8(C)[C@](O)(C#C)CC[C@@]8([H])[C@]7([H])CC6)O
Mol-29	6.66	O=C(C1=CC2=C(CC[C@@]3([H])[C@]4([H])CC[C@@](O)(C#C)[C@]4(CC[C@@]32[H])C)C=C1OC)N5CCN(C([C@H]6N(C(C7=NC8=CC=CC=C8C=C7)=O)CCC6)=O)CC5
Mol-30	6.64	O=C(N1CCN(CC2=CC3=C(CC[C@@]4([H])[C@]5([H])CC[C@@](O)(C#C)[C@]5(CC[C@@]43[H])C)C=C2OC)CC1)[C@H]6N(C(C7=NC8=CC=CC=C8C=C7)=O)CCC6
Mol-31	6.85	O=C(N1CCN(C2=CC3=C(CC[C@@]4([H])[C@]5([H])CC[C@@](O)(C#C)[C@]5(CC[C@@]43[H])C)C=C2O)CC1)[C@H]6N(C(C7=NC8=CC=CC=C8C=C7)=O)CCC6
Mol-32	6.85	O=C([O-])CC(C(N1CCN(C([C@H]2N(C(C3=NC4=CC=CC=C4C=C3)=O)CCC2)=O)CC1)=C5)=CC6=C5[C@@]7([H])CC[C@]8(C)[C@](O)(C#C)CC[C@@]8([H])[C@]7([H])CC6
Mol-33	6.92	O=S([O-])(NC(C(N1CCN(C([C@H]2N(C(C3=NC4=CC=CC=C4C=C3)=O)CCC2)=O)CC1)=C5)=CC6=C5[C@@]7([H])CC[C@]8(C)[C@](O)(C#C)CC[C@@]8([H])[C@]7([H])CC6)=O
Mol-34	6.43	O=C(N1CCN(C2=C(OC)C=C3CC[C@@]4([H])[C@]5([H])CC[C@@](O)(C#C)[C@@]5(C)CC[C@]4([H])

		C3=C2)CC1)[C@H]6N(C(C7=NC8=CC=CC=C8C=C7)=O)CCC6
Mol-35	6.15	O=C([C@H]1N(C(C2=NC3=CC(N(C)C)=CC=C3C=C2)=O)CCC1)N4CCN(C5=CC6=C(CC[C@@]7([H])[C@]8([H])CC[C@]([C@]8(CC[C@@]76[H])C)(O)C#C)C=C5OC)CC4
Mol-36	4.7	C=C([C@]1(OC)C=C(C2=CC=C(Cl)C=C2Cl)C3=C(CC[C@]4([H])[C@]5([H])CCC[C@]5(C)CC[C@]43[H])C1)C(CC(O)=O)=O
Mol-37	5.19	O=C(C1=CC=C(O)C(OC)=C1)CCC2=C(OC)C=C3CC[C@@]4([H])[C@@]5([H])CC[C@H](O)[C@@]5(C)CC[C@]4([H])C3=C2
Mol-38	4.7	O=C(C1=CC=C(OCO2)C2=C1)/C=C/C3=C(OC)C=C4CC[C@@]5([H])[C@@]6([H])CC[C@H](O)[C@@]6(C)CC[C@]5([H])C4=C3
Mol-39	4.7	O=C(C1=CC=C(C)C=C1)/C=C/C2=C(OC)C=C3CC[C@@]4([H])[C@@]5([H])CC[C@H](O)[C@@]5(C)CC[C@]4([H])C3=C2
Mol-40	5.25	O=C(C1=CC=C(C)C=C1C)/C=C/C2=C(OC)C=C3CC[C@@]4([H])[C@@]5([H])CC[C@H](O)[C@@]5(C)CC[C@]4([H])C3=C2
Mol-41	4.86	O=C(C1=CC=C(F)C=C1)/C=C/C2=C(OC)C=C3CC[C@@]4([H])[C@@]5([H])CC[C@H](O)[C@@]5(C)CC[C@]4([H])C3=C2
Mol-42	4.82	O=C(C1=CC=C(Br)C=C1)/C=C/C2=C(OC)C=C3CC[C@@]4([H])[C@@]5([H])CC[C@H](O)[C@@]5(C)CC[C@]4([H])C3=C2
Mol-43	4.75	O=C(C1=CC(OC)=C(OC)C(OC)=C1)CCC2=C(OC)C=C3CC[C@@]4([H])[C@@]5([H])CC[C@H](O)[C@@]5(C)CC[C@]4([H])C3=C2
Mol-44	5.14	C=C([C@]1(OC)C=C(C2=CC(OC)=C(OC)C(OC)=C2)C3=C(CC[C@]4([H])[C@]5([H])CCC[C@]5(C)CC[C@]43[H])C1)C(CC(O)=O)=O
Mol-45	6.14	O=C(C1=CC=C(OC)C(OC)=C1)/C=C/C2=C(OC)C=C3CC[C@@]4([H])[C@@]5([H])CC[C@H](O)[C@@]5(C)CC[C@]4([H])C3=C2
Mol-46	6.39	N#CC1=C(S)N=C(C2CC[C@@]3([H])[C@]4([H])CCC5=CC(CC[C@]5(C)[C@@]4([H])CC[C@]23C)=O)C=C1
Mol-47	6.33	O=C(C1=C(N)C2=CC=C(C3CC[C@@]4([H])[C@]5([H])CCC6=CC(CC[C@]6(C)[C@@]5([H])CC[C@]34

		C)=O)N=C2S1)OCC
Mol-48	6.23	C[C@@]12C(C3=CC=C(C(N)=C(C(C)=O)S4)C4=N3)CC[C@@]1([H])[C@]5([H])CCC6=CC(CC[C@]6(C)[C@@]5([H])CC2)=O
Mol-49	6.28	C[C@@]12C(C3=NC4=NNC(N)=C4C=C3)CC[C@@]1([H])[C@]5([H])CCC6=CC(CC[C@]6(C)[C@@]5([H])CC2)=O
Mol-50	6.42	S=C(N/N=C(C1CC[C@@]2([H])[C@]3([H])CCC4=CC(CC[C@]4(C)[C@@]3([H])CC[C@]12C)=O)/C)N
Mol-51	6.28	C[C@@]12C(/C(C)=N\NC3=NC(/N=N/C4=CC=CC=C4)=C(C)S3)CC[C@@]1([H])[C@]5([H])CCC6=CC(CC[C@]6(C)[C@@]5([H])CC2)=O
Mol-52	6.23	C[C@@]12C(/C(C)=N\NC3=NC(/N=N/C4=CC=CC=C4)=C(C5=CC=CC=C5)S3)CC[C@@]1([H])[C@]6([H])CCC7=CC(CC[C@]7(C)[C@@]6([H])CC2)=O

Table S2. Molecules design by means of model 1.

**Molecule**<br>**SMILES_explicit_H**	
ESTERO-1.mol	[H]O[C@@]1([H])C([H])=C2C([H])([H])C([H])([H])[C@@]3([H])[C@]4([H])C([H])([H])C([H])([H])[C@]([H])(C(=O)/C([H])=C(\[H])c5c(Cl)nc6c([H])c([H])c(Cl)c([H])c6c5[H])[C@@]4(C([H])([H])[H])C([H])([H])C([H])([H])[C@]3([H])[C@@]2(C([H])([H])[H])C([H])([H])C1([H])[H]
ESTERO-2.mol	[H]O[C@@]1([H])C([H])=C2C([H])([H])C([H])([H])[C@@]3([H])[C@]4([H])C([H])([H])C([H])([H])[C@]([H])(C(=O)/C([H])=C(\[H])c5c(Cl)nc6c([H])c([H])c(OC([H])([H])[H])c([H])c6c5[H])[C@@]4(C([H])([H])[H])C([H])([H])C([H])([H])[C@]3([H])[C@@]2(C([H])([H])[H])C([H])([H])C1([H])[H]
ESTERO-3.mol	[H]O[C@@]1([H])C([H])=C2C([H])([H])C([H])([H])[C@@]3([H])[C@]4([H])C([H])([H])C([H])([H])[C@]([H])(C(=O)/C([H])=C(\[H])c5c(Cl)nc6c([H])c([H])c(C(F)(F)F)c([H])c6c5[H])[C@@]4(C([H])([H])[H])C([H])([H])C([H])([H])[C@]3([H])[C@@]2(C([H])([H])[H])C([H])([H])C1([H])[H]
ESTERO-4.mol	[H]O[C@@]1([H])C([H])=C2C([H])([H])C([H])([H])[C@@]3([H])[C@]4([H])C([H])([H])C([H])([H])[C@]([H])(C(=O)/C([H])=C(\[H])c5c(Cl)nc6c([H])c([H])c(C([H])([H])[H])c([H])c6c5[H])[C@@]4(C([H])([H])[H])C([H])([H])C([H])([H])[C@]3([H])[C@@]2(C([H])([H])[H])C([H])([H])C1([H])[H]
ESTERO-5.mol	[H]O[C@@]1([H])C([H])=C2C([H])([H])C([H])([H])[C@@]3([H])[C@]4([H])C([H])([H])C([H])([H])[C@]([H])(C(=O)/C([H])=C(\[H])c5c(Cl)nc6c([H])c([H])c([H])c([H])c6c5[H])[C@@]4(C([H])([H])[H])C([H])([H])C([H])([H])[C@]3([H])[C@@]2(C([H])([H])[H])C([H])([H])C1([H])[H]
ESTERO-6.mol	[H]O[C@@]1([H])C([H])=C2C([H])([H])C([H])([H])[C@@]3([H])[C@]4([H])C([H])([H])C([H])([H])[C@]([H])(C(=O)/C([H])=C(\[H])c5c(Cl)nc6c([H])c([H])c(SC([H])([H])[H])c([H])c6c5[H])[C@@]4(C([H])([H])[H])C([H])([H])C([H])([H])[C@]3([H])[C@@]2(C([H])([H])[H])C([H])([H])C1([H])[H]

ESTERO-7.mol	[H]O[C@@]1([H])C([H])=C2C([H])([H])C([H])([H])[C@@]3([H])[C@]4([H])C([H])([H])C([H])([H])[C@]([H])(C(=O)/C([H])=C(\[H])c5c(Cl)nc6c([H])c(Cl)c([H])c([H])c6c5[H])[C@@]4(C([H])([H])[H])C([H])([H])C([H])([H])[C@]3([H])[C@@]2(C([H])([H])[H])C([H])([H])C1([H])[H]
ESTERO-8.mol	[H]C1=C2C([H])([H])C([H])([H])[C@@]3([H])[C@]4([H])C([H])([H])C([H])([H])[C@]([H])(C(=O)C([H])([H])[H])[C@@]4(C([H])([H])[H])C([H])([H])C([H])([H])[C@]3([H])[C@@]2(C([H])([H])[H])C([H])([H])C([H])([H])[C@@]1([H])OC([H])([H])c1c(Cl)nc2c([H])c([H])c([H])c([H])c2c1[H]
ESTERO-9.mol	[H]O[C@@]1([H])C([H])=C2C([H])([H])C([H])([H])[C@@]3([H])[C@]4([H])C([H])([H])C([H])([H])[C@]([H])(C(=O)/C([H])=C(\[H])c5c(Cl)nc6c([H])c(C(F)(F)F)c([H])c([H])c6c5[H])[C@@]4(C([H])([H])[H])C([H])([H])C([H])([H])[C@]3([H])[C@@]2(C([H])([H])[H])C([H])([H])C1([H])[H]
ESTERO-10.mol	[H]O[C@@]1([H])C([H])=C2C([H])([H])C([H])([H])[C@@]3([H])[C@]4([H])C([H])([H])C([H])([H])[C@]([H])(C(=O)/C([H])=C(\[H])c5c(Cl)nc6c([H])c([H])c([H])c([H])c6c5[H])[C@@]4(C([H])([H])[H])C([H])([H])C([H])([H])[C@]3([H])[C@@]2(C([H])([H])[H])C([H])([H])C1([H])[H]
ESTERO-11.mol	[H]O[C@@]1([H])C([H])=C2C([H])([H])C([H])([H])[C@@]3([H])[C@]4([H])C([H])([H])C([H])([H])[C@]([H])(C(=O)/C([H])=C(\[H])c5c(Cl)nc6c([H])c(SC([H])([H])[H])c([H])c([H])c6c5[H])[C@@]4(C([H])([H])[H])C([H])([H])C([H])([H])[C@]3([H])[C@@]2(C([H])([H])[H])C([H])([H])C1([H])[H]
ESTERO-12.mol	[H]O[C@@]1([H])C([H])=C2C([H])([H])C([H])([H])[C@@]3([H])[C@]4([H])C([H])([H])C([H])([H])[C@]([H])(C(=O)/C([H])=C(\[H])C5=C([H])[C@]6([H])C([H])=C(Cl)C([H])=C([H])[C@]6([H])N([H])C5=O)[C@@]4(C([H])([H])[H])C([H])([H])C([H])([H])[C@]3([H])[C@@]2(C([H])([H])[H])C([H])([H])C1([H])[H]
ESTERO-13.mol	[H]O[C@@]1([H])C([H])=C2C([H])([H])C([H])([H])[C@@]3([H])[C@]4([H])C([H])([H])C([H])([H])[C@]([H])(C(=O)/C([H])=C(\[H])C5=C([H])[C@]6([H])C([H])=C(OC([H])([H])[H])C([H])=C([H])[C@]6([H])N([H])C5=O)[C@@]4(C([H])([H])[H])C([H])([H])C([H])([H])[C@]3([H])[C@@]2(C([H])([H])[H])C([H])([H])C1([H])[H]
ESTERO-14.mol	[H]O[C@@]1([H])C([H])=C2C([H])([H])C([H])([H])[C@@]3([H])[C@]4([H])C([H])([H])C([H])([H])[C@]([H])(C(=O)/C([H])=C(\[H])C5=C([H])[C@]6([H])C([H])=C(C([H])([H])[H])C([H])=C([H])[C@]6([H])N([H])C5=O)[C@@]4(C([H])([H])[H])C([H])([H])C([H])([H])[C@]3([H])[C@@]2(C([H])([H])[H])C([H])([H])C1([H])[H]
ESTERO-15.mol	[H]O[C@@]1([H])C([H])=C2C([H])([H])C([H])([H])[C@@]3([H])[C@]4([H])C([H])([H])C([H])([H])[C@]([H])(C(=O)/C([H])=C(\[H])C5=C([H])[C@]6([H])C([H])=C([H])C([H])=C([H])[C@]6([H])N([H])C5=O)[C@@]4(C([H])([H])[H])C([H])([H])C([H])([H])[C@]3([H])[C@@]2(C([H])([H])[H])C([H])([H])C1([H])[H]
ESTERO-16.mol	[H]O[C@@]1([H])C([H])=C2C([H])([H])C([H])([H])[C@@]3([H])[C@]4([H])C([H])([H])C([H])([H])[C@]([H])(C(=O)/C([H])=C(\[H])C5=C([H])[C@]6([H])C([H])=C(SC([H])([H])[H])C([H])=C([H])[C@]6([H])N([H])C5=O)[C@@]4(C([H])([H])[H])C([H])([H])C([H])([H])[C@]3([H])[C@@]2(C([H])([H])[H])C([H])([H])C1([H])[H]
ESTERO-17.mol	[H]O[C@@]1([H])C([H])=C2C([H])([H])C([H])([H])[C@@]3([H])[C@]4([H])C([H])([H])C([H])([H])[C@]([H])(C(=O)/C([H])=C(\[H])C5=C([H])[C@]6([H])C([H])=C(Br)C([H])=C([H])[C@]6([H])N([H])C5=O)[C@@]4(C([H])([H])[H])C([H])([H])C([H])([H])[C@]3([H])[C@@]2(C([H])([H])[H])C([H])([H])C1([H])[H]
ESTERO-18.mol	[H]O[C@@]1([H])C([H])=C2C([H])([H])C([H])([H])[C@@]3([H])[C@]4([H])C([H])([H])C([H])([H])[C@]([H])(C(=O)/C([H])=C(\[H])C5=C([H])[C@]6([H])C([H])=C([H])C(Cl)=C([H])[C@]6([H])N([H])C5=O)[C@@]4(C([H])([H])[H])C([H])([H])C([H])([H])[C@]3([H])[C@@]2(C([H])([H])[H])C([H])([H])C1([H])[H]
ESTERO-19.mol	[H]O[C@@]1([H])C([H])=C2C([H])([H])C([H])([H])[C@@]3([H])[C@]4([H])C([H])([H])C([H])([H])[C@]([H])(C(=O)/C([H])=C(\[H])C5=C([H])[C@]6([H])C([H])=C([H])C(OC([H])([H])[H])=C([H])[C@]6([H])N([H])C5=O)[C@@]4(C([H])([H])[H])C([H])([H])C([H])([H])[C@]3([H])[C@@]2(C([H])([H])[H])C([H])([H])C1([H])[H]

ESTERO-20.mol	[H]O[C@@]1([H])C([H])=C2C([H])([H])C([H])([H])[C@@]3([H])[C@]4([H])C([H])([H])C([H])([H])[C@]([H])(C(=O)/C([H])=C(\[H])C5=C([H])[C@]6([H])C([H])=C([H])C(C(F)(F)F)=C([H])[C@]6([H])N([H])C5=O)[C@@]4(C([H])([H])[H])C([H])([H])C([H])([H])[C@]3([H])[C@@]2(C([H])([H])[H])C([H])([H])C1([H])[H]
ESTERO-21.mol	[H]O[C@@]1([H])C([H])=C2C([H])([H])C([H])([H])[C@@]3([H])[C@]4([H])C([H])([H])C([H])([H])[C@]([H])(C(=O)/C([H])=C(\[H])C5=C([H])[C@]6([H])C([H])=C([H])C(C([H])([H])[H])=C([H])[C@]6([H])N([H])C5=O)[C@@]4(C([H])([H])[H])C([H])([H])C([H])([H])[C@]3([H])[C@@]2(C([H])([H])[H])C([H])([H])C1([H])[H]
ESTERO-22.mol	[H]O[C@@]1([H])C([H])=C2C([H])([H])C([H])([H])[C@@]3([H])[C@]4([H])C([H])([H])C([H])([H])[C@]([H])(C(=O)/C([H])=C(\[H])C5=C([H])[C@]6([H])C([H])=C([H])C(C([H])([H])[H])=C([H])[C@]6([H])N([H])C5=O)[C@@]4(C([H])([H])[H])C([H])([H])C([H])([H])[C@]3([H])[C@@]2(C([H])([H])[H])C([H])([H])C1([H])[H]
ESTERO-23.mol	[H]O[C@@]1([H])C([H])=C2C([H])([H])C([H])([H])[C@@]3([H])[C@]4([H])C([H])([H])C([H])([H])[C@]([H])(C(=O)/C([H])=C(\[H])C5=C([H])[C@]6([H])C([H])=C([H])C([H])=C([H])[C@]6([H])N([H])C5=O)[C@@]4(C([H])([H])[H])C([H])([H])C([H])([H])[C@]3([H])[C@@]2(C([H])([H])[H])C([H])([H])C1([H])[H]
ESTERO-24.mol	[H]O[C@@]1([H])C([H])=C2C([H])([H])C([H])([H])[C@@]3([H])[C@]4([H])C([H])([H])C([H])([H])[C@]([H])(C(=O)/C([H])=C(\[H])C5=C([H])[C@]6([H])C([H])=C([H])C(SC([H])([H])[H])=C([H])[C@]6([H])N([H])C5=O)[C@@]4(C([H])([H])[H])C([H])([H])C([H])([H])[C@]3([H])[C@@]2(C([H])([H])[H])C([H])([H])C1([H])[H]
ESTERO-25.mol	[H]O[C@@]1([H])C([H])=C2C([H])([H])C([H])([H])[C@@]3([H])[C@]4([H])C([H])([H])C([H])([H])[C@]([H])(C(=O)/C([H])=C(\[H])C5=C([H])[C@]6([H])C([H])=C([H])C(Br)=C([H])[C@]6([H])N([H])C5=O)[C@@]4(C([H])([H])[H])C([H])([H])C([H])([H])[C@]3([H])[C@@]2(C([H])([H])[H])C([H])([H])C1([H])[H]
ESTERO-26.mol	[H]O[C@@]1([H])C([H])=C2C([H])([H])C([H])([H])[C@@]3([H])[C@]4([H])C([H])([H])C([H])([H])[C@]([H])(C(=O)/C([H])=C(\[H])c5c(OC([H])([H])C([H])([H])[H])nc6c([H])c(Cl)c([H])c([H])c6c5[H])[C@@]4(C([H])([H])[H])C([H])([H])C([H])([H])[C@]3([H])[C@@]2(C([H])([H])[H])C([H])([H])C1([H])[H]
ESTERO-27.mol	[H]O[C@@]1([H])C([H])=C2C([H])([H])C([H])([H])[C@@]3([H])[C@]4([H])C([H])([H])C([H])([H])[C@]([H])(C(=O)/C([H])=C(\[H])c5c(OC([H])([H])C([H])([H])[H])nc6c([H])c(C(F)(F)F)c([H])c([H])c6c5[H])[C@@]4(C([H])([H])[H])C([H])([H])C([H])([H])[C@]3([H])[C@@]2(C([H])([H])[H])C([H])([H])C1([H])[H]
ESTERO-28.mol	[H]O[C@@]1([H])C([H])=C2C([H])([H])C([H])([H])[C@@]3([H])[C@]4([H])C([H])([H])C([H])([H])[C@]([H])(C(=O)/C([H])=C(\[H])c5c(OC([H])([H])C([H])([H])[H])nc6c([H])c(C([H])([H])[H])c([H])c([H])c6c5[H])[C@@]4(C([H])([H])[H])C([H])([H])C([H])([H])[C@]3([H])[C@@]2(C([H])([H])[H])C([H])([H])C1([H])[H]
ESTERO-29.mol	[H]O[C@@]1([H])C([H])=C2C([H])([H])C([H])([H])[C@@]3([H])[C@]4([H])C([H])([H])C([H])([H])[C@]([H])(C(=O)/C([H])=C(\[H])c5c(OC([H])([H])C([H])([H])[H])nc6c([H])c([H])c([H])c([H])c6c5[H])[C@@]4(C([H])([H])[H])C([H])([H])C([H])([H])[C@]3([H])[C@@]2(C([H])([H])[H])C([H])([H])C1([H])[H]
ESTERO-30.mol	[H]O[C@@]1([H])C([H])=C2C([H])([H])C([H])([H])[C@@]3([H])[C@]4([H])C([H])([H])C([H])([H])[C@]([H])(C(=O)/C([H])=C(\[H])c5c(OC([H])([H])C([H])([H])[H])nc6c([H])c(SC([H])([H])[H])c([H])c([H])c6c5[H])[C@@]4(C([H])([H])[H])C([H])([H])C([H])([H])[C@]3([H])[C@@]2(C([H])([H])[H])C([H])([H])C1([H])[H]
ESTERO-31.mol	[H]O[C@@]1([H])C([H])=C2C([H])([H])C([H])([H])[C@@]3([H])[C@]4([H])C([H])([H])C([H])([H])[C@]([H])(C(=O)/C([H])=C(\[H])c5c(OC([H])([H])C([H])([H])[H])nc6c([H])c([H])c(Cl)c([H])c6c5[H])[C@@]4(C([H])([H])[H])C([H])([H])C([H])([H])[C@]3([H])[C@@]2(C([H])([H])[H])C([H])([H])C1([H])[H]
ESTERO-32.mol	[H]O[C@@]1([H])C([H])=C2C([H])([H])C([H])([H])[C@@]3([H])[C@]4([H])C([H])([H])C([H])([H])[C@]([H])(C(=O)/C([H])=C(\[H])c5c(OC([H])([H])C([H])([H])[H])nc6c([H])c([H])c(OC([H])([H])[H])c([H])c6c5[H])[C@@]4(C([H])([H])[H])C([H])([H])C([H])([H])[C@]3([H])[C@@]2(C([H])([H])[H])C([H])([H])C1([H])[H]

ESTERO-33.mol	[H]O[C@@]1([H])C([H])=C2C([H])([H])C([H])([H])[C@@]3([H])[C@]4([H])C([H])([H])C([H])([H])[C@]([H])(C(=O)/C([H])=C(\[H])c5c(OC([H])([H])C([H])([H])[H])nc6c([H])c([H])c(C(F)(F)F)c([H])c6c5[H])[C@@]4(C([H])([H])[H])C([H])([H])C([H])([H])[C@]3([H])[C@@]2(C([H])([H])[H])C([H])([H])C1([H])[H]
ESTERO-34.mol	[H]O[C@@]1([H])C([H])=C2C([H])([H])C([H])([H])[C@@]3([H])[C@]4([H])C([H])([H])C([H])([H])[C@]([H])(C(=O)/C([H])=C(\[H])c5c(OC([H])([H])C([H])([H])[H])nc6c([H])c([H])c(C([H])([H])[H])c([H])c6c5[H])[C@@]4(C([H])([H])[H])C([H])([H])C([H])([H])[C@]3([H])[C@@]2(C([H])([H])[H])C([H])([H])C1([H])[H]
ESTERO-35.mol	[H]O[C@@]1([H])C([H])=C2C([H])([H])C([H])([H])[C@@]3([H])[C@]4([H])C([H])([H])C([H])([H])[C@]([H])(C(=O)/C([H])=C(\[H])c5c(OC([H])([H])C([H])([H])[H])nc6c([H])c([H])c([H])c([H])c6c5[H])[C@@]4(C([H])([H])[H])C([H])([H])C([H])([H])[C@]3([H])[C@@]2(C([H])([H])[H])C([H])([H])C1([H])[H]
ESTERO-36.mol	[H]O[C@@]1([H])C([H])=C2C([H])([H])C([H])([H])[C@@]3([H])[C@]4([H])C([H])([H])C([H])([H])[C@]([H])(C(=O)/C([H])=C(\[H])c5c(OC([H])([H])C([H])([H])[H])nc6c([H])c([H])c(SC([H])([H])[H])c([H])c6c5[H])[C@@]4(C([H])([H])[H])C([H])([H])C([H])([H])[C@]3([H])[C@@]2(C([H])([H])[H])C([H])([H])C1([H])[H]
ESTERO-37.mol	[H]O[C@@]1([H])C([H])=C2C([H])([H])C([H])([H])[C@@]3([H])[C@]4([H])C([H])([H])C([H])([H])[C@]([H])(C5=NN(C(=O)C([H])([H])[H])[C@@]([H])(C6=C([H])[C@]7([H])C([H])=C([H])C(Cl)=C([H])[C@]7([H])N([H])C6=O)C5([H])[H])[C@@]4(C([H])([H])[H])C([H])([H])C([H])([H])[C@]3([H])[C@@]2(C([H])([H])[H])C([H])([H])C1([H])[H]
ESTERO-38.mol	[H]O[C@@]1([H])C([H])=C2C([H])([H])C([H])([H])[C@@]3([H])[C@]4([H])C([H])([H])C([H])([H])[C@]([H])(C5=NN(C(=O)C([H])([H])[H])[C@@]([H])(C6=C([H])[C@]7([H])C([H])=C([H])C(OC([H])([H])[H])=C([H])[C@]7([H])N([H])C6=O)C5([H])[H])[C@@]4(C([H])([H])[H])C([H])([H])C([H])([H])[C@]3([H])[C@@]2(C([H])([H])[H])C([H])([H])C1([H])[H]
ESTERO-39.mol	[H]O[C@@]1([H])C([H])=C2C([H])([H])C([H])([H])[C@@]3([H])[C@]4([H])C([H])([H])C([H])([H])[C@]([H])(C5=NN(C(=O)C([H])([H])[H])[C@@]([H])(C6=C([H])[C@]7([H])C([H])=C([H])C(C(F)(F)F)=C([H])[C@]7([H])N([H])C6=O)C5([H])[H])[C@@]4(C([H])([H])[H])C([H])([H])C([H])([H])[C@]3([H])[C@@]2(C([H])([H])[H])C([H])([H])C1([H])[H]
ESTERO-40.mol	[H]O[C@@]1([H])C([H])=C2C([H])([H])C([H])([H])[C@@]3([H])[C@]4([H])C([H])([H])C([H])([H])[C@]([H])(C5=NN(C(=O)C([H])([H])[H])[C@@]([H])(C6=C([H])[C@]7([H])C([H])=C([H])C(C([H])([H])[H])=C([H])[C@]7([H])N([H])C6=O)C5([H])[H])[C@@]4(C([H])([H])[H])C([H])([H])C([H])([H])[C@]3([H])[C@@]2(C([H])([H])[H])C([H])([H])C1([H])[H]
ESTERO-41.mol	[H]O[C@@]1([H])C([H])=C2C([H])([H])C([H])([H])[C@@]3([H])[C@]4([H])C([H])([H])C([H])([H])[C@]([H])(C5=NN(C(=O)C([H])([H])[H])[C@@]([H])(C6=C([H])[C@]7([H])C([H])=C([H])C([H])=C([H])[C@]7([H])N([H])C6=O)C5([H])[H])[C@@]4(C([H])([H])[H])C([H])([H])C([H])([H])[C@]3([H])[C@@]2(C([H])([H])[H])C([H])([H])C1([H])[H]
ESTERO-42.mol	[H]O[C@@]1([H])C([H])=C2C([H])([H])C([H])([H])[C@@]3([H])[C@]4([H])C([H])([H])C([H])([H])[C@]([H])(C5=NN(C(=O)C([H])([H])[H])[C@@]([H])(C6=C([H])[C@]7([H])C([H])=C([H])C(SC([H])([H])[H])=C([H])[C@]7([H])N([H])C6=O)C5([H])[H])[C@@]4(C([H])([H])[H])C([H])([H])C([H])([H])[C@]3([H])[C@@]2(C([H])([H])[H])C([H])([H])C1([H])[H]
ESTERO-43.mol	[H]O[C@@]1([H])C([H])=C2C([H])([H])C([H])([H])[C@@]3([H])[C@]4([H])C([H])([H])C([H])([H])[C@]([H])(C5=NN(C(=O)C([H])([H])[H])[C@@]([H])(C6=C([H])[C@]7([H])C([H])=C([H])C(Br)=C([H])[C@]7([H])N([H])C6=O)C5([H])[H])[C@@]4(C([H])([H])[H])C([H])([H])C([H])([H])[C@]3([H])[C@@]2(C([H])([H])[H])C([H])([H])C1([H])[H]
ESTERO-44.mol	[H]O[C@@]1([H])C([H])=C2C([H])([H])C([H])([H])[C@@]3([H])[C@]4([H])C([H])([H])C([H])([H])[C@]([H])(C5=NN(C(=O)C([H])([H])[H])[C@@]([H])(C6=C([H])[C@]7([H])C([H])=C(Cl)C([H])=C([H])[C@]7([H])N([H])C6=O)C5([H])[H])[C@@]4(C([H])([H])[H])C([H])([H])C([H])([H])[C@]3([H])[C@@]2(C([H])([H])[H])C([H])([H])C1([H])[H]

ESTERO-45.mol	[H]O[C@@]1([H])C([H])=C2C([H])([H])C([H])([H])[C@@]3([H])[C@]4([H])C([H])([H])C([H])([H])[C@]([H])(C5=NN(C(=O)C([H])([H])[H])[C@@]([H])(C6=C([H])[C@]7([H])C([H])=C(OC([H])([H])[H])C([H])=C([H])[C@]7([H])N([H])C6=O)C5([H])[H])[C@@]4(C([H])([H])[H])C([H])([H])C([H])([H])[C@]3([H])[C@@]2(C([H])([H])[H])C([H])([H])C1([H])[H]
ESTERO-46.mol	[H]O[C@@]1([H])C([H])=C2C([H])([H])C([H])([H])[C@@]3([H])[C@]4([H])C([H])([H])C([H])([H])[C@]([H])(C5=NN(C(=O)C([H])([H])[H])[C@@]([H])(C6=C([H])[C@]7([H])C([H])=C(C(F)(F)F)C([H])=C([H])[C@]7([H])N([H])C6=O)C5([H])[H])[C@@]4(C([H])([H])[H])C([H])([H])C([H])([H])[C@]3([H])[C@@]2(C([H])([H])[H])C([H])([H])C1([H])[H]
ESTERO-47.mol	[H]O[C@@]1([H])C([H])=C2C([H])([H])C([H])([H])[C@@]3([H])[C@]4([H])C([H])([H])C([H])([H])[C@]([H])(C5=NN(C(=O)C([H])([H])[H])[C@@]([H])(C6=C([H])[C@]7([H])C([H])=C(C([H])([H])[H])C([H])=C([H])[C@]7([H])N([H])C6=O)C5([H])[H])[C@@]4(C([H])([H])[H])C([H])([H])C([H])([H])[C@]3([H])[C@@]2(C([H])([H])[H])C([H])([H])C1([H])[H]
ESTERO-48.mol	[H]O[C@@]1([H])C([H])=C2C([H])([H])C([H])([H])[C@@]3([H])[C@]4([H])C([H])([H])C([H])([H])[C@]([H])(C5=NN(C(=O)C([H])([H])[H])[C@@]([H])(C6=C([H])[C@]7([H])C([H])=C([H])C([H])=C([H])[C@]7([H])N([H])C6=O)C5([H])[H])[C@@]4(C([H])([H])[H])C([H])([H])C([H])([H])[C@]3([H])[C@@]2(C([H])([H])[H])C([H])([H])C1([H])[H]
ESTERO-49.mol	[H]O[C@@]1([H])C([H])=C2C([H])([H])C([H])([H])[C@@]3([H])[C@]4([H])C([H])([H])C([H])([H])[C@]([H])(C5=NN(C(=O)C([H])([H])[H])[C@@]([H])(C6=C([H])[C@]7([H])C([H])=C(SC([H])([H])[H])C([H])=C([H])[C@]7([H])N([H])C6=O)C5([H])[H])[C@@]4(C([H])([H])[H])C([H])([H])C([H])([H])[C@]3([H])[C@@]2(C([H])([H])[H])C([H])([H])C1([H])[H]
ESTERO-50.mol	[H]O[C@@]1([H])C([H])=C2C([H])([H])C([H])([H])[C@@]3([H])[C@]4([H])C([H])([H])C([H])([H])[C@]([H])(C5=NN(C(=O)C([H])([H])[H])[C@@]([H])(C6=C([H])[C@]7([H])C([H])=C(Br)C([H])=C([H])[C@]7([H])N([H])C6=O)C5([H])[H])[C@@]4(C([H])([H])[H])C([H])([H])C([H])([H])[C@]3([H])[C@@]2(C([H])([H])[H])C([H])([H])C1([H])[H]
Estero_51.mol	[H]O[C@@]1([H])C([H])([H])C([H])([H])[C@@]2([H])[C@]3([H])C([H])([H])C([H])([H])C4=C([H])[C@]([H])(/C([H])=C(\[H])c5c(Cl)nc6c([H])c(OC([H])([H])[H])c([H])c([H])c6c5[H])C([H])([H])C([H])([H])[C@]4(C([H])([H])[H])[C@@]3([H])C([H])([H])C([H])([H])[C@@]21C([H])([H])[H]
Estero_52.mol	[H]O[C@@]1([H])C([H])([H])C([H])([H])[C@@]2([H])[C@]3([H])C([H])([H])C([H])([H])C4=C([H])[C@]([H])(/C([H])=C(\[H])c5c(Cl)nc6c([H])c(Cl)c([H])c([H])c6c5[H])C([H])([H])C([H])([H])[C@]4(C([H])([H])[H])[C@@]3([H])C([H])([H])C([H])([H])[C@@]21C([H])([H])[H]
Estero_53.mol	[H]O[C@@]1([H])C([H])([H])C([H])([H])[C@@]2([H])[C@]3([H])C([H])([H])C([H])([H])C4=C([H])[C@]([H])(/C([H])=C(\[H])c5c(Cl)nc6c([H])c(OC([H])([H])[H])c([H])c([H])c6c5[H])C([H])([H])C([H])([H])[C@]4(C([H])([H])[H])[C@@]3([H])C([H])([H])C([H])([H])[C@@]21C([H])([H])[H]
Estero_54.mol	[H]O[C@@]1([H])C([H])([H])C([H])([H])[C@@]2([H])[C@]3([H])C([H])([H])C([H])([H])C4=C([H])[C@]([H])(/C([H])=C(\[H])c5c(Cl)nc6c([H])c(C(F)(F)F)c([H])c([H])c6c5[H])C([H])([H])C([H])([H])[C@]4(C([H])([H])[H])[C@@]3([H])C([H])([H])C([H])([H])[C@@]21C([H])([H])[H]
Estero_55.mol	[H]O[C@@]1([H])C([H])([H])C([H])([H])[C@@]2([H])[C@]3([H])C([H])([H])C([H])([H])C4=C([H])[C@]([H])(/C([H])=C(\[H])c5c(Cl)nc6c([H])c(C([H])([H])[H])c([H])c([H])c6c5[H])C([H])([H])C([H])([H])[C@]4(C([H])([H])[H])[C@@]3([H])C([H])([H])C([H])([H])[C@@]21C([H])([H])[H]
Estero_56.mol	[H]O[C@@]1([H])C([H])([H])C([H])([H])[C@@]2([H])[C@]3([H])C([H])([H])C([H])([H])C4=C([H])[C@]([H])(/C([H])=C(\[H])c5c(Cl)nc6c([H])c([H])c([H])c([H])c6c5[H])C([H])([H])C([H])([H])[C@]4(C([H])([H])[H])[C@@]3([H])C([H])([H])C([H])([H])[C@@]21C([H])([H])[H]

Estero_57.mol	[H]O[C@@]1([H])C([H])([H])C([H])([H])[C@@]2([H])[C@]3([H])C([H])([H])C([H])([H])C4=C([H])[C@]([H])(/C([H])=C(\[H])c5c(Cl)nc6c([H])c(SC([H])([H])[H])c([H])c([H])c6c5[H])C([H])([H])C([H])([H])[C@]4(C([H])([H])[H])[C@@]3([H])C([H])([H])C([H])([H])[C@@]21C([H])([H])[H]
Estero_58.mol	[H]O[C@@]1([H])C([H])([H])C([H])([H])[C@@]2([H])[C@]3([H])C([H])([H])C([H])([H])C4=C([H])[C@]([H])(/C([H])=C(\[H])c5c(Cl)nc6c([H])c([H])c(Cl)c([H])c6c5[H])C([H])([H])C([H])([H])[C@]4(C([H])([H])[H])[C@@]3([H])C([H])([H])C([H])([H])[C@@]21C([H])([H])[H]
Estero_59.mol	[H]O[C@@]1([H])C([H])([H])C([H])([H])[C@@]2([H])[C@]3([H])C([H])([H])C([H])([H])C4=C([H])[C@]([H])(/C([H])=C(\[H])c5c(Cl)nc6c([H])c([H])c(OC([H])([H])[H])c([H])c6c5[H])C([H])([H])C([H])([H])[C@]4(C([H])([H])[H])[C@@]3([H])C([H])([H])C([H])([H])[C@@]21C([H])([H])[H]
Estero_60.mol	[H]O[C@@]1([H])C([H])([H])C([H])([H])[C@@]2([H])[C@]3([H])C([H])([H])C([H])([H])C4=C([H])[C@]([H])(/C([H])=C(\[H])c5c(Cl)nc6c([H])c([H])c(C(F)(F)F)c([H])c6c5[H])C([H])([H])C([H])([H])[C@]4(C([H])([H])[H])[C@@]3([H])C([H])([H])C([H])([H])[C@@]21C([H])([H])[H]
Estero_61.mol	[H]O[C@@]1([H])C([H])([H])C([H])([H])[C@@]2([H])[C@]3([H])C([H])([H])C([H])([H])C4=C([H])[C@]([H])(/C([H])=C(\[H])c5c(Cl)nc6c([H])c([H])c(C([H])([H])[H])c([H])c6c5[H])C([H])([H])C([H])([H])[C@]4(C([H])([H])[H])[C@@]3([H])C([H])([H])C([H])([H])[C@@]21C([H])([H])[H]
Estero_62.mol	[H]O[C@@]1([H])C([H])([H])C([H])([H])[C@@]2([H])[C@]3([H])C([H])([H])C([H])([H])C4=C([H])[C@]([H])(/C([H])=C(\[H])c5c(Cl)nc6c([H])c([H])c([H])c([H])c6c5[H])C([H])([H])C([H])([H])[C@]4(C([H])([H])[H])[C@@]3([H])C([H])([H])C([H])([H])[C@@]21C([H])([H])[H]
Estero_63.mol	[H]O[C@@]1([H])C([H])([H])C([H])([H])[C@@]2([H])[C@]3([H])C([H])([H])C([H])([H])C4=C([H])[C@]([H])(/C([H])=C(\[H])c5c(Cl)nc6c([H])c([H])c(SC([H])([H])[H])c([H])c6c5[H])C([H])([H])C([H])([H])[C@]4(C([H])([H])[H])[C@@]3([H])C([H])([H])C([H])([H])[C@@]21C([H])([H])[H]
Estero_64.mol	[H]O[C@@]1([H])C([H])([H])C([H])([H])[C@@]2([H])[C@]3([H])C([H])([H])C([H])([H])C4=C([H])[C@]([H])(/C([H])=C(\[H])c5c(Cl)nc6c([H])c(Cl)c(Cl)c([H])c6c5[H])C([H])([H])C([H])([H])[C@]4(C([H])([H])[H])[C@@]3([H])C([H])([H])C([H])([H])[C@@]21C([H])([H])[H]
Estero_65.mol	[H]O[C@@]1([H])C([H])([H])C([H])([H])[C@@]2([H])[C@]3([H])C([H])([H])C([H])([H])C4=C([H])[C@]([H])(/C([H])=C(\[H])c5c(Cl)nc6c([H])c(Cl)c(OC([H])([H])[H])c([H])c6c5[H])C([H])([H])C([H])([H])[C@]4(C([H])([H])[H])[C@@]3([H])C([H])([H])C([H])([H])[C@@]21C([H])([H])[H]
Estero_66.mol	[H]O[C@@]1([H])C([H])([H])C([H])([H])[C@@]2([H])[C@]3([H])C([H])([H])C([H])([H])C4=C([H])[C@]([H])(/C([H])=C(\[H])c5c(Cl)nc6c([H])c(Cl)c(C(F)(F)F)c([H])c6c5[H])C([H])([H])C([H])([H])[C@]4(C([H])([H])[H])[C@@]3([H])C([H])([H])C([H])([H])[C@@]21C([H])([H])[H]
Estero_67.mol	[H]O[C@@]1([H])C([H])([H])C([H])([H])[C@@]2([H])[C@]3([H])C([H])([H])C([H])([H])C4=C([H])[C@]([H])(/C([H])=C(\[H])c5c(Cl)nc6c([H])c(Cl)c(C([H])([H])[H])c([H])c6c5[H])C([H])([H])C([H])([H])[C@]4(C([H])([H])[H])[C@@]3([H])C([H])([H])C([H])([H])[C@@]21C([H])([H])[H]
Estero_68.mol	[H]O[C@@]1([H])C([H])([H])C([H])([H])[C@@]2([H])[C@]3([H])C([H])([H])C([H])([H])C4=C([H])[C@]([H])(/C([H])=C(\[H])c5c(Cl)nc6c([H])c(Cl)c([H])c([H])c6c5[H])C([H])([H])C([H])([H])[C@]4(C([H])([H])[H])[C@@]3([H])C([H])([H])C([H])([H])[C@@]21C([H])([H])[H]
Estero_69.mol	[H]O[C@@]1([H])C([H])([H])C([H])([H])[C@@]2([H])[C@]3([H])C([H])([H])C([H])([H])C4=C([H])[C@]([H])(/C([H])=C(\[H])c5c(Cl)nc6c([H])c(Cl)c(SC([H])([H])[H])c([H])c6c5[H])C([H])([H])C([H])([H])[C@]4(C([H])([H])[H])[C@@]3([H])C([H])([H])C([H])([H])[C@@]21C([H])([H])[H]

Estero_70.mol	[H]O[C@@]1([H])C([H])([H])C([H])([H])[C@@]2([H])[C@]3([H])C([H])([H])C([H])([H])C4=C([H])[C@]([H])(/C([H])=C(\[H])c5c(Cl)nc6c([H])c(OC([H])([H])[H])c(Cl)c([H])c6c5[H])C([H])([H])C([H])([H])[C@]4(C([H])([H])[H])[C@@]3([H])C([H])([H])C([H])([H])[C@@]21C([H])([H])[H]
Estero_71.mol	[H]O[C@@]1([H])C([H])([H])C([H])([H])[C@@]2([H])[C@]3([H])C([H])([H])C([H])([H])C4=C([H])[C@]([H])(/C([H])=C(\[H])c5c(Cl)nc6c([H])c(OC([H])([H])[H])c(OC([H])([H])[H])c([H])c6c5[H])C([H])([H])C([H])([H])[C@]4(C([H])([H])[H])[C@@]3([H])C([H])([H])C([H])([H])[C@@]21C([H])([H])[H]
Estero_72.mol	[H]O[C@@]1([H])C([H])([H])C([H])([H])[C@@]2([H])[C@]3([H])C([H])([H])C([H])([H])C4=C([H])[C@]([H])(/C([H])=C(\[H])c5c(Cl)nc6c([H])c(OC([H])([H])[H])c(C(F)(F)F)c([H])c6c5[H])C([H])([H])C([H])([H])[C@]4(C([H])([H])[H])[C@@]3([H])C([H])([H])C([H])([H])[C@@]21C([H])([H])[H]
Estero_73.mol	[H]O[C@@]1([H])C([H])([H])C([H])([H])[C@@]2([H])[C@]3([H])C([H])([H])C([H])([H])C4=C([H])[C@]([H])(/C([H])=C(\[H])c5c(Cl)nc6c([H])c(OC([H])([H])[H])c(C([H])([H])[H])c([H])c6c5[H])C([H])([H])C([H])([H])[C@]4(C([H])([H])[H])[C@@]3([H])C([H])([H])C([H])([H])[C@@]21C([H])([H])[H]
Estero_74.mol	[H]O[C@@]1([H])C([H])([H])C([H])([H])[C@@]2([H])[C@]3([H])C([H])([H])C([H])([H])C4=C([H])[C@]([H])(/C([H])=C(\[H])c5c(Cl)nc6c([H])c(OC([H])([H])[H])c([H])c([H])c6c5[H])C([H])([H])C([H])([H])[C@]4(C([H])([H])[H])[C@@]3([H])C([H])([H])C([H])([H])[C@@]21C([H])([H])[H]
Estero_76.mol	[H]O[C@@]1([H])C([H])([H])C([H])([H])[C@@]2([H])[C@]3([H])C([H])([H])C([H])([H])C4=C([H])[C@]([H])(/C([H])=C(\[H])c5c(Cl)nc6c([H])c(C(F)(F)F)c(Cl)c([H])c6c5[H])C([H])([H])C([H])([H])[C@]4(C([H])([H])[H])[C@@]3([H])C([H])([H])C([H])([H])[C@@]21C([H])([H])[H]
Estero_77.mol	[H]O[C@@]1([H])C([H])([H])C([H])([H])[C@@]2([H])[C@]3([H])C([H])([H])C([H])([H])C4=C([H])[C@]([H])(/C([H])=C(\[H])c5c(Cl)nc6c([H])c(C(F)(F)F)c(OC([H])([H])[H])c([H])c6c5[H])C([H])([H])C([H])([H])[C@]4(C([H])([H])[H])[C@@]3([H])C([H])([H])C([H])([H])[C@@]21C([H])([H])[H]
Estero_78.mol	[H]O[C@@]1([H])C([H])([H])C([H])([H])[C@@]2([H])[C@]3([H])C([H])([H])C([H])([H])C4=C([H])[C@]([H])(/C([H])=C(\[H])c5c(Cl)nc6c([H])c(C(F)(F)F)c(C(F)(F)F)c([H])c6c5[H])C([H])([H])C([H])([H])[C@]4(C([H])([H])[H])[C@@]3([H])C([H])([H])C([H])([H])[C@@]21C([H])([H])[H]
Estero_79.mol	[H]O[C@@]1([H])C([H])([H])C([H])([H])[C@@]2([H])[C@]3([H])C([H])([H])C([H])([H])C4=C([H])[C@]([H])(/C([H])=C(\[H])c5c(Cl)nc6c([H])c(C(F)(F)F)c(C([H])([H])[H])c([H])c6c5[H])C([H])([H])C([H])([H])[C@]4(C([H])([H])[H])[C@@]3([H])C([H])([H])C([H])([H])[C@@]21C([H])([H])[H]
Estero_81.mol	[H]O[C@@]1([H])C([H])([H])C([H])([H])[C@@]2([H])[C@]3([H])C([H])([H])C([H])([H])C4=C([H])[C@]([H])(/C([H])=C(\[H])c5c(Cl)nc6c([H])c(C(F)(F)F)c(SC([H])([H])[H])c([H])c6c5[H])C([H])([H])C([H])([H])[C@]4(C([H])([H])[H])[C@@]3([H])C([H])([H])C([H])([H])[C@@]21C([H])([H])[H]
Estero_82.mol	[H]O[C@@]1([H])C([H])([H])C([H])([H])[C@@]2([H])[C@]3([H])C([H])([H])C([H])([H])C4=C([H])[C@]([H])(/C([H])=C(\[H])c5c(Cl)nc6c([H])c(C([H])([H])[H])c(Cl)c([H])c6c5[H])C([H])([H])C([H])([H])[C@]4(C([H])([H])[H])[C@@]3([H])C([H])([H])C([H])([H])[C@@]21C([H])([H])[H]
Estero_83.mol	[H]O[C@@]1([H])C([H])([H])C([H])([H])[C@@]2([H])[C@]3([H])C([H])([H])C([H])([H])C4=C([H])[C@]([H])(/C([H])=C(\[H])c5c(Cl)nc6c([H])c(C([H])([H])[H])c(C(F)(F)F)c([H])c6c5[H])C([H])([H])C([H])([H])[C@]4(C([H])([H])[H])[C@@]3([H])C([H])([H])C([H])([H])[C@@]21C([H])([H])[H]
Estero_84.mol	[H]O[C@@]1([H])C([H])([H])C([H])([H])[C@@]2([H])[C@]3([H])C([H])([H])C([H])([H])C4=C([H])[C@]([H])(/C([H])=C(\[H])c5c(Cl)nc6c([H])c(C([H])([H])[H])c(C([H])([H])[H])c([H])c6c5[H])C([H])([H])C([H])([H])[C@]4(C([H])([H])[H])[C@@]3([H])C([H])([H])C([H])([H])[C@@]21C([H])([H])[H]

Estero_85.mol	[H]O[C@@]1([H])C([H])([H])C([H])([H])[C@@]2([H])[C@]3([H])C([H])([H])C([H])([H])C4=C([H])[C@]([H])(/C([H])=C(\[H])c5c(Cl)nc6c([H])c(C([H])([H])[H])c([H])c([H])c6c5[H])C([H])([H])C([H])([H])[C@]4(C([H])([H])[H])[C@@]3([H])C([H])([H])C([H])([H])[C@@]21C([H])([H])[H]
Estero_86.mol	[H]O[C@@]1([H])C([H])([H])C([H])([H])[C@@]2([H])[C@]3([H])C([H])([H])C([H])([H])C4=C([H])[C@]([H])(/C([H])=C(\[H])c5c(Cl)nc6c([H])c(C([H])([H])[H])c(SC([H])([H])[H])c([H])c6c5[H])C([H])([H])C([H])([H])[C@]4(C([H])([H])[H])[C@@]3([H])C([H])([H])C([H])([H])[C@@]21C([H])([H])[H]
Estero_87.mol	[H]O[C@@]1([H])C([H])([H])C([H])([H])[C@@]2([H])[C@]3([H])C([H])([H])C([H])([H])C4=C([H])[C@]([H])(/C([H])=C(\[H])c5c(Cl)nc6c([H])c([H])c(Cl)c([H])c6c5[H])C([H])([H])C([H])([H])[C@]4(C([H])([H])[H])[C@@]3([H])C([H])([H])C([H])([H])[C@@]21C([H])([H])[H]
Estero_88.mol	[H]O[C@@]1([H])C([H])([H])C([H])([H])[C@@]2([H])[C@]3([H])C([H])([H])C([H])([H])C4=C([H])[C@]([H])(/C([H])=C(\[H])c5c(Cl)nc6c([H])c([H])c(OC([H])([H])[H])c([H])c6c5[H])C([H])([H])C([H])([H])[C@]4(C([H])([H])[H])[C@@]3([H])C([H])([H])C([H])([H])[C@@]21C([H])([H])[H]
Estero_89.mol	[H]O[C@@]1([H])C([H])([H])C([H])([H])[C@@]2([H])[C@]3([H])C([H])([H])C([H])([H])C4=C([H])[C@]([H])(/C([H])=C(\[H])c5c(Cl)nc6c([H])c([H])c(C(F)(F)F)c([H])c6c5[H])C([H])([H])C([H])([H])[C@]4(C([H])([H])[H])[C@@]3([H])C([H])([H])C([H])([H])[C@@]21C([H])([H])[H]
Estero_90.mol	[H]O[C@@]1([H])C([H])([H])C([H])([H])[C@@]2([H])[C@]3([H])C([H])([H])C([H])([H])C4=C([H])[C@]([H])(/C([H])=C(\[H])c5c(Cl)nc6c([H])c([H])c(C([H])([H])[H])c([H])c6c5[H])C([H])([H])C([H])([H])[C@]4(C([H])([H])[H])[C@@]3([H])C([H])([H])C([H])([H])[C@@]21C([H])([H])[H]
Estero_91.mol	[H]O[C@@]1([H])C([H])([H])C([H])([H])[C@@]2([H])[C@]3([H])C([H])([H])C([H])([H])C4=C([H])[C@]([H])(/C([H])=C(\[H])c5c(Cl)nc6c([H])c([H])c([H])c([H])c6c5[H])C([H])([H])C([H])([H])[C@]4(C([H])([H])[H])[C@@]3([H])C([H])([H])C([H])([H])[C@@]21C([H])([H])[H]
Estero_92.mol	[H]O[C@@]1([H])C([H])([H])C([H])([H])[C@@]2([H])[C@]3([H])C([H])([H])C([H])([H])C4=C([H])[C@]([H])(/C([H])=C(\[H])c5c(Cl)nc6c([H])c([H])c(SC([H])([H])[H])c([H])c6c5[H])C([H])([H])C([H])([H])[C@]4(C([H])([H])[H])[C@@]3([H])C([H])([H])C([H])([H])[C@@]21C([H])([H])[H]
Estero_93.mol	[H]O[C@@]1([H])C([H])([H])C([H])([H])[C@@]2([H])[C@]3([H])C([H])([H])C([H])([H])C4=C([H])[C@]([H])(/C([H])=C(\[H])c5c(Cl)nc6c([H])c(SC([H])([H])[H])c(SC([H])([H])[H])c([H])c6c5[H])C([H])([H])C([H])([H])[C@]4(C([H])([H])[H])[C@@]3([H])C([H])([H])C([H])([H])[C@@]21C([H])([H])[H]
Estero_94.mol	[H]O[C@@]1([H])C([H])([H])C([H])([H])[C@@]2([H])[C@]3([H])C([H])([H])C([H])([H])C4=C([H])[C@]([H])(/C([H])=C(\[H])c5c(Cl)nc6c([H])c(SC([H])([H])[H])c([H])c([H])c6c5[H])C([H])([H])C([H])([H])[C@]4(C([H])([H])[H])[C@@]3([H])C([H])([H])C([H])([H])[C@@]21C([H])([H])[H]
Estero_95.mol	[H]O[C@@]1([H])C([H])([H])C([H])([H])[C@@]2([H])[C@]3([H])C([H])([H])C([H])([H])C4=C([H])[C@]([H])(/C([H])=C(\[H])c5c(Cl)nc6c([H])c(SC([H])([H])[H])c(C([H])([H])[H])c([H])c6c5[H])C([H])([H])C([H])([H])[C@]4(C([H])([H])[H])[C@@]3([H])C([H])([H])C([H])([H])[C@@]21C([H])([H])[H]
Estero_96.mol	[H]O[C@@]1([H])C([H])([H])C([H])([H])[C@@]2([H])[C@]3([H])C([H])([H])C([H])([H])C4=C([H])[C@]([H])(/C([H])=C(\[H])c5c(Cl)nc6c([H])c(SC([H])([H])[H])c(C(F)(F)F)c([H])c6c5[H])C([H])([H])C([H])([H])[C@]4(C([H])([H])[H])[C@@]3([H])C([H])([H])C([H])([H])[C@@]21C([H])([H])[H]
Estero_97.mol	[H]O[C@@]1([H])C([H])([H])C([H])([H])[C@@]2([H])[C@]3([H])C([H])([H])C([H])([H])C4=C([H])[C@]([H])(/C([H])=C(\[H])c5c(Cl)nc6c([H])c(SC([H])([H])[H])c(OC([H])([H])[H])c([H])c6c5[H])C([H])([H])C([H])([H])[C@]4(C([H])([H])[H])[C@@]3([H])C([H])([H])C([H])([H])[C@@]21C([H])([H])[H]

Estero_98.mol	[H]O[C@@]1([H])C([H])([H])C([H])([H])[C@@]2([H])[C@]3([H])C([H])([H])C([H])([H])C4=C([H])[C@]([H])(/C([H])=C(\[H])c5c(Cl)nc6c([H])c(SC([H])([H])[H])c(Cl)c([H])c6c5[H])C([H])([H])C([H])([H])[C@]4(C([H])([H])[H])[C@@]3([H])C([H])([H])C([H])([H])[C@@]21C([H])([H])[H]
Estero_99.mol	[H]O[C@@]1([H])C([H])([H])C([H])([H])[C@@]2([H])[C@]3([H])C([H])([H])C([H])([H])C4=C([H])[C@]([H])(/C([H])=C(\[H])c5c(Cl)nc6c(Cl)c([H])c([H])c([H])c6c5[H])C([H])([H])C([H])([H])[C@]4(C([H])([H])[H])[C@@]3([H])C([H])([H])C([H])([H])[C@@]21C([H])([H])[H]
Estero_101.mol	[H]O[C@@]1([H])C([H])([H])C([H])([H])[C@@]2([H])[C@]3([H])C([H])([H])C([H])([H])C4=C([H])[C@]([H])(/C([H])=C(\[H])c5c(Cl)nc6c(C(F)(F)F)c([H])c([H])c([H])c6c5[H])C([H])([H])C([H])([H])[C@]4(C([H])([H])[H])[C@@]3([H])C([H])([H])C([H])([H])[C@@]21C([H])([H])[H]
Estero_102.mol	[H]O[C@@]1([H])C([H])([H])C([H])([H])[C@@]2([H])[C@]3([H])C([H])([H])C([H])([H])C4=C([H])[C@]([H])(/C([H])=C(\[H])c5c(Cl)nc6c(C([H])([H])[H])c([H])c([H])c([H])c6c5[H])C([H])([H])C([H])([H])[C@]4(C([H])([H])[H])[C@@]3([H])C([H])([H])C([H])([H])[C@@]21C([H])([H])[H]
Estero_103.mol	[H]O[C@@]1([H])C([H])([H])C([H])([H])[C@@]2([H])[C@]3([H])C([H])([H])C([H])([H])C4=C([H])[C@]([H])(/C([H])=C(\[H])c5c(Cl)nc6c([H])c([H])c([H])c([H])c6c5[H])C([H])([H])C([H])([H])[C@]4(C([H])([H])[H])[C@@]3([H])C([H])([H])C([H])([H])[C@@]21C([H])([H])[H]
Estero_104.mol	[H]O[C@@]1([H])C([H])([H])C([H])([H])[C@@]2([H])[C@]3([H])C([H])([H])C([H])([H])C4=C([H])[C@]([H])(/C([H])=C(\[H])c5c(Cl)nc6c(SC([H])([H])[H])c([H])c([H])c([H])c6c5[H])C([H])([H])C([H])([H])[C@]4(C([H])([H])[H])[C@@]3([H])C([H])([H])C([H])([H])[C@@]21C([H])([H])[H]
Estero_105.mol	[H]O[C@@]1([H])C([H])([H])C([H])([H])[C@@]2([H])[C@]3([H])C([H])([H])C([H])([H])C4=C([H])[C@]([H])(/C([H])=C(\[H])[C@]5([H])C(=O)N([H])[C@@]6([H])C([H])=C([H])C(Cl)=C([H])C6=C5[H])C([H])([H])C([H])([H])[C@]4(C([H])([H])[H])[C@@]3([H])C([H])([H])C([H])([H])[C@@]21C([H])([H])[H]
Estero_106.mol	[H]O[C@@]1([H])C([H])([H])C([H])([H])[C@@]2([H])[C@]3([H])C([H])([H])C([H])([H])C4=C([H])[C@]([H])(/C([H])=C(\[H])[C@]5([H])C(=O)N([H])[C@@]6([H])C([H])=C([H])C(OC([H])([H])[H])=C([H])C6=C5[H])C([H])([H])C([H])([H])[C@]4(C([H])([H])[H])[C@@]3([H])C([H])([H])C([H])([H])[C@@]21C([H])([H])[H]
Estero_107.mol	[H]O[C@@]1([H])C([H])([H])C([H])([H])[C@@]2([H])[C@]3([H])C([H])([H])C([H])([H])C4=C([H])[C@]([H])(/C([H])=C(\[H])[C@]5([H])C(=O)N([H])[C@@]6([H])C([H])=C([H])C(C(F)(F)F)=C([H])C6=C5[H])C([H])([H])C([H])([H])[C@]4(C([H])([H])[H])[C@@]3([H])C([H])([H])C([H])([H])[C@@]21C([H])([H])[H]
Estero_108.mol	[H]O[C@@]1([H])C([H])([H])C([H])([H])[C@@]2([H])[C@]3([H])C([H])([H])C([H])([H])C4=C([H])[C@]([H])(/C([H])=C(\[H])[C@]5([H])C(=O)N([H])[C@@]6([H])C([H])=C([H])C(C([H])([H])[H])=C([H])C6=C5[H])C([H])([H])C([H])([H])[C@]4(C([H])([H])[H])[C@@]3([H])C([H])([H])C([H])([H])[C@@]21C([H])([H])[H]
Estero_109.mol	[H]O[C@@]1([H])C([H])([H])C([H])([H])[C@@]2([H])[C@]3([H])C([H])([H])C([H])([H])C4=C([H])[C@]([H])(/C([H])=C(\[H])[C@]5([H])C(=O)N([H])[C@@]6([H])C([H])=C([H])C([H])=C([H])C6=C5[H])C([H])([H])C([H])([H])[C@]4(C([H])([H])[H])[C@@]3([H])C([H])([H])C([H])([H])[C@@]21C([H])([H])[H]
Estero_110.mol	[H]O[C@@]1([H])C([H])([H])C([H])([H])[C@@]2([H])[C@]3([H])C([H])([H])C([H])([H])C4=C([H])[C@]([H])(/C([H])=C(\[H])[C@]5([H])C(=O)N([H])[C@@]6([H])C([H])=C([H])C(SC([H])([H])[H])=C([H])C6=C5[H])C([H])([H])C([H])([H])[C@]4(C([H])([H])[H])[C@@]3([H])C([H])([H])C([H])([H])[C@@]21C([H])([H])[H]
Estero_111.mol	[H]O[C@@]1([H])C([H])([H])C([H])([H])[C@@]2([H])[C@]3([H])C([H])([H])C([H])([H])C4=C([H])[C@]([H])(/C([H])=C(\[H])[C@]5([H])C(=O)N([H])[C@@]6([H])C([H])=C(Cl)C([H])=C([H])C6=C5[H])C([H])([H])C([H])([H])[C@]4(C([H])([H])[H])[C@@]3([H])C([H])([H])C([H])([H])[C@@]21C([H])([H])[H]

Estero_112.mol	[H]O[C@@]1([H])C([H])([H])C([H])([H])[C@@]2([H])[C@]3([H])C([H])([H])C([H])([H])C4=C([H])[C@]([H])(/C([H])=C(\[H])[C@]5([H])C(=O)N([H])[C@@]6([H])C([H])=C(OC([H])([H])[H])C([H])=C([H])C6=C5[H])C([H])([H])C([H])([H])[C@]4(C([H])([H])[H])[C@@]3([H])C([H])([H])C([H])([H])[C@@]21C([H])([H])[H]
Estero_113.mol	[H]O[C@@]1([H])C([H])([H])C([H])([H])[C@@]2([H])[C@]3([H])C([H])([H])C([H])([H])C4=C([H])[C@]([H])(/C([H])=C(\[H])[C@]5([H])C(=O)N([H])[C@@]6([H])C([H])=C(C(F)(F)F)C([H])=C([H])C6=C5[H])C([H])([H])C([H])([H])[C@]4(C([H])([H])[H])[C@@]3([H])C([H])([H])C([H])([H])[C@@]21C([H])([H])[H]
Estero_114.mol	[H]O[C@@]1([H])C([H])([H])C([H])([H])[C@@]2([H])[C@]3([H])C([H])([H])C([H])([H])C4=C([H])[C@]([H])(/C([H])=C(\[H])[C@]5([H])C(=O)N([H])[C@@]6([H])C([H])=C(C([H])([H])[H])C([H])=C([H])C6=C5[H])C([H])([H])C([H])([H])[C@]4(C([H])([H])[H])[C@@]3([H])C([H])([H])C([H])([H])[C@@]21C([H])([H])[H]
Estero_115.mol	[H]O[C@@]1([H])C([H])([H])C([H])([H])[C@@]2([H])[C@]3([H])C([H])([H])C([H])([H])C4=C([H])[C@]([H])(/C([H])=C(\[H])[C@]5([H])C(=O)N([H])[C@@]6([H])C([H])=C([H])C([H])=C([H])C6=C5[H])C([H])([H])C([H])([H])[C@]4(C([H])([H])[H])[C@@]3([H])C([H])([H])C([H])([H])[C@@]21C([H])([H])[H]
Estero_116.mol	[H]O[C@@]1([H])C([H])([H])C([H])([H])[C@@]2([H])[C@]3([H])C([H])([H])C([H])([H])C4=C([H])[C@]([H])(/C([H])=C(\[H])[C@]5([H])C(=O)N([H])[C@@]6([H])C([H])=C(SC([H])([H])[H])C([H])=C([H])C6=C5[H])C([H])([H])C([H])([H])[C@]4(C([H])([H])[H])[C@@]3([H])C([H])([H])C([H])([H])[C@@]21C([H])([H])[H]
Estero_117.mol	[H]O[C@@]1([H])C([H])([H])C([H])([H])[C@@]2([H])[C@]3([H])C([H])([H])C([H])([H])C4=C([H])[C@]([H])(/C([H])=C(\[H])[C@]5([H])C(=O)N([H])[C@@]6([H])C(Cl)=C([H])C([H])=C([H])C6=C5[H])C([H])([H])C([H])([H])[C@]4(C([H])([H])[H])[C@@]3([H])C([H])([H])C([H])([H])[C@@]21C([H])([H])[H]
Estero_118.mol	[H]O[C@@]1([H])C([H])([H])C([H])([H])[C@@]2([H])[C@]3([H])C([H])([H])C([H])([H])C4=C([H])[C@]([H])(/C([H])=C(\[H])[C@]5([H])C(=O)N([H])[C@@]6([H])C(OC([H])([H])[H])=C([H])C([H])=C([H])C6=C5[H])C([H])([H])C([H])([H])[C@]4(C([H])([H])[H])[C@@]3([H])C([H])([H])C([H])([H])[C@@]21C([H])([H])[H]
Estero_119.mol	[H]O[C@@]1([H])C([H])([H])C([H])([H])[C@@]2([H])[C@]3([H])C([H])([H])C([H])([H])C4=C([H])[C@]([H])(/C([H])=C(\[H])[C@]5([H])C(=O)N([H])[C@]6([H])C(=C5[H])C([H])=C([H])C([H])=C6C(F)(F)F)C([H])([H])C([H])([H])[C@]4(C([H])([H])[H])[C@@]3([H])C([H])([H])C([H])([H])[C@@]21C([H])([H])[H]
Estero_120.mol	[H]O[C@@]1([H])C([H])([H])C([H])([H])[C@@]2([H])[C@]3([H])C([H])([H])C([H])([H])C4=C([H])[C@]([H])(/C([H])=C(\[H])[C@]5([H])C(=O)N([H])[C@]6([H])C(=C5[H])C([H])=C([H])C([H])=C6C([H])([H])[H])C([H])([H])C([H])([H])[C@]4(C([H])([H])[H])[C@@]3([H])C([H])([H])C([H])([H])[C@@]21C([H])([H])[H]
Estero_121.mol	[H]O[C@@]1([H])C([H])([H])C([H])([H])[C@@]2([H])[C@]3([H])C([H])([H])C([H])([H])C4=C([H])[C@]([H])(/C([H])=C(\[H])[C@]5([H])C(=O)N([H])[C@@]6([H])C([H])=C([H])C([H])=C([H])C6=C5[H])C([H])([H])C([H])([H])[C@]4(C([H])([H])[H])[C@@]3([H])C([H])([H])C([H])([H])[C@@]21C([H])([H])[H]
Estero_122.mol	[H]O[C@@]1([H])C([H])([H])C([H])([H])[C@@]2([H])[C@]3([H])C([H])([H])C([H])([H])C4=C([H])[C@]([H])(/C([H])=C(\[H])[C@]5([H])C(=O)N([H])[C@@]6([H])C(SC([H])([H])[H])=C([H])C([H])=C([H])C6=C5[H])C([H])([H])C([H])([H])[C@]4(C([H])([H])[H])[C@@]3([H])C([H])([H])C([H])([H])[C@@]21C([H])([H])[H]
Estero_123.mol	[H]O[C@@]1([H])C([H])([H])C([H])([H])[C@@]2([H])[C@]3([H])C([H])([H])C([H])([H])C4=C([H])[C@]([H])(/C([H])=C(\[H])c5c(OC([H])([H])C([H])([H])[H])nc6c([H])c([H])c(Cl)c([H])c6c5[H])C([H])([H])C([H])([H])[C@]4(C([H])([H])[H])[C@@]3([H])C([H])([H])C([H])([H])[C@@]21C([H])([H])[H]
Estero_124.mol	[H]O[C@@]1([H])C([H])([H])C([H])([H])[C@@]2([H])[C@]3([H])C([H])([H])C([H])([H])C4=C([H])[C@]([H])(/C([H])=C(\[H])c5c(OC([H])([H])C([H])([H])[H])nc6c([H])c([H])c(OC([H])([H])[H])c([H])c6c5[H])C([H])([H])C([H])([H])[C@]4(C([H])([H])[H])[C@@]3([H])C([H])([H])C([H])([H])[C@@]21C([H])([H])[H]

Estero_125.mol	[H]O[C@@]1([H])C([H])([H])C([H])([H])[C@@]2([H])[C@]3([H])C([H])([H])C([H])([H])C4=C([H])C([H])(/C([H])=C(\[H])c5c(OC([H])([H])C([H])([H])[H])nc6c([H])c([H])c(C(F)(F)F)c([H])c6c5[H])C([H])([H])C([H])([H])[C@]4(C([H])([H])[H])[C@@]3([H])C([H])([H])C([H])([H])[C@@]21C([H])([H])[H]
Estero_126.mol	[H]O[C@@]1([H])C([H])([H])C([H])([H])[C@@]2([H])[C@]3([H])C([H])([H])C([H])([H])C4=C([H])C([H])(/C([H])=C(\[H])c5c(OC([H])([H])C([H])([H])[H])nc6c([H])c([H])c(C([H])([H])[H])c([H])c6c5[H])C([H])([H])C([H])([H])[C@]4(C([H])([H])[H])[C@@]3([H])C([H])([H])C([H])([H])[C@@]21C([H])([H])[H]
Estero_127.mol	[H]O[C@@]1([H])C([H])([H])C([H])([H])[C@@]2([H])[C@]3([H])C([H])([H])C([H])([H])C4=C([H])C([H])(/C([H])=C(\[H])c5c(OC([H])([H])C([H])([H])[H])nc6c([H])c([H])c([H])c([H])c6c5[H])C([H])([H])C([H])([H])[C@]4(C([H])([H])[H])[C@@]3([H])C([H])([H])C([H])([H])[C@@]21C([H])([H])[H]
Estero_128.mol	[H]O[C@@]1([H])C([H])([H])C([H])([H])[C@@]2([H])[C@]3([H])C([H])([H])C([H])([H])C4=C([H])C([H])(/C([H])=C(\[H])c5c(OC([H])([H])C([H])([H])[H])nc6c([H])c([H])c(SC([H])([H])[H])c([H])c6c5[H])C([H])([H])C([H])([H])[C@]4(C([H])([H])[H])[C@@]3([H])C([H])([H])C([H])([H])[C@@]21C([H])([H])[H]
Estero_129.mol	[H]O[C@@]1([H])C([H])([H])C([H])([H])[C@@]2([H])[C@]3([H])C([H])([H])C([H])([H])C4=C([H])[C@]([H])(/C([H])=C(\[H])c5c(OC([H])([H])C([H])([H])[H])nc6c([H])c(Cl)c([H])c([H])c6c5[H])C([H])([H])C([H])([H])[C@]4(C([H])([H])[H])[C@@]3([H])C([H])([H])C([H])([H])[C@@]21C([H])([H])[H]
Estero_130.mol	[H]O[C@@]1([H])C([H])([H])C([H])([H])[C@@]2([H])[C@]3([H])C([H])([H])C([H])([H])C4=C([H])[C@]([H])(/C([H])=C(\[H])c5c(OC([H])([H])C([H])([H])[H])nc6c([H])c(OC([H])([H])[H])c([H])c([H])c6c5[H])C([H])([H])C([H])([H])[C@]4(C([H])([H])[H])[C@@]3([H])C([H])([H])C([H])([H])[C@@]21C([H])([H])[H]
Estero_131.mol	[H]O[C@@]1([H])C([H])([H])C([H])([H])[C@@]2([H])[C@]3([H])C([H])([H])C([H])([H])C4=C([H])[C@]([H])(/C([H])=C(\[H])c5c(OC([H])([H])C([H])([H])[H])nc6c([H])c(C(F)(F)F)c([H])c([H])c6c5[H])C([H])([H])C([H])([H])[C@]4(C([H])([H])[H])[C@@]3([H])C([H])([H])C([H])([H])[C@@]21C([H])([H])[H]
Estero_132.mol	[H]O[C@@]1([H])C([H])([H])C([H])([H])[C@@]2([H])[C@]3([H])C([H])([H])C([H])([H])C4=C([H])[C@]([H])(/C([H])=C(\[H])c5c(OC([H])([H])C([H])([H])[H])nc6c([H])c(C([H])([H])[H])c([H])c([H])c6c5[H])C([H])([H])C([H])([H])[C@]4(C([H])([H])[H])[C@@]3([H])C([H])([H])C([H])([H])[C@@]21C([H])([H])[H]
Estero_133.mol	[H]O[C@@]1([H])C([H])([H])C([H])([H])[C@@]2([H])[C@]3([H])C([H])([H])C([H])([H])C4=C([H])[C@]([H])(/C([H])=C(\[H])c5c(OC([H])([H])C([H])([H])[H])nc6c([H])c([H])c([H])c([H])c6c5[H])C([H])([H])C([H])([H])[C@]4(C([H])([H])[H])[C@@]3([H])C([H])([H])C([H])([H])[C@@]21C([H])([H])[H]
Estero_134.mol	[H]O[C@@]1([H])C([H])([H])C([H])([H])[C@@]2([H])[C@]3([H])C([H])([H])C([H])([H])C4=C([H])[C@]([H])(/C([H])=C(\[H])c5c(OC([H])([H])C([H])([H])[H])nc6c([H])c(SC([H])([H])[H])c([H])c([H])c6c5[H])C([H])([H])C([H])([H])[C@]4(C([H])([H])[H])[C@@]3([H])C([H])([H])C([H])([H])[C@@]21C([H])([H])[H]
Estero_135.mol	[H]O[C@@]1([H])C([H])([H])C([H])([H])[C@@]2([H])[C@]3([H])C([H])([H])C([H])([H])C4=C([H])[C@]([H])(/C([H])=C(\[H])c5c(OC([H])([H])C([H])([H])[H])nc6c(Cl)c([H])c([H])c([H])c6c5[H])C([H])([H])C([H])([H])[C@]4(C([H])([H])[H])[C@@]3([H])C([H])([H])C([H])([H])[C@@]21C([H])([H])[H]
Estero_136.mol	[H]O[C@@]1([H])C([H])([H])C([H])([H])[C@@]2([H])[C@]3([H])C([H])([H])C([H])([H])C4=C([H])[C@]([H])(/C([H])=C(\[H])c5c(OC([H])([H])C([H])([H])[H])nc6c(OC([H])([H])[H])c([H])c([H])c([H])c6c5[H])C([H])([H])C([H])([H])[C@]4(C([H])([H])[H])[C@@]3([H])C([H])([H])C([H])([H])[C@@]21C([H])([H])[H]
Estero_137.mol	[H]O[C@@]1([H])C([H])([H])C([H])([H])[C@@]2([H])[C@]3([H])C([H])([H])C([H])([H])C4=C([H])[C@]([H])(/C([H])=C(\[H])c5c(OC([H])([H])C([H])([H])[H])nc6c(C(F)(F)F)c([H])c([H])c([H])c6c5[H])C([H])([H])C([H])([H])[C@]4(C([H])([H])[H])[C@@]3([H])C([H])([H])C([H])([H])[C@@]21C([H])([H])[H]

Estero_138.mol	[H]O[C@@]1([H])C([H])([H])C([H])([H])[C@@]2([H])[C@]3([H])C([H])([H])C([H])([H])C4=C([H])[C@]([H])(/C([H])=C(\[H])c5c(OC([H])([H])C([H])([H])[H])nc6c(C([H])([H])[H])c([H])c([H])c([H])c6c5[H])C([H])([H])C([H])([H])[C@]4(C([H])([H])[H])[C@@]3([H])C([H])([H])C([H])([H])[C@@]21C([H])([H])[H]
Estero_139.mol	[H]O[C@@]1([H])C([H])([H])C([H])([H])[C@@]2([H])[C@]3([H])C([H])([H])C([H])([H])C4=C([H])[C@]([H])(/C([H])=C(\[H])c5c(OC([H])([H])C([H])([H])[H])nc6c([H])c([H])c([H])c([H])c6c5[H])C([H])([H])C([H])([H])[C@]4(C([H])([H])[H])[C@@]3([H])C([H])([H])C([H])([H])[C@@]21C([H])([H])[H]
Estero_140.mol	[H]O[C@@]1([H])C([H])([H])C([H])([H])[C@@]2([H])[C@]3([H])C([H])([H])C([H])([H])C4=C([H])[C@]([H])(/C([H])=C(\[H])c5c(OC([H])([H])C([H])([H])[H])nc6c(SC([H])([H])[H])c([H])c([H])c([H])c6c5[H])C([H])([H])C([H])([H])[C@]4(C([H])([H])[H])[C@@]3([H])C([H])([H])C([H])([H])[C@@]21C([H])([H])[H]
Estero_141.mol	[H]O[C@@]1([H])C([H])([H])C([H])([H])[C@@]2([H])[C@]3([H])C([H])([H])C([H])([H])C4=C([H])[C@]([H])(C5=NN(C(=O)C([H])([H])[H])[C@@]([H])(C6=C([H])[C@]7([H])C([H])=C(Cl)C([H])=C([H])[C@@]7([H])N([H])C6=O)C5([H])[H])C([H])([H])C([H])([H])[C@]4(C([H])([H])[H])[C@@]3([H])C([H])([H])C([H])([H])[C@@]21C([H])([H])[H]
Estero_142.mol	[H]O[C@@]1([H])C([H])([H])C([H])([H])[C@@]2([H])[C@]3([H])C([H])([H])C([H])([H])C4=C([H])[C@]([H])(C5=NN(C(=O)C([H])([H])[H])[C@@]([H])(C6=C([H])[C@]7([H])C([H])=C(OC([H])([H])[H])C([H])=C([H])[C@@]7([H])N([H])C6=O)C5([H])[H])C([H])([H])C([H])([H])[C@]4(C([H])([H])[H])[C@@]3([H])C([H])([H])C([H])([H])[C@@]21C([H])([H])[H]
Estero_143.mol	[H]O[C@@]1([H])C([H])([H])C([H])([H])[C@@]2([H])[C@]3([H])C([H])([H])C([H])([H])C4=C([H])[C@]([H])(C5=NN(C(=O)C([H])([H])[H])[C@@]([H])(C6=C([H])[C@]7([H])C([H])=C(C(F)(F)F)C([H])=C([H])[C@@]7([H])N([H])C6=O)C5([H])[H])C([H])([H])C([H])([H])[C@]4(C([H])([H])[H])[C@@]3([H])C([H])([H])C([H])([H])[C@@]21C([H])([H])[H]
Estero_144.mol	[H]O[C@@]1([H])C([H])([H])C([H])([H])[C@@]2([H])[C@]3([H])C([H])([H])C([H])([H])C4=C([H])[C@]([H])(C5=NN(C(=O)C([H])([H])[H])[C@@]([H])(C6=C([H])[C@]7([H])C([H])=C(C([H])([H])[H])C([H])=C([H])[C@@]7([H])N([H])C6=O)C5([H])[H])C([H])([H])C([H])([H])[C@]4(C([H])([H])[H])[C@@]3([H])C([H])([H])C([H])([H])[C@@]21C([H])([H])[H]
Estero_145.mol	[H]O[C@@]1([H])C([H])([H])C([H])([H])[C@@]2([H])[C@]3([H])C([H])([H])C([H])([H])C4=C([H])[C@]([H])(C5=NN(C(=O)C([H])([H])[H])[C@@]([H])(C6=C([H])[C@]7([H])C([H])=C([H])C([H])=C([H])[C@@]7([H])N([H])C6=O)C5([H])[H])C([H])([H])C([H])([H])[C@]4(C([H])([H])[H])[C@@]3([H])C([H])([H])C([H])([H])[C@@]21C([H])([H])[H]
Estero_146.mol	[H]O[C@@]1([H])C([H])([H])C([H])([H])[C@@]2([H])[C@]3([H])C([H])([H])C([H])([H])C4=C([H])[C@]([H])(C5=NN(C(=O)C([H])([H])[H])[C@@]([H])(C6=C([H])[C@]7([H])C([H])=C(SC([H])([H])[H])C([H])=C([H])[C@@]7([H])N([H])C6=O)C5([H])[H])C([H])([H])C([H])([H])[C@]4(C([H])([H])[H])[C@@]3([H])C([H])([H])C([H])([H])[C@@]21C([H])([H])[H]
Estero_147.mol	[H]O[C@@]1([H])C([H])([H])C([H])([H])[C@@]2([H])[C@]3([H])C([H])([H])C([H])([H])C4=C([H])[C@]([H])(C5=NN(C(=O)C([H])([H])[H])[C@@]([H])(C6=C([H])[C@]7([H])C([H])=C([H])C(Cl)=C([H])[C@@]7([H])N([H])C6=O)C5([H])[H])C([H])([H])C([H])([H])[C@]4(C([H])([H])[H])[C@@]3([H])C([H])([H])C([H])([H])[C@@]21C([H])([H])[H]
Estero_148.mol	[H]O[C@@]1([H])C([H])([H])C([H])([H])[C@@]2([H])[C@]3([H])C([H])([H])C([H])([H])C4=C([H])[C@]([H])(C5=NN(C(=O)C([H])([H])[H])[C@@]([H])(C6=C([H])[C@]7([H])C([H])=C([H])C(OC([H])([H])[H])=C([H])[C@@]7([H])N([H])C6=O)C5([H])[H])C([H])([H])C([H])([H])[C@]4(C([H])([H])[H])[C@@]3([H])C([H])([H])C([H])([H])[C@@]21C([H])([H])[H]
Estero_149.mol	[H]O[C@@]1([H])C([H])([H])C([H])([H])[C@@]2([H])[C@]3([H])C([H])([H])C([H])([H])C4=C([H])[C@]([H])(C5=NN(C(=O)C([H])([H])[H])[C@@]([H])(C6=C([H])[C@]7([H])C([H])=C([H])C(C(F)(F)F)=C([H])[C@@]7([H])N([H])C6=O)C5([H])[H])C([H])([H])C([H])([H])[C@]4(C([H])([H])[H])[C@@]3([H])C([H])([H])C([H])([H])[C@@]21C([H])([H])[H]

Estero_ 150.mol	[H]O[C@@]1([H])C([H])([H])C([H])([H])[C@@]2([H])[C@]3([H])C([H])([H])C([H])([H])C4=C([H])[C@]([H])(C5=NN(C(=O)C([H])([H])[H])[C@@]([H])(C6=C([H])[C@]7([H])C([H])=C([H])C(C([H])([H])[H])=C([H])[C@@]7([H])N([H])C6=O)C5([H])[H])C([H])([H])C([H])([H])[C@]4(C([H])([H])[H])[C@@]3([H])C([H])([H])C([H])([H])[C@@]21C([H])([H])[H]
Estero_ 151.mol	[H]O[C@@]1([H])C([H])([H])C([H])([H])[C@@]2([H])[C@]3([H])C([H])([H])C([H])([H])C4=C([H])[C@]([H])(C5=NN(C(=O)C([H])([H])[H])[C@@]([H])(C6=C([H])[C@]7([H])C([H])=C([H])C([H])=C([H])[C@@]7([H])N([H])C6=O)C5([H])[H])C([H])([H])C([H])([H])[C@]4(C([H])([H])[H])[C@@]3([H])C([H])([H])C([H])([H])[C@@]21C([H])([H])[H]
Estero_ 152.mol	[H]O[C@@]1([H])C([H])([H])C([H])([H])[C@@]2([H])[C@]3([H])C([H])([H])C([H])([H])C4=C([H])[C@]([H])(C5=NN(C(=O)C([H])([H])[H])[C@@]([H])(C6=C([H])[C@]7([H])C([H])=C([H])C(SC([H])([H])[H])=C([H])[C@@]7([H])N([H])C6=O)C5([H])[H])C([H])([H])C([H])([H])[C@]4(C([H])([H])[H])[C@@]3([H])C([H])([H])C([H])([H])[C@@]21C([H])([H])[H]
Estero_ 153.mol	[H]O[C@@]1([H])C([H])([H])C([H])([H])[C@@]2([H])[C@]3([H])C([H])([H])C([H])([H])C4=C([H])[C@]([H])(C5=NN(C(=O)C([H])([H])[H])[C@@]([H])(C6=C([H])[C@]7([H])C([H])=C([H])C([H])=C(Cl)[C@@]7([H])N([H])C6=O)C5([H])[H])C([H])([H])C([H])([H])[C@]4(C([H])([H])[H])[C@@]3([H])C([H])([H])C([H])([H])[C@@]21C([H])([H])[H]
Estero_ 154.mol	[H]O[C@@]1([H])C([H])([H])C([H])([H])[C@@]2([H])[C@]3([H])C([H])([H])C([H])([H])C4=C([H])[C@]([H])(C5=NN(C(=O)C([H])([H])[H])[C@@]([H])(C6=C([H])[C@]7([H])C([H])=C([H])C([H])=C(OC([H])([H])[H])[C@@]7([H])N([H])C6=O)C5([H])[H])C([H])([H])C([H])([H])[C@]4(C([H])([H])[H])[C@@]3([H])C([H])([H])C([H])([H])[C@@]21C([H])([H])[H]
Estero_ 155.mol	[H]O[C@@]1([H])C([H])([H])C([H])([H])[C@@]2([H])[C@]3([H])C([H])([H])C([H])([H])C4=C([H])[C@]([H])(C5=NN(C(=O)C([H])([H])[H])[C@@]([H])(C6=C([H])[C@]7([H])C([H])=C([H])C([H])=C(C(F)(F)F)[C@@]7([H])N([H])C6=O)C5([H])[H])C([H])([H])C([H])([H])[C@]4(C([H])([H])[H])[C@@]3([H])C([H])([H])C([H])([H])[C@@]21C([H])([H])[H]
Estero_ 156.mol	[H]O[C@@]1([H])C([H])([H])C([H])([H])[C@@]2([H])[C@]3([H])C([H])([H])C([H])([H])C4=C([H])[C@]([H])(C5=NN(C(=O)C([H])([H])[H])[C@@]([H])(C6=C([H])[C@]7([H])C([H])=C([H])C([H])=C(C([H])([H])[H])[C@@]7([H])N([H])C6=O)C5([H])[H])C([H])([H])C([H])([H])[C@]4(C([H])([H])[H])[C@@]3([H])C([H])([H])C([H])([H])[C@@]21C([H])([H])[H]
Estero_ 157.mol	[H]O[C@@]1([H])C([H])([H])C([H])([H])[C@@]2([H])[C@]3([H])C([H])([H])C([H])([H])C4=C([H])[C@]([H])(C5=NN(C(=O)C([H])([H])[H])[C@@]([H])(C6=C([H])[C@]7([H])C([H])=C([H])C([H])=C([H])[C@@]7([H])N([H])C6=O)C5([H])[H])C([H])([H])C([H])([H])[C@]4(C([H])([H])[H])[C@@]3([H])C([H])([H])C([H])([H])[C@@]21C([H])([H])[H]
Estero_ 158.mol	[H]O[C@@]1([H])C([H])([H])C([H])([H])[C@@]2([H])[C@]3([H])C([H])([H])C([H])([H])C4=C([H])[C@]([H])(C5=NN(C(=O)C([H])([H])[H])[C@@]([H])(C6=C([H])[C@]7([H])C([H])=C([H])C([H])=C(SC([H])([H])[H])[C@@]7([H])N([H])C6=O)C5([H])[H])C([H])([H])C([H])([H])[C@]4(C([H])([H])[H])[C@@]3([H])C([H])([H])C([H])([H])[C@@]21C([H])([H])[H]
Estero_ 163.mol	[H]O[C@@]1([H])C([H])=C2C([H])([H])C([H])([H])[C@@]3(C([H])([H])[H])[C@]4([H])C([H])([H])C([H])([H])[C@]([H])(C([H])=O)[C@@]4(C([H])([H])[H])C([H])([H])C([H])([H])[C@]3([H])[C@@]2(C([H])([H])[H])C([H])([H])C1([H])[H]
Estero_ 164.mol	[H]O[C@@]1([H])C([H])=C2C([H])([H])C([H])([H])[C@@]3(C([H])([H])[H])[C@]4([H])C([H])([H])C([H])([H])[C@]([H])(C(=O)SC([H])([H])[H])[C@@]4(C([H])([H])[H])C([H])([H])C([H])([H])[C@]3([H])[C@@]2(C([H])([H])[H])C([H])([H])C1([H])[H]
Estero_ 173.mol	[H]O[C@@]1([H])C([H])=C2C([H])([H])[C@]([H])(Cl)[C@]3(C([H])([H])[H])[C@]([H])(C([H])([H])C([H])([H])[C@@]4(C([H])([H])[H])[C@@]3([H])C([H])([H])C([H])([H])[C@]4([H])C(=O)C([H])([H])[H])[C@@]2(C([H])([H])[H])C([H])([H])C1([H])[H]
Estero_ 175.mol	[H]O[C@@]1([H])C([H])=C2C([H])([H])[C@]([H])(C(F)(F)F)[C@]3(C([H])([H])[H])[C@]([H])(C([H])([H])C([H])([H])[C@@]4(C([H])([H])[H])[C@@]3([H])C([H])([H])C([H])([H])[C@]4([H])C(=O)C([H])([H])[H])[C@@]2(C([H])([H])[H])C([H])([H])C1([H])[H]

Estero_176.mol	[H]O[C@@]1([H])C([H])=C2C([H])([H])[C@]([H])(C([H])([H])[H])[C@]3(C([H])([H])[H])[C@]([H])(C([H])([H])C([H])([H])[C@@]4(C([H])([H])[H])[C@@]3([H])C([H])([H])C([H])([H])[C@]4([H])C(=O)C([H])([H])[H])[C@@]2(C([H])([H])[H])C([H])([H])C1([H])[H]
Estero_177.mol	[H]O[C@@]1([H])C([H])=C2C([H])([H])C([H])([H])[C@@]3(C([H])([H])[H])[C@]4([H])C([H])([H])C([H])([H])[C@]([H])(C(=O)C([H])([H])[H])[C@@]4(C([H])([H])[H])C([H])([H])C([H])([H])[C@]3([H])[C@@]2(C([H])([H])[H])C([H])([H])C1([H])[H]
Estero_178.mol	[H]O[C@@]1([H])C([H])=C2C([H])([H])[C@]([H])(SC([H])([H])[H])[C@]3(C([H])([H])[H])[C@]([H])(C([H])([H])C([H])([H])[C@@]4(C([H])([H])[H])[C@@]3([H])C([H])([H])C([H])([H])[C@]4([H])C(=O)C([H])([H])[H])[C@@]2(C([H])([H])[H])C([H])([H])C1([H])[H]
Estero_179.mol	[H]O[C@]1([H])[C@]2(C([H])([H])[H])[C@]([H])(C([H])([H])C([H])([H])[C@@]3(C([H])([H])[H])[C@@]2([H])C([H])([H])C([H])([H])[C@]3([H])C(=O)C([H])([H])[H])[C@]2(C([H])([H])[H])C(=C([H])[C@]([H])(O[H])C([H])([H])C2([H])[H])[C@@]1([H])O[H]
Estero_180.mol	[H]/N=C1/C([H])([H])C2=C([H])[C@]([H])(O[H])C([H])([H])C([H])([H])[C@]2(C([H])([H])[H])[C@@]2([H])C([H])([H])C([H])([H])[C@@]3(C([H])([H])[H])[C@@]([H])(C([H])([H])C([H])([H])[C@]3([H])C(=O)C([H])([H])[H])[C@]12C([H])([H])[H]
Estero-181.mol	[H]Oc1c([H])c([H])c2c(c1[H])C([H])([H])C([H])([H])[C@@]1([H])[C@]3([H])C([H])([H])/C(=C(/[H])c4c(Cl)nc5c([H])c([H])c([H])c([H])c5c4[H])C(=O)[C@@]3(C([H])([H])[H])C([H])([H])C([H])([H])[C@]21[H]
Estero-182.mol	[H]Oc1c([H])c([H])c2c(c1[H])C([H])([H])C([H])([H])[C@@]1([H])[C@]3([H])C([H])([H])/C(=C(/[H])c4c(Cl)nc5c([H])c([H])c(C([H])([H])[H])c([H])c5c4[H])C(=O)[C@@]3(C([H])([H])[H])C([H])([H])C([H])([H])[C@]21[H]
Estero-183.mol	[H]Oc1c([H])c([H])c2c(c1[H])C([H])([H])C([H])([H])[C@@]1([H])[C@]3([H])C([H])([H])/C(=C(/[H])c4c(Cl)nc5c([H])c([H])c(OC([H])([H])[H])c([H])c5c4[H])C(=O)[C@@]3(C([H])([H])[H])C([H])([H])C([H])([H])[C@]21[H]
Estero-184.mol	[H]Oc1c([H])c([H])c2c(c1[H])C([H])([H])C([H])([H])[C@@]1([H])[C@]3([H])C([H])([H])/C(=C(/[H])c4c(Cl)nc5c([H])c(OC([H])([H])[H])c(OC([H])([H])[H])c([H])c5c4[H])C(=O)[C@@]3(C([H])([H])[H])C([H])([H])C([H])([H])[C@]21[H]
Estero-185.mol	[H]Oc1c([H])c([H])c2c(c1[H])C([H])([H])C([H])([H])[C@@]1([H])[C@]3([H])C([H])([H])/C(=C(/[H])c4c(Cl)nc5c([H])c(OC([H])([H])[H])c([H])c([H])c5c4[H])C(=O)[C@@]3(C([H])([H])[H])C([H])([H])C([H])([H])[C@]21[H]
Estero-188.mol	[H]Oc1c([H])c([H])c2c(c1[H])C([H])([H])C([H])([H])[C@@]1([H])[C@]3([H])C([H])([H])/C(=C(/[H])c4c(Cl)nc5c(C([H])([H])[H])c([H])c([H])c([H])c5c4[H])C(=O)[C@@]3(C([H])([H])[H])C([H])([H])C([H])([H])[C@]21[H]
Estero-189.mol	[H]Oc1c([H])c([H])c2c(c1[H])C([H])([H])C([H])([H])[C@@]1([H])[C@]3([H])C([H])([H])/C(=C(/[H])c4c(Cl)nc5c([H])c([H])c(C(F)(F)F)c([H])c5c4[H])C(=O)[C@@]3(C([H])([H])[H])C([H])([H])C([H])([H])[C@]21[H]
Estero-190.mol	[H]Oc1c([H])c([H])c2c(c1[H])C([H])([H])C([H])([H])[C@@]1([H])[C@]3([H])C([H])([H])/C(=C(/[H])c4c([H])c5c([H])c([H])c([H])c([H])c5n([H])c4=O)C(=O)[C@@]3(C([H])([H])[H])C([H])([H])C([H])([H])[C@]21[H]
Estero-191.mol	[H]Oc1c([H])c([H])c2c(c1[H])C([H])([H])C([H])([H])[C@@]1([H])[C@]3([H])C([H])([H])/C(=C(/[H])c4c([H])c5c([H])c(C([H])([H])[H])c([H])c([H])c5n([H])c4=O)C(=O)[C@@]3(C([H])([H])[H])C([H])([H])C([H])([H])[C@]21[H]
Estero-193.mol	[H]Oc1c([H])c([H])c2c(c1[H])C([H])([H])C([H])([H])[C@@]1([H])[C@]3([H])C([H])([H])/C(=C(/[H])c4c([H])c5c([H])c(OC([H])([H])[H])c(OC([H])([H])[H])c([H])c5n([H])c4=O)C(=O)[C@@]3(C([H])([H])[H])C([H])([H])C([H])([H])[C@]21[H]
Estero-194.mol	[H]Oc1c([H])c([H])c2c(c1[H])C([H])([H])C([H])([H])[C@@]1([H])[C@]3([H])C([H])([H])/C(=C(/[H])c4c([H])c5c([H])c([H])c(OC([H])([H])[H])c([H])c5n([H])c4=O)C(=O)[C@@]3(C([H])([H])[H])C([H])([H])C([H])([H])[C@]21[H]
Estero-195.mol	[H]Oc1c([H])c([H])c2c(c1[H])C([H])([H])C([H])([H])[C@@]1([H])[C@]3([H])C([H])([H])/C(=C(/[H])c4c([H])c5c([H])c(F)c([H])c([H])c5n([H])c4=O)C(=O)[C@@]3(C([H])([H])[H])C([H])([H])C([H])([H])[C@]21[H]

Estero-196.mol	[H]Oc1c([H])c([H])c2c(c1[H])C([H])([H])C([H])([H])[C@@]1([H])[C@]3([H])C([H])([H])/C(=C(/[H])c4c([H])c5c([H])c(Cl)c([H])c([H])c5n([H])c4=O)C(=O)[C@@]3(C([H])([H])[H])C([H])([H])C([H])([H])[C@]21[H]
Estero-197.mol	[H]Oc1c([H])c([H])c2c(c1[H])C([H])([H])C([H])([H])[C@@]1([H])[C@]3([H])C([H])([H])/C(=C(/[H])c4c([H])c5c([H])c([H])c([H])c(C([H])([H])[H])c5n([H])c4=O)C(=O)[C@@]3(C([H])([H])[H])C([H])([H])C([H])([H])[C@]21[H]
Estero-198.mol	[H]Oc1c([H])c([H])c2c(c1[H])C([H])([H])C([H])([H])[C@@]1([H])[C@]3([H])C([H])([H])/C(=C(/[H])c4c([H])c5c([H])c(C(F)(F)F)c([H])c([H])c5n([H])c4=O)C(=O)[C@@]3(C([H])([H])[H])C([H])([H])C([H])([H])[C@]21[H]
Estero-199.mol	[H]C1=C2C([H])([H])C([H])([H])[C@@]3([H])[C@]4([H])C([H])([H])C([H])([H])[C@]([H])(C(=O)/C([H])=C(\[H])c5c(Cl)nc6c([H])c([H])c([H])c([H])c6c5[H])[C@@]4(C([H])([H])[H])C([H])([H])C([H])([H])[C@]3([H])[C@@]2(C([H])([H])[H])C([H])([H])C([H])([H])C1=O
Estero-200.mol	[H]C1=C2C([H])([H])C([H])([H])[C@@]3([H])[C@]4([H])C([H])([H])C([H])([H])[C@]([H])(C(=O)/C([H])=C(\[H])c5c(Cl)nc6c([H])c([H])c(C([H])([H])[H])c([H])c6c5[H])[C@@]4(C([H])([H])[H])C([H])([H])C([H])([H])[C@]3([H])[C@@]2(C([H])([H])[H])C([H])([H])C([H])([H])C1=O
Estero-201.mol	[H]C1=C2C([H])([H])C([H])([H])[C@@]3([H])[C@]4([H])C([H])([H])C([H])([H])[C@]([H])(C(=O)/C([H])=C(\[H])c5c(Cl)nc6c([H])c([H])c(OC([H])([H])[H])c([H])c6c5[H])[C@@]4(C([H])([H])[H])C([H])([H])C([H])([H])[C@]3([H])[C@@]2(C([H])([H])[H])C([H])([H])C([H])([H])C1=O
Estero-202.mol	[H]C1=C2C([H])([H])C([H])([H])[C@@]3([H])[C@]4([H])C([H])([H])C([H])([H])[C@]([H])(C(=O)/C([H])=C(\[H])c5c(Cl)nc6c([H])c(OC([H])([H])[H])c(OC([H])([H])[H])c([H])c6c5[H])[C@@]4(C([H])([H])[H])C([H])([H])C([H])([H])[C@]3([H])[C@@]2(C([H])([H])[H])C([H])([H])C([H])([H])C1=O
Estero-251.mol	[H]C1=C2C([H])([H])[C@@]([H])(OC(=O)C([H])([H])[H])C([H])([H])C([H])([H])[C@]2(C([H])([H])[H])[C@@]2(C([H])([H])[H])C([H])([H])C([H])([H])[C@@]3(C([H])([H])[H])[C@@]([H])(C([H])([H])C([H])([H])[C@]3([H])C(=O)/C([H])=C(\[H])c3c([H])c4c([H])c(Cl)c([H])c([H])c4n([H])c3=O)[C@]2([H])C1([H])[H]
Estero-252.mol	[H]C1=C2C([H])([H])[C@@]([H])(OC(=O)C([H])([H])[H])C([H])([H])C([H])([H])[C@]2(C([H])([H])[H])[C@@]2(C([H])([H])[H])C([H])([H])C([H])([H])[C@@]3(C([H])([H])[H])[C@@]([H])(C([H])([H])C([H])([H])[C@]3([H])C(=O)/C([H])=C(\[H])c3c([H])c4c([H])c(C(F)(F)F)c([H])c([H])c4n([H])c3=O)[C@]2([H])C1([H])[H]
Estero-253.mol	[H]c1c([H])c2c(c([H])c1OC([H])([H])c1c(Cl)nc3c([H])c([H])c(C([H])([H])[H])c([H])c3c1[H])C([H])([H])C([H])([H])[C@@]1([H])[C@]3([H])C([H])([H])C([H])([H])C(=O)[C@@]3(C([H])([H])[H])C([H])([H])C([H])([H])[C@]21[H]
Estero-256.mol	[H]c1c([H])c2c(c([H])c1OC([H])([H])c1c(Cl)nc3c([H])c([H])c(F)c([H])c3c1[H])C([H])([H])C([H])([H])[C@@]1([H])[C@@]3([H])C([H])([H])C([H])([H])C(=O)[C@@]3(C([H])([H])[H])C([H])([H])C([H])([H])[C@@]21[H]
Estero-257.mol	[H]c1c([H])c2c(c([H])c1OC([H])([H])c1c(Cl)nc3c([H])c(OC([H])([H])[H])c([H])c([H])c3c1[H])C([H])([H])C([H])([H])[C@@]1([H])[C@@]3([H])C([H])([H])C([H])([H])C(=O)[C@@]3(C([H])([H])[H])C([H])([H])C([H])([H])[C@@]21[H]
Estero-258.mol	[H]c1c([H])c2c(c([H])c1OC([H])([H])c1c(Cl)nc3c([H])c(OC([H])([H])[H])c(OC([H])([H])[H])c([H])c3c1[H])C([H])([H])C([H])([H])[C@@]1([H])[C@@]3([H])C([H])([H])C([H])([H])C(=O)[C@@]3(C([H])([H])[H])C([H])([H])C([H])([H])[C@]21[H]
Estero-259.mol	[H]c1c([H])c2c(c([H])c1OC([H])([H])c1c(Cl)nc3c([H])c(OC([H])([H])[H])c([H])c([H])c3c1[H])C([H])([H])C([H])([H])[C@@]1([H])[C@@]3([H])C([H])([H])C([H])([H])C(=O)[C@@]3(C([H])([H])[H])C([H])([H])C([H])([H])[C@@]21[H]
Estero-260.mol	[H]c1c([H])c2c(c([H])c1OC([H])([H])c1c(Cl)nc3c([H])c([H])c(C(F)(F)F)c([H])c3c1[H])C([H])([H])C([H])([H])[C@@]1([H])[C@@]3([H])C([H])([H])C([H])([H])C(=O)[C@@]3(C([H])([H])[H])C([H])([H])C([H])([H])[C@@]21[H]

Estero-262.mol	[H]c1c([H])c2c(c([H])c1OC([H])([H])c1c(Cl)nc3c([H])c([H])c(Cl)c([H])c3c1[H])C([H])([H])C([H])([H])[C@@]1([H])[C@@]3([H])C([H])([H])C([H])([H])C(=O)[C@@]3(C([H])([H])[H])C([H])([H])C([H])([H])[C@@]21[H]
Estero-266.mol	[H]c1c([H])c2c(c([H])c1OC([H])([H])c1c([H])c3c([H])c(C(F)(F)F)c([H])c([H])c3n([H])c1=O)C([H])([H])C([H])([H])[C@@]1([H])[C@@]3([H])C([H])([H])C([H])([H])C(=O)[C@@]3(C([H])([H])[H])C([H])([H])C([H])([H])[C@]21[H]

Supplementary Material, S3. Python code for decision-making matrix.

```
#!/usr/bin/env
python3 """

improved_rank_compounds_v2.py

Rank compounds by combining docking scores and biological activity.

Key Features
```

- Flexible delimiter detection (comma, tab, semicolon) or explicit `--sep`.
- Custom weights for docking vs. activity (`--dock-weight`, `--act-weight`).
- Custom activity column name (`--activity-col`).
- Column exclusion list (`--exclude`).
- Adds **Rank** column (1 = best) after scoring.
- Prints a nicely formatted top‑N ranking table in the console using `tabulate`.
- Saves the full ranked table with **Rank** and **Score** to CSV or Excel.
- Robust numeric conversion and detailed error messages.
- Works on Python ≥3.8; requires pandas and tabulate.

```
Install extras:

    pip install pandas tabulate openpyxl

Example

    python improved_rank_compounds_v2.py data.csv -o ranked.xlsx --top 15

"""

import argparse
import sys

from pathlib import Path

from typing import List, Optional

import pandas as pd

from tabulate import tabulate
```

```
def infer_separator(file_path: Path) -> str:

    """Infer the separator (delimiter) used in a text table."""

    with file_path.open("r", encoding="utf-8", errors="ignore") as fh:
        sample = fh.read(4096)

    if "\t" in sample and "," not in sample:
        return "\t"

    if ";" in sample and sample.count(";") > sample.count(","):
        return ";"

    return "," # default to comma

def
    compute_score(
    row:
    pd.Series,

    docking_cols:
    List[str],
    activity_col: str,
    dock_weight: float,
    act_weight: float,

) -> float:

    """Compute composite score
```

Supplementary Material Figure S1.

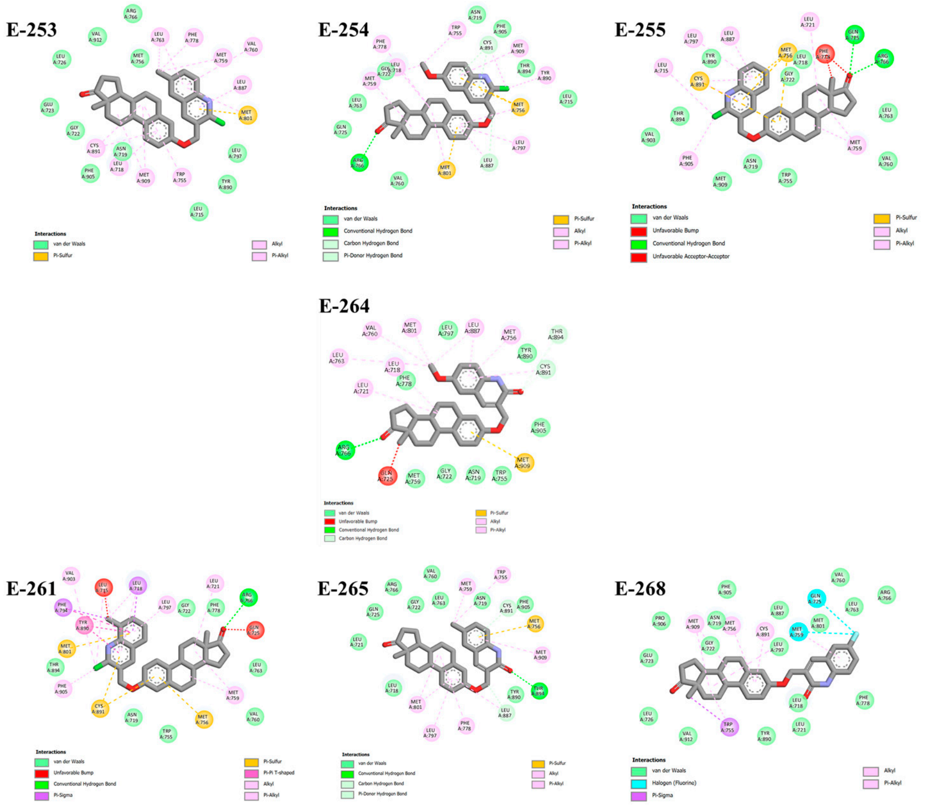

Figure S1. Twodimensional interaction diagrams of the designed steroidal ligands bound to the progesteronereceptor ligandbinding domain (PDB 2w8y). Interaction types are colourcoded as follows: van der Waals (light green), conventional hydrogen bond (bright green), carbonhydrogen bond (greygreen), πdonor hydrogen bond (pale green), πsulfur (yellow), alkyl (pink), πalkyl (purple), halogen (cyan) and unfavourable contacts (red).

Material Supplementary: Figure S2.

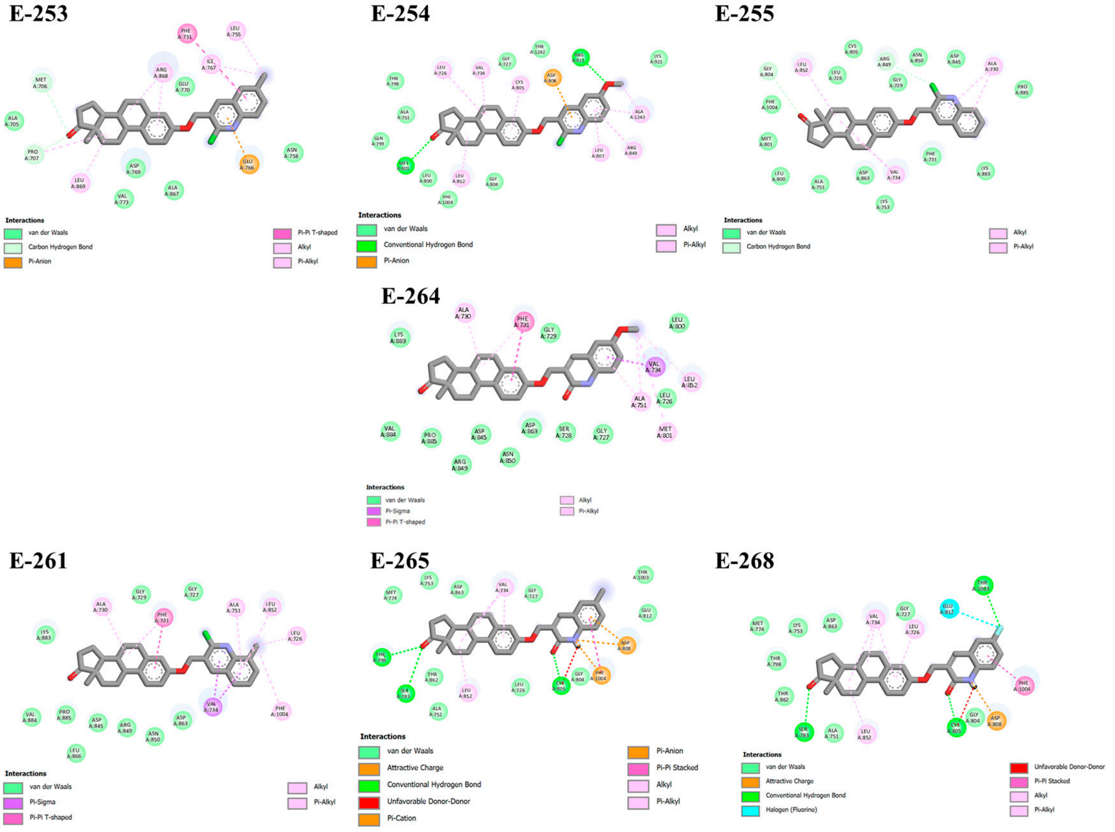

Figure S2. Twodimensional interaction diagrams of the designed steroidal ligands with the HER2 tyrosinekinase domain (PDB 7jxh). Interaction types are colorcoded as follows: van der Waals (green), conventional hydrogen bond (pink), ππ / πalkyl (purple), πanion (orange), attractive charge (blue), halogen (cyan), and unfavorable contacts (red).

Material Supplementary. Figure S3.

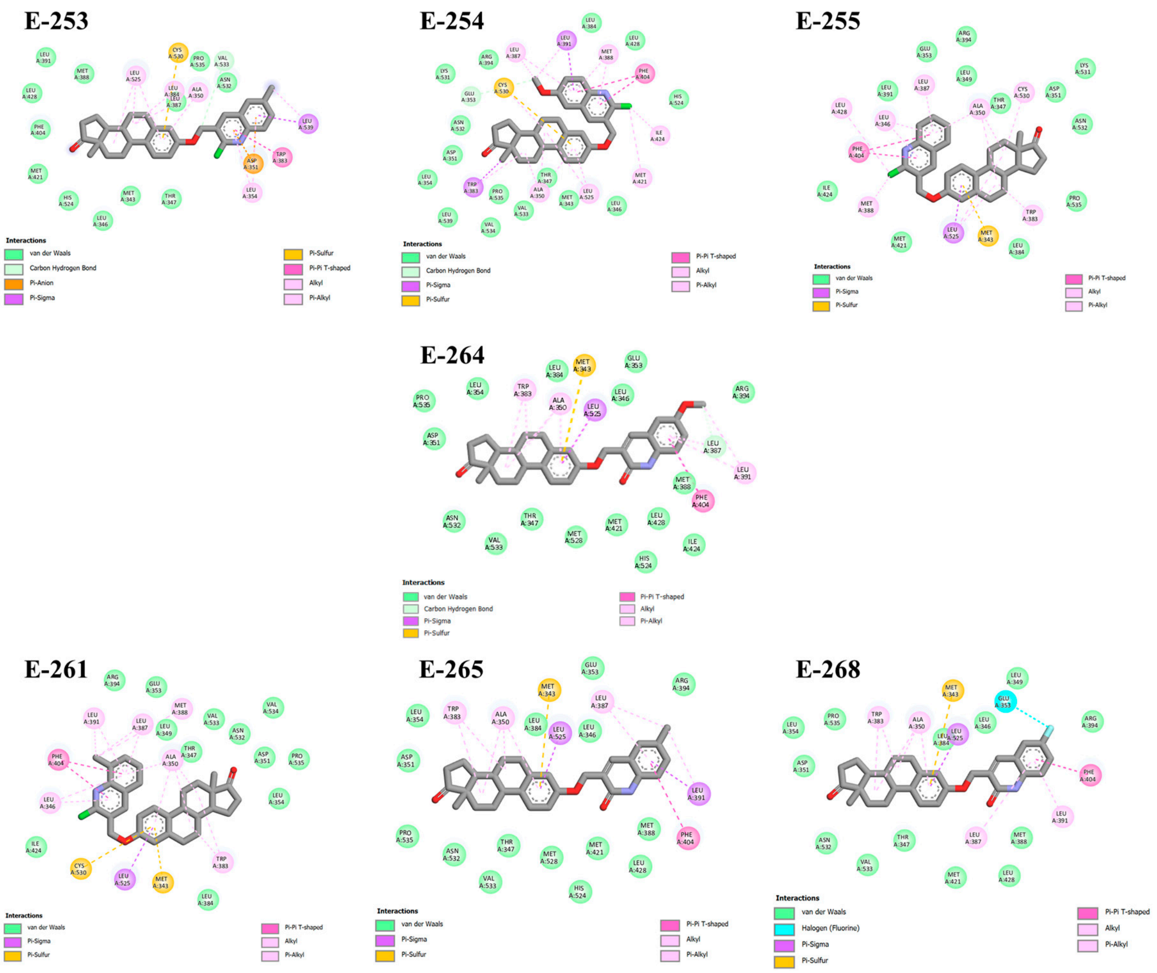

Figure S3. Twodimensional interaction diagrams of the designed steroidal ligands bound to the ERα ligandbinding domain (PDB 6vjd). Interaction types are colourcoded as follows: van der Waals (green), carbon–hydrogen bond (greygreen), conventional hydrogen bond (bright green), ππ / πalkyl (lavender), ππ Tshaped (magenta), πanion (orange), πsigma (purple), πsulfur (yellow), halogen (cyan), alkyl (pink) and unfavourable contacts (red).
